# South Asia-specific adaptation of Mediterranean diet principles: a mixed-methods review for practical and sustainable dietary habits

**DOI:** 10.3389/fnut.2025.1719686

**Published:** 2025-12-23

**Authors:** Daniele Spadaccini, Arun Chandran, Filipa Patricia Gonçalves Correia, Helia Janji, Carola Ciamparini, Sabrina Tini, Marina Caputo, Paolo Marzullo, Gianluca Aimaretti, Flavia Prodam

**Affiliations:** 1 Department of Health Sciences, University of Piemonte Orientale, Novara, Italy; 2 Department of Biology, University of North Dakota, Grand Forks, ND, United States; 3 Department of Translational Medicine and Endocrinology, University of Piemonte Orientale, Novara, Italy

**Keywords:** India, nutrient intake, vegetarianism, non-communicable diseases, food systems

## Abstract

South Asia, home to nearly 2 billion people, faces a dual burden of persistent malnutrition and rapidly rising rates of obesity, type 2 diabetes, and cardiovascular diseases. Dietary patterns are dominated by refined cereals, low intake of fruits, vegetables, and whole grains, limited protein quality, and imbalanced fat composition, compounded by cultural practices and the growing penetration of ultra-processed foods. This mixed-method review systematically synthesized dietary intake data and contextual barriers to evaluate the transferability of Mediterranean Diet (MD) principles to South Asia. Unlike broader continental frameworks, our approach integrates local foods, cultural traditions, and environmental realities to design two region-specific dietary pyramids for vegetarian and non-vegetarian populations. The adapted model emphasizes higher consumption of whole grains, legumes, fruits, and vegetables, the inclusion of affordable high-quality protein sources, and a balanced use of locally available fats, while placing sweets and ultra-processed foods at the top of the pyramid with clear limits. Beyond nutrient adequacy, our analysis highlights structural barriers, economic affordability, entrenched food traditions, limited nutritional awareness, environmental pressures, and food safety challenges that must be addressed to ensure feasibility. Policy action, nutrition education, women’s empowerment, climate-smart agriculture, and fortification strategies emerge as key enablers for a sustainable dietary transition in the region.

**Systematic review registration:**

https://osf.io/d7j4m/

## 1 Introduction

South Asia, home to nearly 2 billion people, is currently facing a dual health burden: the persistence of malnutrition alongside the step rise of obesity, type 2 diabetes (T2D) and cardiovascular diseases (CVDs) (1–5). This evolving scenario is closely linked to rapid urbanization, socio-economic transitions, and the growing consumption of processed and ultra-processed foods (6–8). Furthermore, genetic predisposition renders South Asians more vulnerable to insulin resistance and metabolic disorders, with disease onset occurring at lower BMI levels compared to other ethnic groups (9, 10).

The Mediterranean diet (MD) is internationally recognized for its protective role against CVDs and its beneficial effects on glycemic control and overall metabolic health (11, 12). Its emphasis on whole grains, legumes, nuts, healthy fats and moderate amounts of fish and dairy has made it a global reference point (13, 14). Yet translating this pattern outside the Mediterranean basin poses challenges: cultural, culinary, environmental, and agricultural differences limit the feasibility and sustainability of directly adopting the MD in distant regions (15, 16). Several initiatives have attempted to address these challenges. The “Planeterranean” initiative, developed under the auspices of UNESCO, has proposed continental food pyramids, including one for Asia, by identifying regionally available foods with nutritional profiles comparable to those of the MD (17–19). While this provides a valuable global framework, its continental scope does not capture the marked heterogeneity that distinguishes South Asia from East and Southeast Asia in terms of staple grains, fat sources, religious dietary rules, and access to animal protein. Perspectives on heritage diets have similarly broadened the debate by highlighting the health-promoting potential of traditional dietary models from Africa, Latin America, and Asia (20), but they remain primarily descriptive rather than operational. Other contributions have emphasized the importance of cultural adaptation to ensure feasibility and sustainability in diverse settings (21, 22), yet concrete applications for South Asia are still lacking. Collectively, these approaches underscore the need to move beyond the Mediterranean basin, but also reveal a gap: the absence of region-specific, barrier-aware frameworks. The present study addresses this gap by combining dietary intake data with cultural, economic, and environmental determinants to design tailored food pyramids and substitution strategies directly applicable to South Asia.

Traditional South Asian diets already share several plant-based food features with the MD, including the frequent use of pulses and legumes, nuts, seeds, spices, and herbal infusions and moderate intake of animal foods, mainly as dairy. At the same time, they show systematic shortcomings: a predominance of refined grains, insufficient consumption of vegetables and fruits, low dietary diversity, and widespread use of unhealthy cooking fats, often fried or rich in n-6 fatty acids (23–27). These factors contribute to micronutrient deficiencies, poor antioxidant intake, and adverse metabolic outcomes, which are further exacerbated by the rapid spread of processed and ultra-processed foods (2, 6, 8, 28). While preliminary studies have suggested that a Mediterranean-style adaptation rooted in South Asian food cultures could provide substantial benefits, the feasibility, sustainability, and acceptability of such an approach on the whole population remain underexplored (29–32).

Against this background, the present study seeks to move from conceptual frameworks to operational tools. We applied a mixed-methods review to (i) synthesize available dietary intake data and contextual barriers across South Asia, and (ii) develop a Mediterranean-based, South Asian–specific food pyramid, with separate versions for vegetarian and non-vegetarian populations. By explicitly integrating nutritional, cultural, economic, and environmental dimensions, our framework aims to provide a practical basis for dietary guidance in the region, complementing global initiatives with context-specific recommendations that are culturally acceptable and nutritionally sound.

## 2 Methods

Due to the particular rationale described, this study employed a dual-method approach, combining a systematic review and a scoping review to comprehensively evaluate the feasibility of adapting the MD to South Asia. The systematic review was conducted to assess food and nutrient intakes, while the scoping review explored traditional foods, recipes, and key barriers affecting dietary adaptation. The protocol was preregistered on Open science Framework (OSF) prior to data extraction (33). Given the mixed-methods design (systematic + scoping), PROSPERO registration was not applicable. We followed PRISMA-2020 and PRISMA-ScR for the respective components (34, 35).

### 2.1 Configuration of a working group

We formed a working group consisting of three clinical nutrition experts (one methodological expert and two clinical practitioners), supported by a team of South Asian university fellows from the University of Eastern Piedmont with training in nutrition, who served as consultants for cultural validation of findings.

### 2.2 Conceptual framework

The study was guided by the following primary research question:

How can the principles of the MD be effectively transferred to the South Asian region?

Given the complexity of this issue, we formulated two core sub-questions, further divided into specific investigative aspects:

- What is the South Asian Diet?

- What are the registered food and nutrient intakes in the region?*(Systematic review of peer-reviewed literature).*
- What are the most common foods and recipes in South Asia?*(Scoping review, incorporating both peer-reviewed and non peer-reviewed sources).*

Answering these questions allowed us to identify the key differences between the Mediterranean Diet and South Asian dietary patterns, integrating both systematic nutritional data and cultural dietary perspectives. To establish a robust comparative framework, we defined the MD characteristics based on the Mediterranean Diet Foundation Expert Group guidelines. Reference intakes and portion recommendations were aligned with those from the PREDIMED study and EAT Lancet, ensuring that proposed adaptations maintained scientific validity and dietry integrity (13, 14, 36, 37).

- Why is the South Asian diet different from the MD?*(Scoping review on barriers to dietary adaptation).*

- What are the socio-economic barriers influencing food consumption in South Asia?
- What are the cultural barriers influencing food consumption in South Asia?
- What are the environmental barriers influencing food consumption in South Asia?

**Figure 1:**
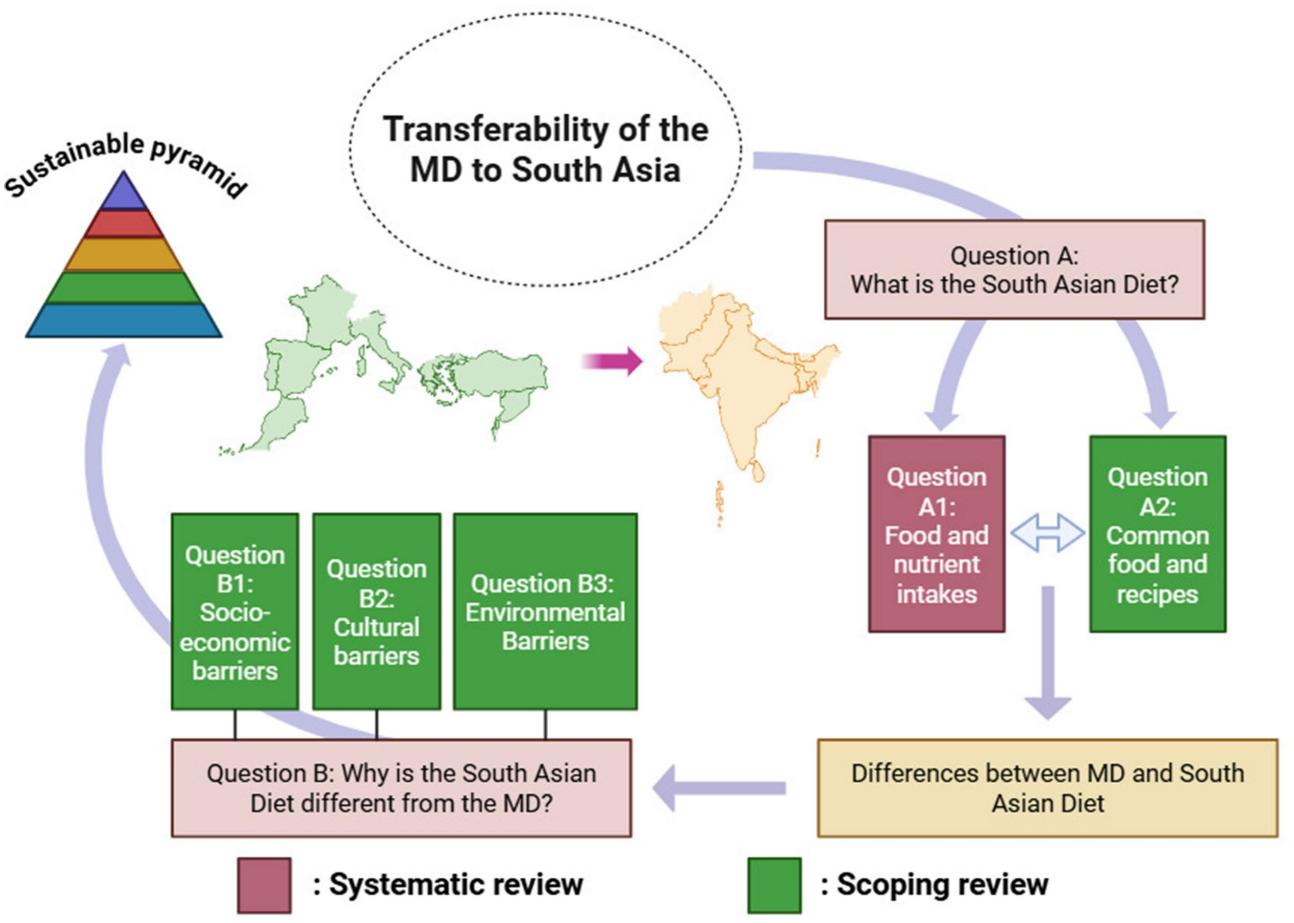
Conceptual diagram flow for the methodology of this review.

To further contextualize these dietary differences, we conducted a second scoping review (b-1 to b-3) aimed at identifying the key barriers preventing a Mediterranean-aligned dietary shift in South Asia. This review assessed socio-economic constraints, cultural food practices, and environmental factors influencing food choices. A graphical visualization of the conceptual framework is shown in Figure 1.

### 2.3 Inclusion and exclusion criteria

We included cross-sectional studies on human populations (adults aged over 18 or not age-restricted) that reported quantitative dietary intake data—including food consumption, food frequency, macronutrient or micronutrient intakes, or nutrient deficiencies—from at least one South Asian country (India, Pakistan, Bhutan, Bangladesh, Sri Lanka, Maldives, Afghanistan, or Nepal). We excluded studies involving children, adolescents, pregnant women, individuals with specific diseases, studies with fewer than 100 participants, and those conducted in single towns or small locations, as they were not sufficiently representative of the South Asian population.

For the scoping review, we included articles retrieved from the systematic search and additional sources identified through manual searches, cross-referencing, and grey literature. Given that all studies provided at least some information regarding commonly consumed food, studies had to clearly identify at least one other aspect linked to our scoping methodology (cultural, socio-economical, or environmental barriers against changes toward a healthier Mediterranean dietary pattern, or insightful aspects of South Asian typical food and recipes). Where one or another aspect was strongly suspected but unclear from the abstract, we accessed the full text to make a final decision on inclusion. The investigation of traditional cuisine and local food elements was enriched through the use of generic websites together with consultation with South Asian university students from our network.

### 2.4 Literature search

For the systematic review, a search was conducted in PubMed (including MEDLINE and PMC) and Semantic Scholar, covering publications from January 2010 to December 2023. The search strategy, detailed in Supplementary File 1, was designed using Boolean operators to retrieve relevant dietary studies while excluding research on non-representative populations.

The scoping review followed a more flexible search strategy to capture dietary patterns, food culture, and barriers to adaptation. It included searches in academic databases, grey literature reports, government policy documents, and qualitative studies focusing on traditional food practices and socio-cultural influences.

In addition to peer-reviewed sources, information on commonly consumed foods, traditional recipes, and preparation techniques was retrieved from digital repositories and grey literature (e.g., recipe databases, food blogs, historical records). All content was manually reviewed and validated by the research team, and cross-checked with South Asian university students to ensure cultural accuracy and relevance.

### 2.5 Study selection

In the initial phase, three authors (DS, FC, and AC) screened titles and abstracts to exclude non-relevant studies. The full texts of the remaining records were then retrieved and assessed for eligibility. To ensure consistency, the author group held several meetings to align on the conceptual framework, refine inclusion and exclusion criteria, and discuss problematic cases. For the final eligibility stage, two reviewers (DS and AC) independently evaluated the studies, with discrepancies resolved through discussion and, when necessary, consensus with a third reviewer (FP).

### 2.6 Data charting and synthesis

For the systematic review, extracted data were organized into structured tables summarizing study characteristics, population demographics, reported food and nutrient intakes, and key findings. The critical appraisal of included studies was conducted using the Newcastle-Ottawa Scale (NOS), modified for cross-sectional studies, following the methodology outlined by Herzog et al. (38).

The scoping review was structured to map findings across two primary areas:

1. Traditional foods and culinary practices, which were analyzed in a table summarizing common food items, their nutritional profiles, recipes, preparation methods, and potential modifications for better adherence to MD principles.
2. Dietary barriers, categorized by socio-economic, cultural, and environmental constraints. Key findings from each included study were synthesized into a barrier mapping table, highlighting regional variations in dietary challenges.

Based on the synthesized evidence from both the systematic and scoping reviews, we developed a culturally adapted MD pyramid for South Asia. This pyramid incorporated local foods and traditional dietary practices, ensuring that the proposed model was both nutritionally adequate and culturally sustainable.

### 2.7 Quality assessment

For the systematic review, the studies’ quality assessment was conducted using an adapted form of the NOS for cross-sectional studies, rearranged to fulfill the requirements and respect the crucial aspects linked to the review. The rearrangement was conducted starting from original NOS following a similar methodology as for the article from Herzog et al. (38). The scale comprises seven questions, yielding a total score of 9 points, and assesses the quality of the following aspects: (i) selection; (ii) sample size; (iii) non-respondents; (iv) control of confounders (2 points); (v) assessment of outcomes (2 points); (vi) statistical test; (vii) ascertainment of the outcome measurement (Supplementary File 2). Quality assessment was performed by two researchers (DS, AC) and disagreements were resolved by consensus.

For the scoping review, following PRISMA-ScR guidelines, a formal quality assessment was not conducted, as the goal was to explore the scope of available research rather than evaluate study rigor. However, credibility checks were performed to exclude sources lacking clear authorship, methodological transparency, or relevance.

### 2.8 Dietary pyramid

The development of the adapted MD pyramid for South Asia followed an evidence-based and iterative process, integrating quantitative findings from the systematic review and qualitative insights from the scoping review. The objective was to outline a regionally tailored dietary model capable of preserving the core health-promoting principles of the MD while remaining sensitive to the economic, cultural and environmental realities of South Asia.

At the methodological level, the process was structured around three predefined steps: (i) identifying areas of divergence between MD recommendations and South Asian dietary patterns; (ii) formulating adaptation rules grounded in nutritional requirements, feasibility constraints, and cultural relevance; and (iii) determining *a priori* whether separate vegetarian and non-vegetarian versions were required, given the central role of religiously structured dietary habits in the region.

To operationalize the iterative nature of the process, each proposed adaptation was repeatedly re-evaluated across the evidentiary layers. Nutritional indications derived from the systematic review were cross-checked against the feasibility constraints emerging from the scoping review. Whenever discrepancies arose, the adaptation rule was revised and reassessed until the recommendation satisfied all dimensions simultaneously.

## 3 Results

### 3.1 Systematic review

**Figure 2:**
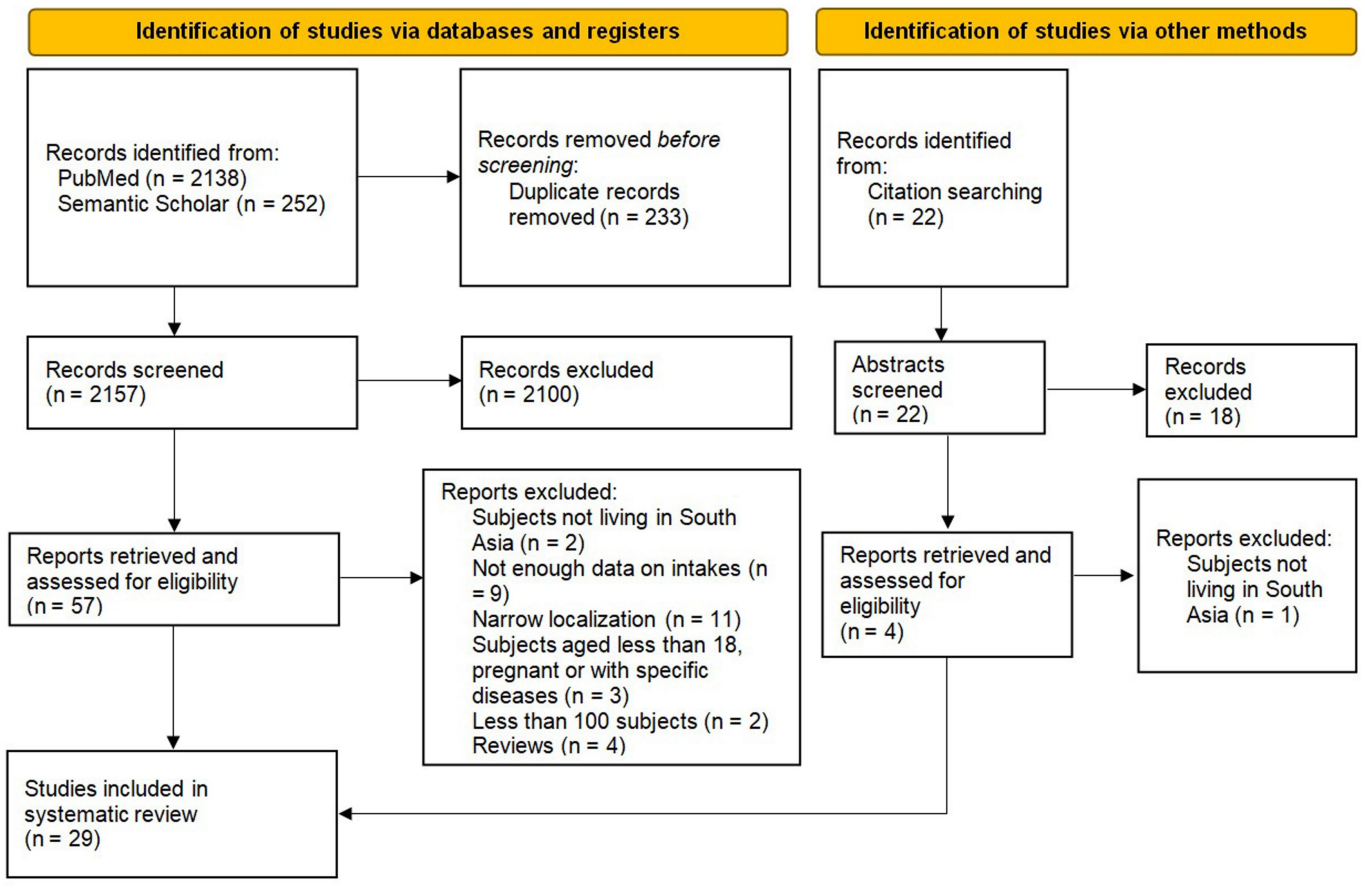
Flow chart of the systematic review.

The electronic search through PubMed identified 2,138 articles for review and an additional 252 were retrieved through Semantic Scholar. After screening the titles and abstracts, we excluded 2,100 articles due to deviations from our inclusion criteria or due to record duplication. A total of 57 full texts were examined, and after adding four more reports identified through citation searching, we finally included 29 cross-sectional studies for the systematic review (Figure 2). The overall quality of studies ranged from 3 to 9 points (mean = 6.24; median = 7). Initial inter-rater agreement between the two reviewers was 86%, with all discrepancies resolved through consensus. The distribution of quality scores showed recurrent weaknesses in the high prevalence of non-responders and the lack of comparability between responders and non-responders (selection bias) (Supplementary File 3). Other quality discriminants were the moderate prevalence of 24-h dietary recalls rather than semiquantitative food frequency questionnaires and the use of non validated methods or estimation methods for the assessment of nutrient intakes. Most of the included studies were conducted in India (15), with less information coming from Bangladesh (5), Sri Lanka (4), Nepal (3) and Pakistan (2). No studies were included from Bhutan, Afghanistan and Maldives.

#### 3.1.1 Average energy and nutrient intakes in South Asia

Intakes varied heavily among different country and no data were available from Nepal, Bhutan, Afghanistan and Maldives. Furthermore, reporting intakes measure units were sometime discordant between each other (i.e., for macronutrients we recorded g/day or % of total energy intake). In Table 1 we resumed averages intakes for each country and more details are presented in Supplementary Table 4.

**Table 1:** Average calculated energy and nutrient intakes for each South Asian country.

	India	Sri Lanka	Bangladesh	Pakistan
Energy (kcal/day)	1,555–3,174	1,439–2,230	2,468–3,204	2,227
Carbohydrates (g/day)	364–417	270–306	512	316–355
Protein (g/day)	46–78	23–50	60	55–71
Fat (g/day)	27–76	25–58	34	40–66
Fibre (g/day)	13–35	18–22	/	/
Sodium (mg/day)	2,863–4,100	2,729–2,890	/	/
Calcium (mg/day)	808–981	220	677	580
Iron (mg/day)	12–25	7	20	28
Zinc (mg/day)	7–24	1–7	/	11
Vitamin A (μg/day)	1,063–1317^a^	180–652	/	457
Vitamin C (mg/day)	68	24	/	/
Vitamin B12 (μg/day)	0.73–2.2	0.86	/	/
Folate (μg/day)	163–366	49	/	/

Single values without a range derive from a single study, while the first and second numbers of a range represent the lowest and highest average values reported across multiple studies, respectively. For more details, see Supplementary Table 4. ^a^Intake of *β*-carotene.

#### 3.1.2 Average food intakes in South Asia

Included studies that calculated the average food intakes among South Asian countries used different ways of reporting data (g/day; portions/week; % of participants achieving daily consumption; relative kcal/day; % of participants achieving specific frequencies of consumption; prevalence of adequate consumption) (Supplementary File 4). Furthermore, there was also a lack of standardization when referring to specific food groups.

Overall dietary patterns in South Asia are characterized by a very high carbohydrate intake, mainly attributable to refined grains (rice or wheat, depending on the regions), especially in Bangladesh, and low limited dietary diversity among food groups, with a low to very low intake of vegetables, fruits, legumes, nuts, eggs, fish and meat. Non refined grains intake was also found, when data were available, to be negligible in some studies (39, 40), with slightly higher intakes around 50 g/day reported in India (41, 42), compared to the average refined grain intake mostly ranging between 200 g/day and 450 g/day (39, 40, 42–47). Data on dairy and fish intakes varied widely, with coastal regions and Bangladesh including almost satisfying quantities of fish in their diets (43, 44, 46, 48, 49), while in India and Pakistan dairies products were more consumed (27, 39, 41, 42, 45–47, 50). Meat consumption did not exceed 50 g/day across all states (27, 39, 41–45, 47–50), a part for one study in West India that reported a mean intake of 82 g/day (46). Legumes and pulses consumption was generally low, generally ranging from 10 to 20 g/day (39, 41, 43, 44, 47, 48, 50) to 50 g/day (42, 45, 46). Fruit and vegetables intake was investigated by several studies, with different average intakes being displayed: in most studies the cumulative intake of fruit and vegetables was recorded to be between 100 g and 350 g/day (39, 41–44, 46, 47, 50, 51). Two studies in Nepal and Sri Lanka that reported high intakes of fruit and vegetables in the form of servings/week, without specifying the actual portions (40, 49). Spices intake (mainly from chilies) was reported to be non-negligible in some studies, with an estimated average intake between 30 g/day and 60 g/day (43, 47, 50). Cooking fats and oils intake ranged from 30 to 40 g/day in most studies (6, 41, 42, 45, 49, 50), with other report indicating a lower consumption, around 15-20 g/day, in rural India and Bangladesh (39, 43, 44). Added sugar ranged between 5 and 50 g/day (39, 42, 44, 47). Sweets intake was recorded in one study between 10 and 40 g/day (43).

### 3.2 Scoping review

**Figure 3:**
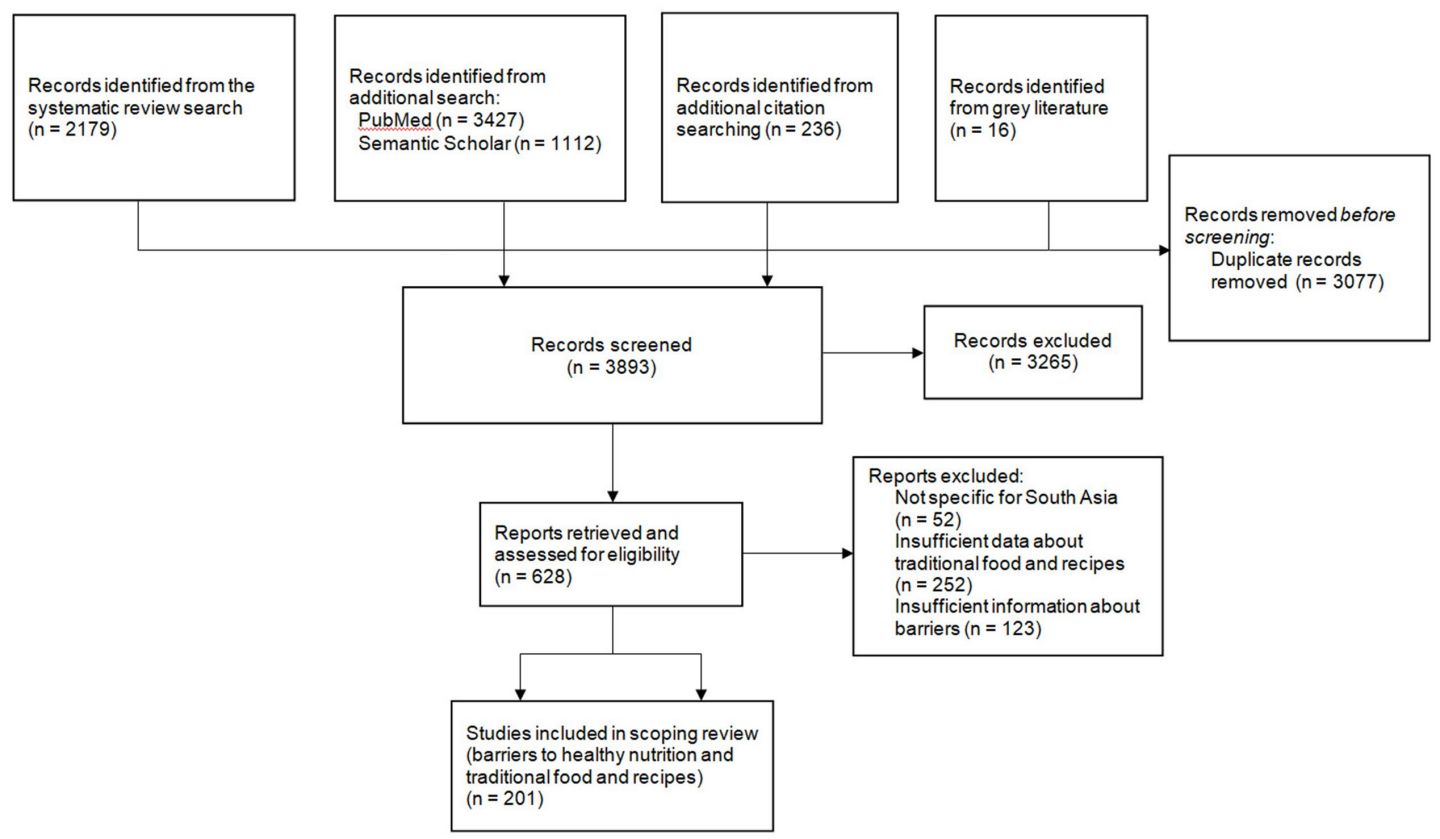
Flow chart of the scoping review.

We identified a total of 6,970 records from the systematic review, additional database searches, citation searching, and grey literature (Figure 3). After removing 3,077 duplicates, we screened 3,893 titles and abstracts, excluding 3,265 for lacking relevant scoping themes. We examined the full texts of the remaining 628 articles, ultimately including 201 in the scoping review. Of these, 159 articles pertained to the analysis of barriers, and 42 focused on the assessment of traditional food and recipes. The latter group was supplemented by non-peer-reviewed sources to enrich our understanding of the regional dietary practices.

#### 3.2.1 Traditional foods and recipes in South Asia

Permission must be obtained for use of copyrighted material from other sources (including the web). Please note that it is compulsory to follow figure instructions.

#### 3.2.2 Comparison with Mediterranean diet recommended intakes

**Figure 4:**
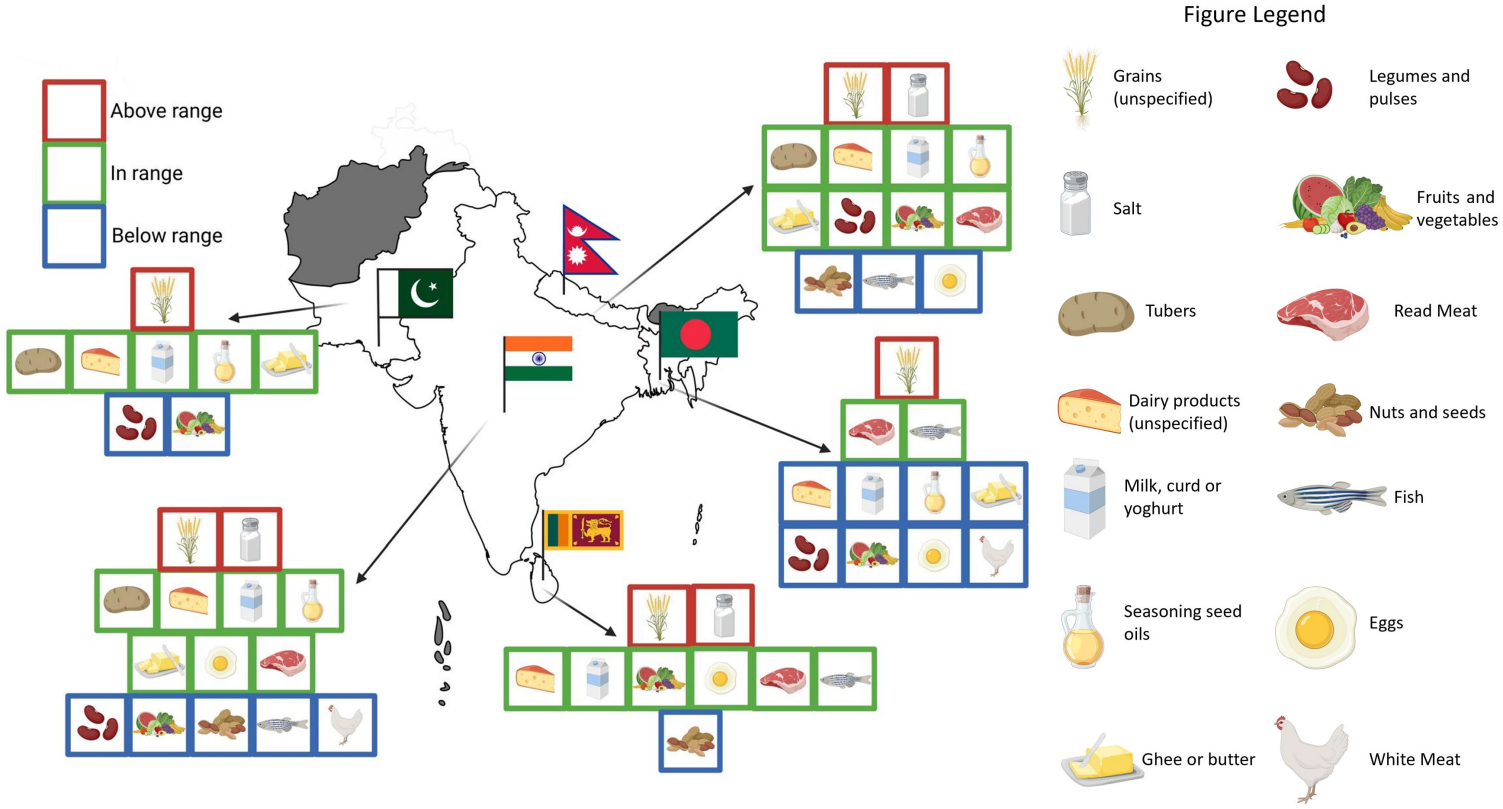
South Asian food patterns and intakes compared to EAT-Lancet reference diet and MEDAS. Graphical distribution. Only food categories with sufficient data from the systematic review are represented in the figure. The placement of each icon was determined based on a semi-quantitative average of food intakes in each country, compared to the average recommended intakes by the EAT-Lancet reference diet and the MEDAS score. Values above the range indicate >30% of the recommended intake, values within the range denote adequate adherence, and values below the range indicate <30% of the recommended intake. Created with BioRender.com.

Food consumption patterns in South Asia diverged significantly from the recommendations outlined by the EAT-Lancet Commission and the latest Mediterranean diet reference pyramids and profiles (Table 2). While dietary trends varied across countries, a consistent pattern of excessive consumption of refined grains, contrary to guidelines favoring whole grains, and high salt intake emerged throughout the region. Legumes and nuts, where data were available, were consistently below the recommended thresholds. Fish consumption was markedly low across most countries, with the exception of Sri Lanka and Bangladesh, where intake was adequate. Fruit and vegetable intake frequently fell below recommended levels in most countries, except for Nepal and Sri Lanka, where consumption was comparatively higher (Figure 4).

**Table 2:** Current international and Mediterranean guidelines for a healthy diet.

	EAT lancet reference diet (37)(g/day)	Mediterranean diet (MEDAS/PREDIMED) (137) (g/day)	Mediterranean diet (Pyramid 2011) (14) (servings/day)	Mediterranean diet (Pyramid 2020) (138) (servings/day)
Grains (preferably whole)	232	Not specified	2–4	2–4
Starchy tubers	50	Not specified	≤ 0.28	2–4 (together with grains)
Vegetables	300	> 250	≥ 4	≥4
Fruits	200	> 375	2–4	2–4
Dairy	250	400	2	2
Red Meat	14	< 150	<0.28	≤0.28
White meat	29	42.9	0.28	0.28
Eggs	13	Not specified	0.28–0.57	0.28–0.57
Fish	28	> 42.0/64.3	≥ 0.28	≥ 0.28
Legumes/pulses	75 (dried)	> 17.1 (dried)	≥ 0.28	1
Nuts	50	> 10.7	1–2	1–2
Unsaturated oils	40	> 40 (virgin olive oil)	Not specified (virgin olive oil)	Not specified (virgin olive oil)
Dairy fat and other saturated fat	11.8	< 12	Not specified	Not specified
Sugar	31	Not specified	Not specified	Not specified
Sweets	Not specified	Less than 2 portions of commercial sweets/week	≤ 0.28	≤0.43
Wine	/	> 100 (if consumed)	Not specified	Not specified

#### 3.2.3 Barriers towards a healthier diet in South Asia

For each of the three main barriers, we classified articles based on which topics were being discussed as potential barriers, individuating two main topics for each of the barriers: “Income levels/cost of nutritious food” and “urbanization/modernization” for the socio-economic barrier, “cultural/religious tradition” and “lack of awareness/education” for the cultural barrier, and “climate change” and “pollution/contamination” for the environmental barrier. A synthesis of our search, divided by country, is illustrated in Table 3 and an analytical classification by topic and an evaluation of key findings for each article is available in Supplementary Table 6.

**Table 3:** Synthesis of articles divided by topics as barriers towards a healthier diet in South Asia.

	Socio-economic		Cultural		Environmental		Total articles (*N* = 159)
	Income levels/cost of nutritious food	Urbanization/ modernization	Cultural/ religious traditions	Lack of awareness/ Education	Climate change	Pollution/ contamination	
Afghanistan	3	0	2	4	1	1	4
Bangladesh	22	7	14	23	10	9	25
Bhutan	0	0	0	0	0	0	0
India	42	46	46	64	9	11	77
Maldives	0	0	0	0	0	0	0
Nepal	9	1	8	12	6	1	12
Pakistan	14	2	6	18	6	13	19
Sri Lanka	5	2	5	8	1	2	8
South Asia	10	7	10	13	5	3	14
Total	105	65	91	142	38	40	

##### 3.2.3.1 Economic barriers

###### Income levels and cost of nutritious food

Low income is a key barrier to healthy eating in South Asia. Nutritious foods such as fruits, vegetables, pulses, and whole grains are often unaffordable for poorer populations, leading to a reliance on calorie-dense but nutrient-poor staples (52–54). Meanwhile, urban and higher-income groups are seeing a rise in processed food consumption, contributing to malnutrition and NCDs (6, 8, 23, 52, 55). Foods rich in essential nutrients, such as animal-based sources of iron and zinc, also remain economically inaccessible for many families (56).

###### Urbanization and modernization

Urbanization and changing lifestyles are rapidly reshaping food environments and habits. In Northern India, for instance, rising urbanization has coincided with greater availability and consumption of ultra-processed foods—such as ready-to-eat meals, sweet snacks, and fast food—often due to limited time for meal preparation (7, 8, 57). Meanwhile, healthier snack options like milk-based beverages are in decline (6). Nevertheless, also due to the mean difference in income, urban population generally has a higher dietary variety compared to rural populations (58, 59), but not in Sri Lanka (60). Historically, urban populations have also shown higher rates of overweight and obesity due to more sedentary lifestyles, though recent studies show this pattern may be shifting (61–63).

##### 3.2.3.2 Cultural barriers

###### Cultural and religious traditions

In South Asia, religious and cultural norms are deeply embedded in food choices, sometimes restricting dietary diversity. Hindu vegetarianism, especially in its stricter forms that exclude eggs, may lead to deficiencies in protein, vitamin B12, iron, and zinc (64–67). Similarly, Ayurvedic dietary principles promote food combinations and preparation methods rooted in tradition. While these offer a culturally meaningful approach to wellness, they can also impose rigid restrictions that hinder modern nutritional flexibility (68, 69).

Cultural expectations also govern intra-household food distribution. Men, often regarded as the main providers, typically receive larger and more nutritious portions, whereas women—especially in rural settings—are responsible for food acquisition, preparation, and childcare, yet frequently eat last and least. This dynamic raises the risk of malnutrition among women and children (55, 70–73).

Food taboos, particularly surrounding specific meats or combinations deemed impure or harmful, further reduce the intake of nutrient-dense foods (68, 69, 74, 75).

Even in urban areas where food availability has expanded, preferences for traditional staples like rice and wheat endure, often resulting in hybrid diets that maintain caloric sufficiency but lack balance and variety (8, 41, 43, 76).

###### Lack of nutritional awareness and education

A widespread lack of nutrition education exacerbates poor dietary habits. Limited public health campaigns and insufficient integration of nutrition into school curricula contribute to widespread unawareness about affordable, locally available nutrient-rich foods like leafy greens, pulses, and fortified staples (23, 77, 78).

This problem is particularly acute in rural areas, where knowledge gaps are compounded by infrastructural barriers and traditional gender roles. Women, who typically manage household nutrition, often lack access to education, perpetuating suboptimal feeding practices across generations (23, 76, 79–82).

Public awareness about food safety is also lacking. Contamination from heavy metals, pesticides, and unsafe water remains a serious risk, especially in staples like rice. However, few are aware of safe handling practices or the long-term health consequences of exposure to such contaminants (83–86).

##### 3.2.3.3 Environmental barriers

###### Climate change

South Asia is highly vulnerable to climate change, which threatens both food security and nutrition. Rising temperatures, erratic rainfall, and extreme weather events increasingly disrupt staple crop production—especially rice and wheat—in regions like the Indo-Gangetic plains. Groundwater depletion, salinization, and flooding in coastal areas like Bangladesh and Sri Lanka exacerbate these effects, reducing agricultural productivity and damaging livelihoods (87–90).

Warm and humid conditions also promote mycotoxin-producing fungi, resulting in aflatoxin contamination of maize, rice, and groundnuts, with major public health and economic consequences (91–93). Smallholder farmers are especially affected. Lacking financial resources and access to resilient technologies, they struggle to adapt through strategies such as crop diversification or improved irrigation (55, 74, 89).

###### Chemical pollution and biological contamination

Agricultural safety is further compromised by environmental contamination. Arsenic in groundwater, especially in Bangladesh, India, and Nepal, has been detected at hazardous levels in rice crops, leading to chronic health conditions like cancer and impaired nutrient metabolism (46, 85, 94, 95). Industrial adulteration of turmeric with lead and chromium presents another significant concern (83).

As previously highlighted, South Asia’s hot and humid climate fosters the growth of mycotoxin-producing fungi, with aflatoxins posing serious health threats and economic losses. Contamination levels in rice, maize, and groundnuts often surpass international safety standards, linked to improper storage and lack of regulation (91–93).

The excessive use of pesticides—often unregulated—has led to chemical residues in fruits and vegetables, particularly when irrigated with untreated wastewater. These residues are associated with endocrine disruption, developmental delays, and carcinogenic risk (96, 97). Marine ecosystems near industrial coasts are also polluted, with polychlorinated biphenyls (PCBs) accumulating in fish and posing serious health threats (98–100).

Poor hygiene and the use of polluted water in irrigation lead to contamination of fresh produce with pathogens like Salmonella and *E. coli*. These microbial hazards, coupled with the emergence of antibiotic-resistant strains, contribute to a growing burden of foodborne diseases across the region (101, 102).

### 3.3 Integrated evidence

The iterative framework resulting from the integration of the systematic and scoping reviews produced two operational outputs: (i) the adapted dietary pyramids and (ii) a set of complementary recommendations.

**Figure 5:**
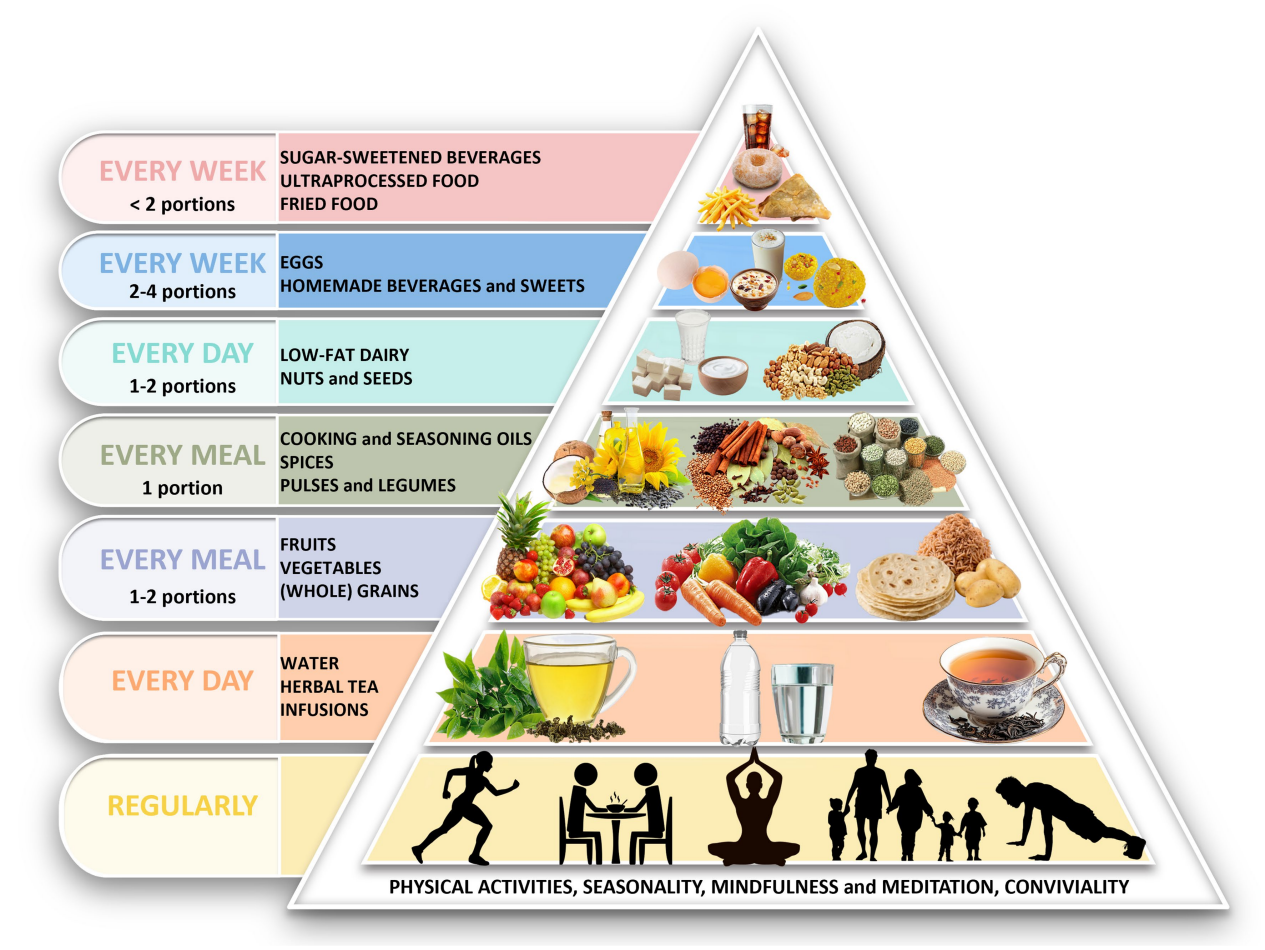
Adaptation of Mediterranean diet pyramid for South Asia (vegetarians). Pyramid illustrates the recommended food groups and their relative proportions per meal, day, or week within a traditional South Asian vegetarian dietary pattern. The base includes physical activity, mindfulness, and conviviality. Daily intake includes water/herbal infusions, fruits, vegetables, whole grains and tubers, vegetable oils, legumes and pulses, spices, nuts and seeds, and low-fat dairy. Eggs and homemade sweets are suggested in moderation on a weekly basis, while sugar-sweetened beverages, fried foods, and ultra-processed products should be limited. The color scheme in pyramid reflects intake frequency: daily (yellow, orange, light purple, grey, light blue), weekly (blue), and occasional (pink). Pyramid edited with Adobe Photoshop cc 2025. The description of the food groups is provided in Supplementary File 5.

**Figure 6:**
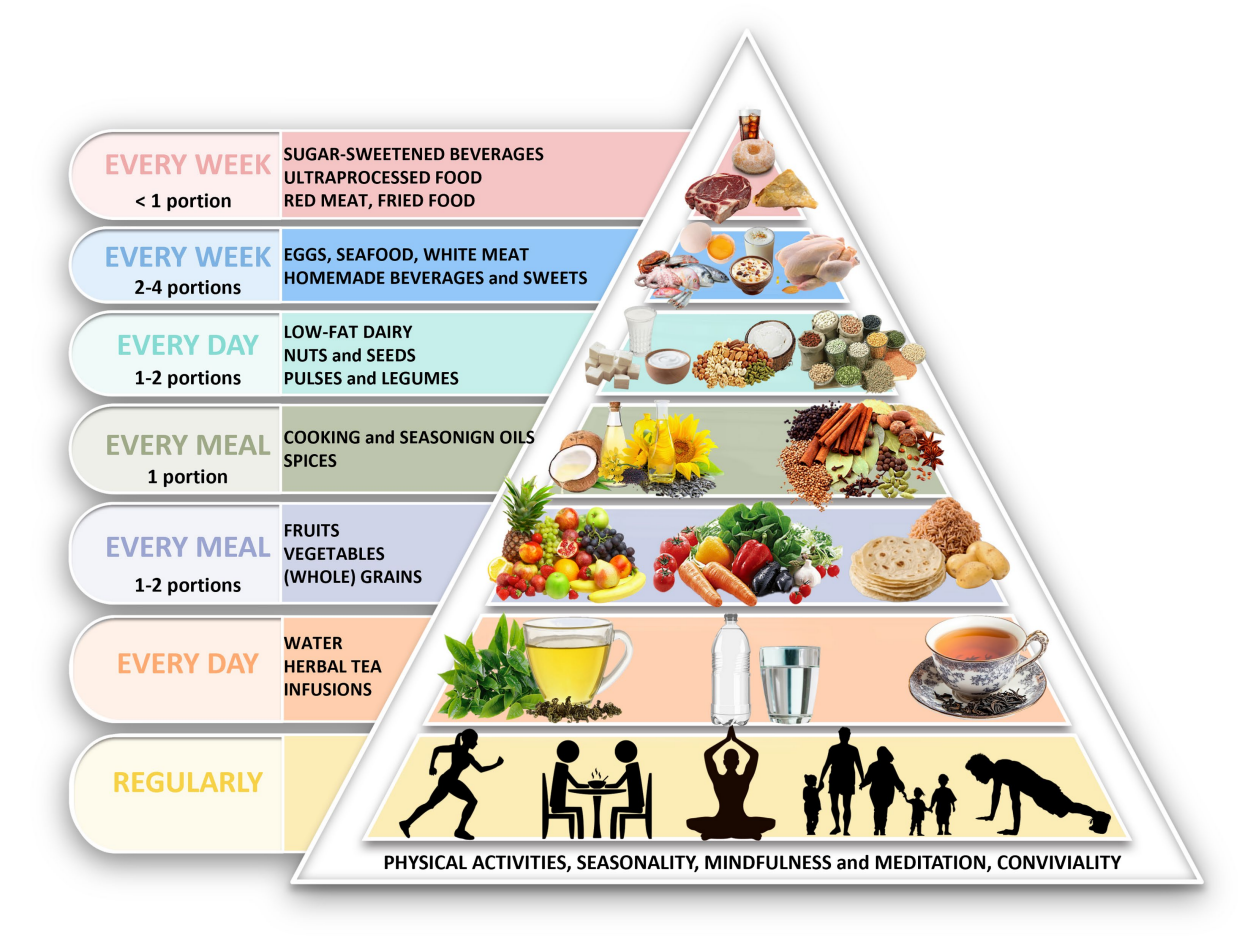
Adaptation of Mediterranean diet pyramid for South Asia (non-vegetarians). Pyramid illustrates the recommended food groups and their relative proportions per meal, day, or week within a traditional South Asian non-vegetarian dietary pattern. The base includes physical activity, mindfulness, and conviviality. Daily intake includes water/herbal infusions, fruits, vegetables, whole grains and tubers, vegetable oils, legumes and pulses, spices, nuts and seeds, and low-fat dairy. Eggs, fish, seafood, white meat and homemade sweets are suggested in moderation on a weekly basis, while red meat. Sugar-sweetened beverages, fried foods, and ultra-processed products should be limited. The color scheme in pyramid reflects intake frequency: daily (yellow, orange, light purple, grey, light blue), weekly (blue), and occasional (pink). Pyramid edited with Adobe Photoshop cc 2025. The description of the food groups is provided in Supplementary File 5.

- The pyramids (Figures 5, 6) were structured according to the predefined adaptation rules and reorganized using locally available foods. Key outputs were:

- Cereals and tubers placed at the same foundational level.
- Legumes and pulses designated as daily protein sources.
- Dairy retained as a primary protein source for vegetarians.
- Fish, eggs, and white meat included as weekly options in non-vegetarian patterns.
- Fat sources reorganized to improve the n-6/n-3 profile using culturally common oils.
- Nuts and seeds recommended regularly but in moderated amounts due to cost.
- Red meat, ultra-processed foods, fried snacks, and sugar-sweetened beverages positioned at the top as items to minimize.

Both pyramids also incorporate foundational lifestyle elements (seasonality, conviviality, physical activity, outdoor time, rest, mindfulness, safe water access). No specific guidance on alcoholic beverages was included.

- Additional outputs derived from the integrated evidence included actions that need to be taken by policymakers:

- Expanding nutrition-education initiatives through schools, community programs and women-focused training to improve awareness of affordable, nutrient-dense foods.
- Supporting affordability-oriented actions such as reducing the cost of legumes, pulses, vegetables and fruits to facilitate substitution of refined grains.
- Strengthening food-safety controls by improving regulation of pesticide use, irrigation water quality, and storage conditions to limit exposure to heavy metals, aflatoxins and microbial contamination.
- Promoting climate-resilient agricultural practices to ensure long-term availability of diverse, nutrient-rich foods despite environmental vulnerabilities.

A detailed breakdown of recommended frequencies and food categories is available in Supplementary File 7, while the iterative analytical process leading to both the pyramids and the complementary recommendations is summarized in Supplementary File 8.

## 4 Discussion

This mixed-method review demonstrates that a direct transplantation of the MD is unfeasible in South Asia, and that culturally adapted frameworks are necessary.

From the analysis of dietary and nutrient intakes, South Asian diets appear calorically adequate but are heavily reliant on refined carbohydrates, low in fiber and micronutrients, and characterized by imbalances in fat composition and insufficient high-quality protein sources, particularly among vegetarians. These imbalances are not merely a matter of individual choice but are strongly shaped by structural barriers. Economic constraints drive the reliance on refined staples; cultural traditions underpin frying practices and the centrality of sweets; and environmental challenges such as climate variability and food contamination further restrict access to nutrient-dense foods. Limited awareness of dietary inadequacies compounds these issues, reducing the feasibility of adopting healthier patterns.

Our work is particularly crucial since the region is highlighting increasingly alarming epidemiological trends, with a high and growing burden of T2D, CVDs, and other metabolic disorders. The PURE study has identified CVD as the leading cause of death in South Asia with rural areas showing especially high prevalence and mortality due to lower dietary diversity and limited healthcare access (103). South Asians also experience cardiovascular events 5–10 years earlier than other populations, often with worse clinical outcomes, partly reflecting under diagnosis and inadequate access to preventive care (104). Findings from the Global Burden of Disease (GBD) study confirm that ischemic heart disease, stroke, and chronic obstructive pulmonary disease are now among the main causes of mortality, with high blood pressure, hyperglycemia, and poor diet as the predominant risk factors (105). Projections further suggest that CVD-related mortality in South Asia will nearly double by 2050, in stark contrast to the declining cardiovascular mortality seen in many other regions (106).

### 4.1 Sustainability of protein sources

Our findings align with and expand upon existing dietary frameworks for South Asian populations, particularly in relation to official guidelines, intervention studies, and Mediterranean Diet (MD) adaptations. Similarly to our recommendations, the Indian (ICMR-NIN) (107, 108) and Pakistani (PDGN) (109) dietary guidelines advocate for a predominantly plant-based diet, emphasizing whole grains, legumes, fruits, and vegetables, with dairy as the primary complementary protein source. These guidelines also highlight the role of spices, nuts, and seeds as key nutrient sources, particularly in relation to n-3 polyunsaturated fat intake. However, our review identifies a significant gap between these recommendations and actual dietary intake, with persistent deficiencies in fiber, micronutrients, and high-quality proteins. Furthermore, the Indian protein intake recommendation (RDA: 54 g/day for men, 46 g/day for women) appears overly optimistic, as it does not fully account for the lower protein quality of pulses. Given the suboptimal digestibility and amino acid profile of plant-based proteins, we argue that a more appropriate target would be at least 1 g/kg/day (110). Specifically, we propose an increase in legume and pulse consumption beyond the ICMR’s 120 g/day current recommendation for combined pulses and flesh foods, as well as the PDGN’s 56-84 g/day for adults (107–109).

The role of dairy intake remains particularly controversial when compared to the EAT-Lancet recommendations: while the EAT-Lancet India model advocates for a plant-based diet with reduced dairy intake, our findings indicate that not only dairy intake was in range in most countries among reviewed studies, but also that dairy remains a critical protein source, particularly among Indian and Pakistani vegetarian population, due to low egg consumption, affordability, and the lack of widely available high-quality protein alternatives (24, 37, 56). Moreover, dairy in South Asia is frequently consumed in low-fat forms and, in many cases, presents a more sustainable alternative to meat. Given these factors, we claim that a strict reduction in dairy intake is unnecessary, provided that total added fat intake remains controlled. Instead, our recommendation is to maintain dairy intake among vegetarians, while simultaneously increasing legume and pulse consumption and reducing refined grains, a strategy that aligns better with both nutritional adequacy and long-term feasibility. However, a different approach is necessary for non-vegetarian populations and for Bangladesh, where the availability of dairy is lower than that of meat and fish. In these cases, dairy intake should be balanced with the consumption of flesh foods, ensuring a diversified and sufficient intake of high-quality protein sources within the diet.

### 4.2 Optimizing fat intake for South Asia

Understanding the appropriate balance of added fats is crucial for South Asia, particularly given the absence of olive oil, which serves as the primary fat source in the Mediterranean diet due to its health benefits, versatility, and widespread availability. In contrast, South Asian diets rely on a variety of oils with differing nutritional profiles, making it essential to optimize fat choices to address the region’s prevalent n-6/n-3 fatty acids imbalance, which is linked to multiple negative health outcomes (111–113).

To provide a structured approach to fat intake, both national and global dietary guidelines offer recommendations adapted to different nutritional priorities. The ICMR suggests 30 g/day of added fats for an adult man, emphasizing the need to incorporate n-3 polyunsaturated fatty acids (PUFA) through mustard, canola/rapeseed, and soybean oils, particularly when combined with n-6-dominant sources such as groundnut, sesame, rice bran, and sunflower oils (107, 108). Meanwhile, the EAT-Lancet framework proposes a broader sustainability-driven model, recommending 40 g/day of unsaturated fats, 7 g of saturated fat, and 5 g from animal sources (lard/tallow), while excluding dairy-based fats altogether (37). Given regional cooking habits—where oils are predominantly used for frying and high-temperature cooking—we propose an adjusted recommendation of 20–30 g/day, with careful selection of fat sources to maximize nutritional benefits while maintaining cultural acceptability. A previous Indian consensus for the prevention of hypertension and coronary artery disease had already suggested 25 g/day of fat intake, recommending a combination of mustard and soy oil, along with limited amounts of coconut oil or ghee (114). This model was later refined by Singh and colleagues, who experimented with a modified Mediterranean-inspired approach, incorporating walnuts or almonds alongside other typical Mediterranean diet elements, in what was called the Indo-Mediterranean diet. While this study was the first structured attempt to adapt the Mediterranean diet to South Asia using local foods, its design introduced substantial inconsistencies in the n-6/n-3 ratio. Specifically, mustard and soy oils were presented as interchangeable alternatives, despite having different fatty acid compositions, and the inclusion of walnuts and almonds—which also differ significantly in their n-6/n-3 ratios—added an additional nutritional bias to the intervention. Moreover, while this model demonstrated cardiovascular benefits, it did not assess the feasibility of large-scale implementation, an aspect that is central to our current approach (31).

Given these limitations, it is essential to reassess the role of commonly used fats in South Asia. Aside from vanaspati, which is widely recognized for its detrimental health effects due to its high trans fatty acid content (115), each fat source presents in fact both advantages and limitations. Mustard and rapeseed oils are rich in n-3 PUFA but also contain erucic acid, which may raise health concerns if consumed in excess (116, 117). Canola oil, while lower in erucic acid, is often refined, reducing its bioactive compounds (117); Soybean oil, despite providing some n-3 PUFA, is heavily refined and excessively high in n-6, making it unsuitable for improving the n-6/n-3 ratio. Similarly, sesame, groundnut, rice bran, and sunflower oils, though beneficial in moderation, are too rich in n-6 to serve as primary fat sources (118). Conversely, ghee is highly stable for frying and high-heat cooking, as it does not degrade at high temperatures. It also provides SCFA and MCT, particularly when combined with coconut oil, offering metabolic benefits. Additionally, being almost free of n-6, it may help correct the n-6/n-3 imbalance. However, its high saturated fat content requires careful consumption, particularly in populations at risk of cardiovascular disease (116, 119).

To mitigate the risks associated with an excessive reliance on any single fat source, a diverse and balanced approach is necessary, adapting fat choices to both local availability and n-3 intake. Since direct dietary sources of n-3 PUFA, such as fish (for non-vegetarians) and ALA-rich seeds (walnuts, flaxseeds, sabja seeds, and white/yellow mustard seeds), remain scarce and expensive, future strategies should prioritize improving access to these foods or promote cultivation and consumption of alternative source of ALA such as perilla or camelina, alongside optimized fat consumption (120).

### 4.3 Grains and tubers

Although we were not able to clearly distinguish between whole and refined grains within the reviewed studies, grain consumption in South Asia is exceptionally high, primarily due to cultural traditions and economic constraints, as families prioritize affordable sources of energy (52, 53). However, given the established role of whole grains in reducing the risk of diabetes, obesity, cancer, and cardiovascular disease (CVDs), their promotion and partial replacement of refined grains is essential (121, 122).

On the other hand, although total carbohydrate intake generally falls within or slightly exceeds recommendations, it is predominantly derived from refined rice and wheat. Tubers, traditionally considered a nutritionally inferior starch source, are limited to 50–100 g/day in EAT-Lancet’s recommendations, representing only 5% of the suggested grain intake (37). However, South Asia has a diverse range of starchy roots, which in some regions are more accessible than grains (123, 124). Given their comparable or superior nutritional profile to refined rice, we incorporated them within the grain category in our dietary framework, provided that whole grain consumption is also emphasized.

### 4.4 Sweets and ultra-processed food

South Asia has undergone a marked shift toward ultra-processed food consumption, particularly in urban settings and among younger populations. While traditional homemade foods remain prevalent, the consumption of packaged snacks, processed sweets, and ready-to-eat meals has increased significantly over the past two decades, driven by urbanization, economic growth, and shifts in occupational structures (8).

Given the deep cultural significance of sweets in South Asia, a pragmatic approach is warranted. Unlike Western desserts, many traditional South Asian sweets incorporate nutrient-dense ingredients, including dairy, nuts, seeds, legumes, and whole grains. Additionally, while the Mediterranean diet recommends limiting sweet consumption to two servings per week, ultra-processed food intake in South Asia remains lower than in Western populations (6, 50). Instead, moderation and reformulation, such as reducing added sugars and avoiding deep frying, could allow for the continued integration of culturally significant sweets within a balanced dietary pattern, supported by regular physical activity.

However, this flexibility does not extend to industrially processed sweets and salty snacks, whose consumption, particularly in North India, has increased significantly and is strongly linked to rising metabolic disorders (6). These products, characterized by excessive sugar and salt, unhealthy fats (hydrogenated or partially hydrogenated oils), and artificial additives, while being poor in fiber, micronutrients, and antioxidants, contribute to an increasingly adverse dietary profile. Despite Indian dietary guidelines advocating for home-cooked meals and minimally processed foods to improve dietary quality, rapid food industry expansion and shifting consumption patterns are accelerating the erosion of traditional diets. Accordingly, ultra-processed foods are positioned at the top of our dietary pyramids, with a minor differentiation between vegetarians and non-vegetarians. Given that vegetarians should have greater intake of fibers, micronutrients and antioxidants, a slightly more flexible approach to ultra-processed food consumption may be considered, provided that dietary adequacy is maintained.

### 4.5 Strengths and implications

In our methodological framework, we began from the assumption that dietary transitions in rapidly evolving regions can be interpreted through three main domains—economic, cultural, and environmental. However, during the scoping phase, these domains consistently expressed themselves through six recurrent aspects: affordability and modernization on the economic side, cultural traditions and nutritional awareness on the cultural side, and climate change and food contamination on the environmental side. Importantly, these six aspects did not emerge as discrete categories but as interconnected components, with many studies addressing multiple barriers—explicitly or implicitly—within the same analysis.

Starting from an economic perspective, we firstly observed that affordability remains a major constraint: nutrient-rich diets are out of reach for most households, as shown in Uttar Pradesh where 75% could not afford them under current prices. In light of this structural limitation, a first set of solutions concerns measures capable of directly lowering the cost of healthy foods. Policies supporting home food production, subsidized staples, and targeted social protection could reduce costs substantially and improve access to healthier diets (53). Sustainability and economic feasibility must go hand in hand if dietary improvements are to be realistic. For this reason, beyond short-term affordability, broader food-system strategies become essential. Public policies should move beyond caloric sufficiency and explicitly prioritize nutrient density, particularly in food distribution programs that still rely heavily on refined grains. To achieve this, food production policies should align with climate-smart agriculture, incorporating drought-resistant crops, precision irrigation, and agroecological practices to ensure both sustainability and resilience to environmental stressors. Fortification and biofortification also remain among the most cost-effective strategies to improve micronutrient status at scale (90, 125). In parallel, evidence from Bhutan, India, and Pakistan highlights the underutilized potential of wild edible plants (WEPs), which can serve as cost-free, nutrient-dense foods while simultaneously supporting biodiversity conservation in rural areas (126–129).

Improving diets in South Asia is not only about nutrients but also about cultural feasibility and social structures. The PODOSA trial showed that interventions are more effective when they build on familiar foods—by adjusting portion sizes, cooking methods, and ingredients—rather than imposing entirely new dietary patterns (130). Equally important is women’s empowerment, which consistently emerges as a key driver of dietary diversity and food security. Women’s education, financial independence, and agricultural training directly improve household nutrition, while programs such as microfinance initiatives, kitchen gardens, and women-led cooperatives have already proven effective in expanding access to nutrient-dense foods and strengthening community resilience. However, to maximize impact, these efforts must be better integrated into nutrition and food security policies, making gender-inclusive strategies a core component of public health interventions (71, 131–133).

Finally, nutrition awareness must be expanded through a combination of school-based nutrition programs, community workshops, and targeted campaigns. While such initiatives have demonstrated clear benefits, they often struggle to achieve sustainability without dedicated funding and policy support (134). Mobile health (mHealth) platforms can play a complementary role by delivering low-cost, tailored education, particularly to younger and urban populations. Combining these approaches offers a practical pathway to make dietary guidance both accessible and culturally relevant (134).

Food safety and environmental contamination are also critical barriers to healthier diets in South Asia. Pesticide overuse, polluted water, and heavy metal accumulation in crops undermine both nutritional quality and public health. Strengthening regulatory frameworks, investing in improved food storage and irrigation infrastructure, and promoting clean agricultural practices are crucial to mitigating these risks (90, 97, 135, 136).

Taken together, these findings show that improving diets in South Asia requires an approach that goes beyond describing barriers or listing nutritional recommendations, and instead integrates structural constraints directly into practical guidance. The adapted pyramids we developed—grounded in quantitative evidence from the systematic review and refined through qualitative insights from the scoping review—already incorporate the region’s economic, cultural, and environmental realities into a single, feasible model of dietary change. Their structure reflects both nutritional adequacy and real-world feasibility, showing how theoretical guidelines can be translated into culturally acceptable and economically realistic patterns. Yet even a well-integrated framework cannot achieve its intended impact unless the broader food system enables individuals to put these recommendations into practice.

Ensuring these conditions demands coordinated action across multiple actors: governments to align agricultural, fiscal, and regulatory measures with nutritional goals; public health institutions to sustain education and community outreach; agricultural and environmental agencies to support resilient production systems; and civil society organizations—including women’s groups and local cooperatives—to mediate uptake at the community level. As such, the effectiveness and durability of the outcomes proposed in this review rely on a multi-actor governance framework capable of translating behavioral recommendations into stable, long-term improvements in dietary quality and population health.

Despite the analysis being specific to South Asia, we believe that the organizational pattern that emerged—where three overarching domains consistently resolved into a coherent six-dimension structure—reflects a stable configuration rather than a regional peculiarity. Patterns of this kind tend to recur in settings undergoing nutritional and social transition, suggesting that the framework developed here can offer future researchers a robust and transferable basis for interpreting dietary barriers and designing context-specific adaptations in other regions facing similar pressures. While the six barriers themselves appear to be broadly generalizable, their concrete manifestations differ across populations; for this reason, applying the framework elsewhere requires a dedicated scoping review to identify the local problems through which these six underlying dimensions are expressed, and to develop solutions that respond to those specific realities.

### 4.6 Study limitations

Although this study provides a thorough framework, certain limitations should be acknowledged. First, the heterogeneity of dietary data across South Asian countries remains a challenge. While national surveys exist for India, data from other countries are often scarce, outdated, or derived from small, non-representative samples, which may affect the broader applicability of our findings. In particular, the absence of eligible studies from Bhutan, Afghanistan, and the Maldives further limits regional representativeness. While the dietary patterns of Bhutan and the Maldives are likely to share several features with neighboring countries such as India and Bangladesh, Afghanistan presents a more distinct cultural and food-system profile, which may influence the transferability of some adaptation rules. Generalizing to all South Asian countries therefore remains difficult, and the proposed framework could be adapted nationally to reflect sub-regional differences. Regional collaborations aimed at harmonizing dietary assessment methodologies would help generate more consistent and representative evidence. Second, the quality and methodology of the included studies introduced some constraints. Many relied on 24-h recalls or food frequency questionnaires, which are prone to recall bias and reporting inaccuracies, and inconsistencies across tools limited direct comparisons. A shift toward standardized and validated methods, potentially supported by digital technologies, could enhance reliability and precision. Third, the scoping review component, while complementary to the systematic review, included qualitative sources such as non-peer-reviewed food databases, online recipes, and informal consultations with native students, which inherently lack methodological rigor. Fourth, our analysis did not directly evaluate dietary adherence, metabolic outcomes, or long-term feasibility. While the framework provides a structured adaptation of MD principles, its effectiveness in practice remains to be tested. Intervention trials and longitudinal studies are needed to assess adherence, health impacts, and cultural acceptance.

Finally, the study did not fully address regional disparities in food accessibility, pricing, and environmental constraints, which significantly influence feasibility. Incorporating economic modeling and sustainability assessments in future research would help guide policymakers toward interventions that balance nutritional adequacy, affordability, and long-term viability.

## 5 Conclusion

This study presents a structured and replicable approach for adapting the MD to South Asia, addressing nutritional gaps, cultural acceptability, and practical feasibility. Unlike broader continental frameworks, our work delivers a region-specific model that integrates local food systems, traditional practices, and metabolic risk profiles. The resulting pyramids emphasize reducing refined carbohydrate reliance, promoting whole grains and high-quality protein sources, and optimizing fat choices in line with local availability.

While this framework provides a solid foundation, long-term effectiveness will depend on complementary measures. Policy action, targeted education campaigns, and economic and agricultural incentives are essential to make these adaptations affordable and scalable. Future research should test their real-world impact through intervention trials and longitudinal studies, with careful attention to regional disparities in food accessibility.

A sustainable dietary transition in South Asia, aligned with MD principles, will require coordinated efforts among researchers, policymakers, and public health stakeholders. Coordinated efforts across research, policy, and practice will be essential to ensure that dietary recommendations translate into tangible improvements in population health.

## Data Availability

The original contributions presented in the study are included in the article/Supplementary material, further inquiries can be directed to the corresponding author.
