# Supplementary material for: South Asia-specific adaptation of Mediterranean diet principles: a mixed-methods review for practical and sustainable dietary habits
Source: Front Nutr. 2025 Dec 23;12:1719686. doi: 10.3389/fnut.2025.1719686 (PMC12786337; doi:10.3389/fnut.2025.1719686)
Supplement: Supplementary file 1 [file Table_1.DOCX]

**Supplementary file 1. Boolean operators utilized for the systematic review search**

(("India"[Title/Abstract]) OR ("South Asia"[Title/Abstract]) OR ("Bangladesh"[Title/Abstract]) OR ("Afghanistan"[Title/Abstract]) OR ("Sri Lanka"[Title/Abstract]) OR ("Pakistan"[Title/Abstract]) OR ("Maldives"[Title/Abstract]) OR ("Bhutan"[Title/Abstract]) OR ("Nepal"[Title/Abstract])) AND (("food"[Title/Abstract]) OR ("dietary"[Title/Abstract]) OR ("nutrient"[Title/Abstract])) AND (("intake"[Title/Abstract]) OR ("frequency"[Title/Abstract]) OR ("consumption" OR ("deficiency"[Title/Abstract])) AND ("2010/01/01"[Date - Publication] : "2023/12/31"[Date - Publication]) NOT ((children[Title/Abstract]) OR (pregnant[Title/Abstract]) OR (gestational[Title/Abstract]) OR (adolescent[Title/Abstract])))
