# Supplementary material for: South Asia-specific adaptation of Mediterranean diet principles: a mixed-methods review for practical and sustainable dietary habits
Source: Front Nutr. 2025 Dec 23;12:1719686. doi: 10.3389/fnut.2025.1719686 (PMC12786337; doi:10.3389/fnut.2025.1719686)
Supplement: Supplementary file 2 [file Table_2.DOC]

**Supplementary File 2. Newcastle-Ottawa Scale (NOS) adapted for cross-sectional studies**

**Selection:** (Maximum 3 stars)

1) Representativeness of the sample:

a) Truly representative of the average in the target population. * (all subjects or random sampling)

b) Somewhat representative of the average in the target population. * (non-random sampling)

c) Selected group of users.

d) No description of the sampling strategy.

2) Sample size:

a) Justified and satisfactory. *

b) Not justified.

3) Non-respondents:

a) Comparability between respondents and non-respondents characteristics is established, and the response rate is satisfactory. *

b) The response rate is unsatisfactory, or the comparability between respondents and non-respondents is unsatisfactory.

c) No description of the response rate or the characteristics of the responders and the non-responders.

**Control of confounders:** (Maximum 2 stars)

1) The subjects in different outcome groups are comparable, based on the study design or analysis. Confounding factors are controlled:

a) When needed, the study controls for the most important factor (i.e. the study divides the sample based on localization or socio-economic status) *

b) The study controls for any additional control factors.**

c) No subdivision of the sample and no control or discussion of potential confounders.

**Outcome:** (Maximum 4 stars)

1) Assessment of the outcome:

a) Structured interview and outcome based on a quantitative food frequency questionnaire. **

b) Structured interview and outcome based on a qualitative food frequency questionnaire or 24h recall. *

c) Self report.

d) No description.

2) Statistical test:

a) The statistical test used to analyze the data is clearly described and appropriate, and the measurement of the association is presented, including confidence intervals and the probability level (p-value), when applicable. *

b) The statistical test is not appropriate, not described or incomplete.

3) Ascertainment of the outcome measurement:

a) Validated measurement tool *

b) Non-validated measurement tool (i.e. estimate exclusively based on family expenditure for each food group , but the tool is available or described.

c) No description of the measurement tool.

This scale has been adapted from the Newcastle-Ottawa Quality Assessment Scale for cohort studies to perform a quality assessment of cross-sectional studies for the systematic review, “Are Healthcare Workers’ Intentions to Vaccinate Related to their Knowledge, Beliefs and Attitudes? A Systematic Review” (1)

We have substituted the “comparability” with the “control of confounders” factor since both the variables and target population are not the same in each study, but it was important to differentiate the outcomes within multiple populations, whenever it was possible.

In our scale, we have assigned a maximum of nine stars rather than ten stars, as is usual for NOS, since the overall quality of cross sectional studies is lower compared to cohort studies and case-control studies.

**REFERENCES**

1. Herzog R, José Álvarez-Pasquin M, Díaz C, Luis J, Barrio D, Estrada JM, Gil Á. Are healthcare workers’ intentions to vaccinate related to their knowledge, beliefs and attitudes? a systematic review*. BMC Public Heal*th (2013) 13: doi: 10.1186/1471-2458-13-154
