# Supplementary material for: South Asia-specific adaptation of Mediterranean diet principles: a mixed-methods review for practical and sustainable dietary habits
Source: Front Nutr. 2025 Dec 23;12:1719686. doi: 10.3389/fnut.2025.1719686 (PMC12786337; doi:10.3389/fnut.2025.1719686)
Supplement: Supplementary file 3 [file Table_3.DOCX]

| Author | Year | Q 1 | Q2 | Q3 | Q4 | Q5 | Q6 | Q7 | TOT |
| --- | --- | --- | --- | --- | --- | --- | --- | --- | --- |
| Soofi | 2017 | 1 | 1 | 0 | 2 | 1 | 1 | 1 | 7 |
| Vijay | 2021 | 1 | 1 | 0 | 1 | 0 | 1 | 1 | 5 |
| Weerasekara | 2020 | 1 | 1 | 1 | 1 | 1 | 1 | 1 | 7 |
| Ali | 2021 | 1 | 1 | 1 | 2 | 2 | 1 | 1 | 9 |
| Shrestha | 2017 | 1 | 1 | 1 | 1 | 2 | 1 | 1 | 8 |
| Waid | 2018 | 1 | 1 | 1 | 2 | 1 | 1 | 1 | 8 |
| Sharma | 2020 | 1 | 1 | 0 | 1 | 1 | 1 | 1 | 6 |
| Al Hasan | 2019 | 1 | 1 | 0 | 0 | 0 | 1 | 0 | 3 |
| Al Hasan | 2020 | 1 | 1 | 0 | 0 | 0 | 1 | 0 | 3 |
| Anand | 2019 | 1 | 1 | 0 | 2 | 1 | 1 | 1 | 7 |
| Mehroosh | 2022 | 1 | 1 | 0 | 1 | 0 | 1 | 0 | 4 |
| Johnson | 2017 | 1 | 1 | 0 | 1 | 0 | 1 | 0 | 4 |
| Neupane | 2019 | 1 | 1 | 1 | 0 | 0 | 1 | 0 | 4 |
| Ravi | 2016 | 1 | 1 | 0 | 2 | 1 | 1 | 1 | 7 |
| Sowmya | 2016 | 1 | 1 | 0 | 1 | 2 | 1 | 1 | 7 |
| Smith | 2019 | 1 | 1 | 0 | 2 | 2 | 1 | 1 | 8 |
| Jayawardena | 2016 | 1 | 1 | 1 | 1 | 2 | 1 | 1 | 8 |
| Jayawardena | 2014 | 1 | 1 | 0 | 1 | 1 | 1 | 1 | 6 |
| Sivaprasad | 2015 | 1 | 1 | 1 | 1 | 0 | 1 | 0 | 5 |
| Mahajan | 2013 | 1 | 1 | 1 | 1 | 1 | 1 | 1 | 7 |
| Coleman | 2023 | 1 | 1 | 1 | 1 | 1 | 1 | 0 | 6 |
| Sudha | 2020 | 1 | 1 | 0 | 2 | 2 | 1 | 1 | 8 |
| Shridhar | 2014 | 0 | 1 | 1 | 1 | 1 | 0 | 1 | 5 |
| Bhargava | 2014 | 1 | 1 | 1 | 1 | 1 | 1 | 1 | 7 |
| Radhika | 2010 | 1 | 1 | 0 | 1 | 2 | 1 | 1 | 7 |
| Anjana | 2015 | 1 | 1 | 0 | 2 | 2 | 1 | 1 | 8 |
| Shabnam | 2021 | 1 | 1 | 0 | 1 | 1 | 1 | 0 | 5 |
| Zavos | 2024 | 1 | 1 | 0 | 1 | 1 | 1 | 0 | 5 |
| Harris-Fry | 2018 | 1 | 1 | 0 | 2 | 1 | 1 | 1 | 7 |

**Supplementary file 3. Newcastle Ottawa quality assessment for systematic review (adapted for cross sectional studies)**
