# Supplementary material for: South Asia-specific adaptation of Mediterranean diet principles: a mixed-methods review for practical and sustainable dietary habits
Source: Front Nutr. 2025 Dec 23;12:1719686. doi: 10.3389/fnut.2025.1719686 (PMC12786337; doi:10.3389/fnut.2025.1719686)
Supplement: Supplementary file 4 [file Table_4.DOCX]

**Supplementary File 4. Systematic review results on food and nutrient intakes in South Asia**

| Author and year | Localization | Participants/inclusion criteria | Exclusion criteria | Source of information | Food intakes | Nutrient intakes | Newcastle/Ottawa quality score | Other info |
| --- | --- | --- | --- | --- | --- | --- | --- | --- |
| Shrestha et al. 2017 (25) | Central Nepal | 121 participants (37 men; 79 women) from the Dhulikhel Heart  Study (DHS) | Pregnancy | Semiquantitative FFQ and 24h recall | Food intakes (servings/week):  Whole grains 1.71  Refined grains 20.61  Oil 11.73  Butter/ghee 1.04 Vegetables 30.75  Fruits 18.17  Starchy vegetables: 10.10  Meat 4.74  Milk 1.38  Dairies 0.65 | / | 8 | Western food and soft drinks were not frequently consumed. |
| Mehroosh et al.2022 (36) | India | Data from 58 878 urban Indian households from the demographically representative household expenditure panel collected by the Kantar-World panel Division | / | Estimation from national purchase records | Apparent intakes:  Salt: around 10g/day  Cooking oils: 34g/day | / | 4 | Low purchase clusters had the greatest rate of purchase of commonly consumed food (rice, atta, oil, tea, coffee and spices), while high purchase cluster  had a higher rate for milk, butter, cheese, drinks, breakfast cereals and frozen foods.  Purchase of ready to eat foods and sweet  snacks and had increased between 2013 and 2016 across all urban India by 22% an 15% respectively. Ultra-processed foods purchase was found to be increased in all regions except Southern India. Ultra-processed food purchase was still low in Urban India (6.4 kg per household  Member per year) but it had increased by 6% between  2013 and 2016, with the greatest increase in Northern India. |
| Sudha et al. 2020 (61) | India | 463 participants (216 men, 247 women) randomly chosen through a stratified multistage sampling | / | Semiquantitative FFQ | Food intakes (g/day):  Refined cereals: from 198 to 203  Whole grains: from 56.9 to 71.4  Pulses and legumes: from 40.2 to 44.6  Milk and milk products: from 224 to 260  Fats and edible oils: from 36.2 to 38.9  Fruits: from 124 to 134  Leafy vegetables: from 13.2 to 18.2  Other vegetables: from 31.3 to 46.8  Roots and tubers: from 194 to 248  Animal foods: from 25.7 to 28.1 | Energy (kcal/day): from 2302 to 2478  Nutrient intakes (g/day):  Carbohydrates: from 364 to 372  Fats 65.9 to 72.7 Proteins from 61.9 to 65.4  Fiber: from 29.9 to 34.9  PUFA: from 15.0 to 16.8 | 8 | / |
| Vijay et al. 2021 (17) | India | 548806 women from the 2016 National Family Health Survey 4 (NFHS-4) | / | self report qualitative FFQ | Daily consumption of food groups:  Dark green leafy vegetables: 60.2%  Pulses and beans: 57.1%  Milk or curd: 57.4%  Fruits: 15.9%  Fish: 7.1 %  Eggs: 5.1%  Chicken or meat: 1.4% | / | 5 | More than 90% of women were having a dietary diversity score lower than 5. Highest adequate dietary diversity was found in Goa (7.08%), the lowest in Rajasthan (0.11%) |
| Permani C. Weerasekara 2020 (18) | Sri Lanka | 400 women | / | Multiple 24h recalls | / | Nutrient intakes:  Energy: 2230 kcal/day  Carbohydrates: 300 g/day  Fats: 58 g/day  Proteins: 23.4 g/day  Iron: 7.2 mg/day  Calcium 220 mg/day  Zinc 1.2 mg/day  Vit A 180 μg/day  Vit B1 0.98 mg/day  Vit B2 0.99 mg/day  Vit B3 11.6 mg/day  Vit B6 0.96 mg/day  Vit B12 0.86 μg/day  Folate 48.6 μg/day  Vit C 24.4 mg/day | 7 | In rural areas, more than 70% of women in reproductive age produced their food and 14% collected wild fruit and vegetables. On the other hand, overall the subjects had poor diets with imbalanced  macronutrients and alarmingly low intakes of some important micronutrients. |
| Zakari Ali 2021 (22) | Bangladesh | 800 households representative of the seven broad agroecological zones of Bangladesh, from the Bangladesh Climate Change Adaptation Survey (BCCAS) Round 1 | / | Quantitative 14 days dietary recalls | Fruit and vegetables: 293 g/day | Energy: 2933.5 kcal/day  From Carbohydrates 75.2%  From fats 12.2%  From proteins 9.9% | 9 | The identified dietary patterns consisted of a mix of healthy and potentially less healthy habits. Some patterns exhibited greater dietary diversity, but this was observed only among wealthier population groups Rice alone accounted for more than 2000 kcal/day across the entire sample. |
| Waid et al. 2018 (26) | Bangladesh | 52455 households from the Household [Income and] Expenditure  Surveys (H[I]ES) | Estimated energy intake (in AME)  above or below certain cut-offs | Quantitative 30 or 14 days dietary recalls | Food intakes (g/day):  Starches: from to 663 to 811  Pulses: from 18 to 33  Fish: from 25 to 56  Eggs: from 2 to 9  Meat: from 7 to 33  Vegetables: from 108 to 216  Fruits: from 7 to 68  Dairies: from 16 to 80  Oil: from 9 to 30  Spices: from 28 to 59  Sweets: from7 to 37  Beverages: from 12 to 45 | Energy: from 2468 to 3204 kcal/day | 8 | Adherence to the modern dietary pattern (higher in meat, sugar, oil and beverages) dramatically increased from 1% in 1985 to 62% in 2010, linked to urban areas, higher education and modern utilities. Economic growth and increased household expenditure could drive to greater dietary diversity, shifting from starch-heavy diets to more varied diets with higher vegetable intake but especially for rural areas policies that support both economic growth and diversified agricultural production are warranted. |
| Sharma et al. 2020 (29) | India | Data from the Consumption Expenditure Survey (CES) of a nationally  representative sample of 102000 households from 7469 villages and 5268 urban blocks of India conducted by  the National Sample Survey Organization (NSSO) | Floating populations, foreign nationals and  their domestic servants, soldiers in barracks, and kids in  orphanages | Quantitative 30 or 7 days dietary recalls | Food intakes (kcal/day):  Processed food: from 2014 to 263  Spices: from 35 to 39  Estimated intakes(g/day)^1^:  Grains: from 294 to 365  Vegetables: from 204 to 242  Fruits: from 44 to 73  Milk and dairies: from 237 to 300  Eggs: from 9 to 15  Poultry: from 3 to 4  Fish: 6  Legumes and peanuts: from 20 to 22  Nuts: from 2 to 3  Added fats: from 24 to 31 | Energy (kcal/day):  From 1643 to 3174 depending on monthly per capita consumption expenditure.  Mean values: from 2169 to 2214 | 6 | Currently, India produces and consumes too much rice and sugarcane and too little coarse cereals, pulses, fruits, and vegetables, which have lower environmental impact. Transforming the Indian food system necessitates comprehensive policies addressing public health, nutrition, agriculture, trade, and consumer awareness to promote access to healthier dietary options and enhance environmental sustainability. |
| Al Hasan et al. 2019 (31) | Bangladesh | Apparent data from FAO’s food balance sheets documented in the Food and  Agriculture Organization Corporate Statistical Database(FAOSTAT) 1961-2013 | / | Estimated data | Apparent intakes (g/day):  Cereals: 520.4  Starchy roots: 131.5  Pulses: 18  Fish: 52.6  Eggs: 5.7  Meat: 12.9  Vegetables: 74.8  Fruits: 63.9  Milk: 60  Vegetable oil: 17.4  Animal fat: 0.9  Sugar: 22.1 | / | 3 | Since the late 1970s, in Bangladesh, structural changes in diet included increases in vegetable oils, fish, eggs, meat, milk, vegetables, and fruits consumption, though these remain insufficient for positive health impacts. |
| Al Hasan et al. 2020 (32) | Bangladesh | Apparent data from FAO’s food balance sheets documented in the Food and  Agriculture Organization Corporate Statistical Database(FAOSTAT) 1961-2017 | / | Estimated data | / | Apparent intakes (2017):  Energy (kcal/day): 2596  Carbohydrates: 512.3 g/day, 78.9%  Proteins: 60.3 g/day, 9.9%  Fats: 33.9 g/day, 11.8%  Animal fats: 6.7 g/day  Plant fats: 27.27g/day | 3 | Since the late 1990s, Bangladesh has overcome the significant calorie deficit in its diet. However, the diet still suffers from major issues such as excessive carbohydrate intake, and imbalanced, low-quality protein and fat intake. These dietary inadequacies are likely contributing to the rising rates of overweight and obesity, as well as diet-related non-communicable diseases in the country. |
| Anand et al. 2019 (35) | India (urban New Delhi and rural Haryana) | 1445 participants from the Centre for  Cardiometabolic Risk Reduction in South Asia (CARRS) cohort  study and the Indian Council of Medical Research funded Coronary Heart Disease (CHD) repeat survey hat accepted and provided urine samples | Participants with reported energy intakes < 500 kcal and > 4000 kcal/day | Quantitative 24-h dietary recall | Fruit and vegetables intake:  ≤ 2 times/day: 16.9%  2-3 times/day: 72.9%  ≥ 4 times/day: 1.7% | Energy: 1555 kcal/day  Potassium: 1145 mg/day  Sodium: 2863 mg/day | 7 | Rural participants were consuming less fruit and vegetables and more sodium compared to urban participants. |
| Johnson et al. 2017 (37) | India (New Delhi, Haryana, Andhra Pradesh) | 1395 (722 men; 673 women) randomly stratified participants from selected communities from urban, rural and slum areas different in terms of sociodemographic variability | Creatinine values or clearance out of particular cut-offs | Estimation from 24h urinary samples | Estimated salt intake:  Delhi and Haryana: 9.45 g/day  Andhra Pradesh: 10.41 g/day | / | 4 | Salt consumption in Delhi, Haryana, and Andhra Pradesh is about twice the WHO-recommended maximum of 5 g/day, affecting all demographics. Indian cuisine is high in salt content, particularly in rural pickles and urban fast foods. |
| Neupane et al. 2019 (38) | Nepal | 4510 (155 men; 295 women) participants randomly selected from the modified version of the WHO STEPwise approach to  surveillance (STEPS) | Total 24h urinary volume < 500 mL; estimated daily urinary creatinine excretion < 6 mmol for men and < 4 mmol for women; self reported spillage for more than 30 mL | Estimation from 24h urinary samples | Estimated salt intake:  13.2 g/day | / | 4 | Salt intake in Nepal is more than twice the WHO recommendations. Interventions targeting the marketing, availability, and labeling of processed foods are crucial due to their significant salt content. |
| Ravi et al. 2016 (39) | South India (urban Chennai, semi-urban Thiruvallum, rural Kanchipuram | 6876 (2964 men; 3912 women) participants enrolled in PURSE-HIS  (Population Study of Urban, Rural and Semiurban  Regions for the Detection of Endovascular  Disease and Prevalence of Risk Factors and Holistic  Intervention Study) | Previous history of hypertension and outliers for sodium intake | Quantitative 24h recall | / | Energy Intake:  From 2379 to 3043 kcal/day  Sodium Intake:  From 3.2 to 4.1 g/day | 7 | Dietary sodium intake was higher than recommendations and was independently associated with blood pressure.  Salt intake in South India is mainly dependent on added salt in rice or bread, vegetables, and pulses dishes. |
| Sowmya et al. 2016 (41) | South India (Tamil Nadu) | 6907 (2900 men; 4007 women) participants recruited from the rural component of the Chennai Urban Rural Epidemiological Study 2007-2010 (CURES) | Self-reported history of diabetes or unrealistic energy intake (< 500 and > 3500 kcal/day for women and <800 and > 4000 kcal/day for men) | Semi-quantitative FFQ | Food intakes (g/day):  Refined cereals: from 428.1 to 459.3  Whole milled cereals: from 2.9 to 3.7  Millets: from 3.3 to 4.1  Legumes: from 16.1 to 18.4  Dairies: from 123.5 to 135.4;  Tubers: from 12.7 to 20.7  Fruits and vegetables: from 93.4 to 112.2  Eggs: 20.8  Meat and poultry: from 3.2 to 3.8  Fish and seafood: from 14.0 to 19.4  Fat and oils: from 13.4 to 14.5  Nuts: from 3.9 to 4.8  Added salt: from 4.5 to 5.6 men  Added sugars: from 3.6 to 3.7 women | Energy:  from 1976 to 2151 kcal/day  Nutrient intakes (g/day):  Carbohydrates: from 392.1 to 417  Glycaemic load: from 255.6 to 272.2  Proteins: from 45.8 to 51.3  Fats: from 27.4 to 32.0  Fibers: from 13.8 to 16.4 | 7 | Most of the rural population in South Asia exceed the carbohydrate and salt intake compared to WHO recommendations, while protein, fats (especially monounsaturated and poly unsaturated fats) and fruit and vegetable intake were lower than recommended. |
| Smith et al. 2019 (47) | India | Data from the 2011-2012 7 rounds of the National Sample Survey of Consumption  Expenditure (whole Indian population) | NR | Semiquantitative FFQ | / | Average zinc intake: 8.72 mg/day  Average phytate intake: 1670 mg/day  Average absorbable zinc intake: 2.21 mg/day  Prevalence of zinc intake inadequacy: 24.6% | 8 | The prevalence of inadequate zinc intake is increasing in India and is expected to worsen due to elevated CO2 levels reducing zinc in crops. Without intervention, current dietary trends won't resolve this issue. Effective solutions include direct supplementation, fortification, biofortification, and advocating for zinc-rich diets. Targeted programs for high-risk states and populations are essential. |
| Soofi et al. 2017 (11) | Pakistan | 22278 women in reproductive age (15-49) from the 27963 households that responded to the 2011 National Nutrition Survey (NNS) | NR | Blood tests | / | Prevalence of anaemia: 50.4%  Prevalence of folate deficiency: 50.8 %  Prevalence of vit B12 deficiency: 52.4 % | 7 | Factors associated with iron, folate and vit B12 deficiencies include low socioeconomic status, rural residence, and inadequate diet. Addressing these deficiencies requires interventions such as enhancing health education, dietary diversity, and food fortification. Gender bias and inequitable food distribution within households may also contribute to these deficiencies, necessitating targeted efforts to ensure food security for women. |
| Radhika 2010 (67) | South India | 2042 (917 men; 1125 women) participants recruited from the urban component of the Chennai Urban Rural Epidemiological Study 2005 (CURES) | Self-reported history of diabetes or CVD or unrealistic energy intake (< 500 and > 4200 kcal/day) | Semi-quantitative FFQ | Food intakes (g/day):  Refined cereals: 330.2  Whole milled cereals: 39.5  Millets: 6.7  Legumes: 52.9  Dairies: 393.6  Tubers: 131.1  Fruits and vegetables: 265.0  Eggs: 13.6  Meat and poultry: 49.7  Fish and seafood: 20.0  Fat and oils: 33.6  Nuts: 23.1  Added salt: 5.6 men; 4.5 women  Added sugars: 47.9 | Energy: 2484 kcal/day  Nutrient intakes (%energy):  Carbohydrates: 63.8  Proteins: 12.1  Fats: 23.9  Fibers: 34.7  Alcohol 35.0 | 7 | 2005 data showed that In urban South India there was a low consumption of wholegrain cereals, millets, fish, fruits, and vegetables and high consumption of refined grains. |
| Anjana et al. 2015 (69) | South India | 1376 (573 men; 803 women) individuals from the urban component of the Chennai Urban Rural Epidemiological Study 2001-2003 | Diabetes | Semi-quantitative FFQ | Food intakes (g/day):  Refined cereals: 342.0  Pulses and legumes: 52.6  Dairies: 372.3  Tubers: 24.9  Fruit and vegetables: 337.8  Meats: 18.8  Fish and sea food: 17.2  Nuts and seeds: 20.6  Added fats: 33.1  Added salt: 8.5  Added sugar: 13.3 | Energy: 2528 kcal/day  Nutrients intake (%energy):  Carbohydrates 64.6  Fats: 23.5  Proteins: 11.2  Saturated fat: 8.6  Poly unsaturated fat: 6.5  Monounsaturated fat: 6.9  Fiber: 30.0 g/day  Glycaemic index: 63.1  Glycaemic load: 234.7 | 8 | Over 80% of diabetes cases in an urban South Indian population could be prevented by modifying five key risk factors: obesity, physical inactivity, diet, hypertriglyceridemia, and low HDL cholesterol. Improving diet and physical activity alone could prevent 51.7% of cases, increasing to 70.8% with control of abdominal obesity. |
| Mazumderet al 2013 (65) | West India | 236 Participants (148 men; 88 women) randomly selected from 212 households | Participants with insufficient scalp hair were excluded from the study, as hair samples were needed for arsenic analysis | Quantitative 24-h dietary recall | Food intakes (g/day)  Rice: from 447 to 458  Chapati: from 192 to 212  Other grains: from 24 to 42  Pulses (cooked): from 105 to 141  Vegetables (cooked): from 272 to 281  Milk: from 93 to 126  Fish: from 40 to 48  Meat: 82  Eggs: from 19 to 39  Fruits: 75 | / | 7 | Significant risk of arsenic exposure through diet in regions using arsenic-contaminated groundwater for irrigation and underscores the need for effective mitigation strategies. |
| Shridhar Et al 2014 63 | India | 6555 (3814 men; 2714 women)  participants  from the Indian Migration Study (IMS) | Participants who had improbable energy intake levels <  500 kcal or > 5000 kcal/day, ovo-vegetarians, incomplete FFQ  or physical activity data | Semiquantitative FFQ | / | Energy (kcal/day): from 2712.2 to 2728.8  Nutrients (g/day):  Proteins: from 76.1 to 78.1  Fats: from 74.2 to 75.9  Fibers: from 12.8 to 13.7  Micronutrients (mg/day):  Iron: from 22.4 to 25.5  Calcium: from 946.5 to 980.6  Zinc: 11.6  Vitamin C: from 136.9 to 142.7  Vitamin B12: from 1.2 to 2.2 μg/day  Folate: from 327.2 to 355.6 μg/day | 5 | A small but beneficial association was found between vegetarian diet and cardiovascular risk factors compared to non-vegetarian  Diet across four geographic regions of India |
| Coleman et al 2023 (55) | Bangladesh | 25824 (11797 men; 14027 women) participants from the Tranfer Modality Research (TMR) and the Agricolture, Nutrition and Gender Linkage (ANGeL) projects | / | 24-h dietary recalls | Food predicted probability of adequate consumption (%):  Citrus Fruits: from 0.05 to 1.81  Other fruits: from 5.7 to 7.0  Dark green leafy vegetables: from 25.7 to 35.2  Cruciferous vegetables: from 2.5 to 36.8  Deep orange vegetables: from 2.3 to 11.3  Other vegetables: from 81.1 to 95.3  Legumes: from 26.7 to 29.4  Nuts and seeds: from 0.1 to 1.0  Whole grains: from 17.2 to 27.5  Fish and seafood: from 45.0 to 76.4  Poultry: from 3.35 to 11.8  Low fat diary: from 0.01 to 0.07  High fat diary: from 6.5 to 12.7  Eggs: from 0.01 to 16.6  Red meat: from 2.2 to 5.5 | Energy Intake (kcal/day)  From 2230.2 to 2714.5 | 6 | poor diet quality, driven mostly by high consumption of refined grains and low consumption of healthy food groups, is a pressing concern for men and women across different socioeconomic stratum of rural Bangladesh. |
| Mahajan et al 2013 54 | Northern India | 200 subjects from the Prospective Urban Rural Epidemiology (PURE) study | - | Semiquantitative FFQ and 24h-dietary recalls | / | Energy (kcal/day):  From 1868 to 2147  Nutrient intakes (g/day):  Proteins: from 67.0 to 71.3  Fats: from 39.7 to 50.1  Carbohydrates: from 316.2 to 355.5  Fiber: from 10.0 to 11.8  Micronutrients intake (mg/day):  Vit B1: from 2.1 to 2.3  Vit B2: from 1.5 to 1.7  Vit C: from 39.9 to 87.9  Zinc: from 11.3 to 23.7  Iron: from 11.7 to 25.2  Calcium: from 808.1 to 944.0  β-carotene: from 1063 to 1317 μg/day | 7 | / |
| Sivaprasad  et al. 2015  50 | Central India | 274 (127 men; 147 women) apparently  healthy adults | Multivitamin supplements  for the last 6 months or suffering from severe metabolic  complications or having a history of surgical operation of the gastrointestinal  tract or suffering from acute illness at the time of enrollment | 24h-dietary recalls | / | Dietary vit B12: from 0.73 to 1.31 μg/day  Dietary folates: from 163.1 to 173.5 μg/day | 4 | Only 40% of the study population met 70% of the Recommended Dietary Allowance (RDA) for B12. |
| Ranil Jayawardena et al 2014 49 | Sri Lanka | 463 adult participants (166 men; 297 women) from the Sri Lanka Diabetes and Cardiovascular Study | - | Quantitative 24h recall | - | Energy: from 1439 to 1669 kcal/day  Nutrient intakes (g/day):  Carbohydrates: from 270.3 to 305.9  Proteins: from 42.9 to 47.8  Fats: from 24.8 to 36.1  Fiber: from 17.7 to 20.6  Sodium: from 2729 to 2890 mg/day | 6 | Sri Lankan adults consume high levels of carbohydrates, low protein, low dietary fiber, and high sodium, which may contribute to the rising prevalence of non-communicable diseases such as diabetes and hypertension. |
| Shabnam et al. 2021 (72) | Pakistan | 16341 households from the Household Integrated Economic  Survey 2010-2011 (HIES) | Calorie consumption < 600 and > 8000 kcal/day | Estimated data from food purchases in a 30-day recall | Apparent food intake (g/day)^2^:  Wheat: 266  Rice: 33  Milk: 223  Meat: 17  Pulses: 10  Ghee and oil: 31  Fruits: 51  Vegetables: 92  Tubers: 36  Added sugar: 46  Estimated calories from food groups (%):  Spices: 0.5 | Energy: 2227 kcal/day  Nutrient intakes (g/day):  Carbohydrates: 348.4  Proteins: 55.3  Fats: 65.7  Micronutrient intakes (mg/day):  Calcium: 579.9  Iron: 28.3  Zinc: 10.6  Iodine: 54.4 μg/day  Vit A: 457.2 μg/day | 5 | Increases in  household income in Pakistan appear to translate to greater  consumption of dairies, meats, oils, sugar,  vegetables, and fruits rather than mostly comprising cereals. |
| Jayawardena Et al 2016 (48) | Sri Lanka | 100 non-institutionalized adults randomly selected from the Sri Lanka Diabetes and Cardiovascular Study | / | Semi-quantitative FFQ |  | Energy (kcal/day): 1794.1  Nutrient intakes (g/day):  Proteins: 50.1  Fats: 46.1  Carbohydrates: 303.9  Dietary fiber: 21.8  PUFA: 2.4  Micronutrient intakes (mg/day):  Calcium: 677.4  Magnesium: 308.9  Potassium: 1963  Iron: 19.7  Zinc: 7.3  Vit C: 67.9  Vit E: 2.6  Vit B1: 1.5  Vit B2: 1.2  Vit B6: 1.3  Folic acid: 45.5  Vitamin A: 652.3 μg/day | 8 | Coconut oil is the main source of fat in Sri Lanka |
| Zavos et al. 2024 (73) | Sri Lanka | 3969 (1683 men; 2286 women) participants aged > 16 years from the second wave of the Colombo Twin and Singleton Study (CoTaSS-2) | Participants unable to understand the consent process or the questionnaires due to language barriers or apparent cognitive impairments | Qualitative FFQ | Food intakes (servings/week):  Meat: 1.73  Fish 8.95  Eggs: 1.46  Grains: 8.61  Rice: 18.03  Flour: 3.91  Dairies: 10.76  Salty snacks: 1.88  Sweet snacks: 5.46  Nuts/seeds: 0.79  Fruits: 7.45  Leafy greens: 7.54  Other vegetables: 16.91 | / | 5 | Economic and environmental factors were the main determinants of food consumption in the sample. |
| Harris-Fry et al. 2018 | Nepal | 1278 participants from 150 households in Dhanusha and Mahottari districts | / | 24h dietary recalls | Food intakes (g/day):  Starchy staples: from 886 to 1098  Pulses: from 96 to 113  Nuts and seeds: from 6.3 to 6.7  Dairy: from 240 to 324  Meat and fish: from 48 to 73  Eggs: from 4.8 to 7.9  Green leafy vegetables: from 22 to 25  Vit A rich fruits and vegetables: from 201 to 226  Other vegetables: from 150 to 189  Other fruits: from 32 to 55  Alcohol: from 0 to 45 | Energy (kcal/day): from 2228 to 2841  Protein (g/day): from 67 to 87  Nutrient intakes (mg/day):  Vitamin C: from 128 to 133  Iron: from 15 to 25  Zinc: from 11 to 14  Calcium: from 511 to 686  Folate (μg/day): from 350 to 639  Vit B12 (μg/day): from 0.6 to 0.9  Vitamin A (RE): from 486 to 511 | 7 | Male household  heads consume more animal-source foods, alcohol and processed food resulting in a higher dietary adequacy compared to pregnant women and mothers-in-law. On the other hand, women consume more fruit and vegetables. |

^1^ : Conversion from kcal/day to g/day calculated as the estimation presented in Table 1 in the article: Willett W, Rockström J, Loken B, Springmann M, Lang T, Vermeulen S, Garnett T, Tilman D, DeClerck F, Wood A, et al. Food in the Anthropocene: the EAT–Lancet Commission on healthy diets from sustainable food systems. *Lancet* (2019) 393:447–492. doi: 10.1016/S0140-6736(18)31788-4

^2^ : Verified data taken from the HIES 2010-2011 website: https://www.pbs.gov.pk/publication/household-integrated-economic-survey-hies-2010-11
