# Supplementary material for: South Asia-specific adaptation of Mediterranean diet principles: a mixed-methods review for practical and sustainable dietary habits
Source: Front Nutr. 2025 Dec 23;12:1719686. doi: 10.3389/fnut.2025.1719686 (PMC12786337; doi:10.3389/fnut.2025.1719686)
Supplement: Supplementary file 5 [file Table_5.DOCX]

**Supplementary File 6. Scoping review results for barriers to healthy nutrition in South Asia**

| Author | Year | Country/region | Socioeconomical barriers | | Cultural barriers | | Envirnomental barriers | | Key Findings |
| --- | --- | --- | --- | --- | --- | --- | --- | --- | --- |
|  |  |  | Income levels and cost of nutritious food | Urbanization and modernization | Cultural and religious traditions | Lack of nutritional awareness and education | Climate change | Pollution |  |
| Payyappallimana(1) | 2016 | India | No | No | Yes | No | No | No | Traditional Ayurvedic food practices and principles are impactful in determining food preferences and intake, with mixed outcomes on the population |
| Sarkar(2) | 2015 | India | No | No | Yes | No | No | No | Ayurvedic principles on healthy food are rooted in Indian culture, providing dietary variety but also rigid schemes that need to followed, often without sufficient scientific evidence |
| Pieroni(3) | 2007 | Bangladesh | Yes | No | Yes | No | No | No | Socioeconomic status and cultural beliefs significantly influence dietary habits among South-Asian migrants |
| Amiry(4) | 2022 | Afghanistan | No | No | Yes | Yes | No | No | Gastric cancer is very common in Afghanistan and subjects with higher adherence to the Mediterranean diet show a reduced risk |
| Hewawasam(5) | 2017 | India | No | No | Yes | Yes | No | No | Regional dietary practices present challenges in adopting a low FODMAP diet |
| Kaur (6) | 2018 | India | Yes | No | No | Yes | No | No | Modern information technology may be useful to improve nutritional awareness and education |
| Dhillon(7) | 2016 | India | No | No | Yes | No | No | No | Legume intake does not correlate with lower diabetes prevalence in a population with high legume consumption |
| Kachwaha(8) | 2020 | India | Yes | No | Yes | No | No | No | Financial barriers and dietary preferences, rather than food availability, hinder access to nutritious diets for vulnerable populations |
| Bren d’Amour(9) | 2020 | India | No | Yes | Yes | No | Yes | No | Urbanization and increased processed food consumption affect dietary habits and nutrition |
| Joy(10) | 2017 | India | No | Yes | Yes | Yes | No | No | Traditional dietary practices have cardiovascular benefits, but modern trends pose health risks |
| Kapoor(11) | 2018 | India | Yes | Yes | Yes | Yes | No | No | Dietary patterns in South Asia, including Prudent, Indian, and Non-Vegetarian diets, show that higher dietary diversity is associated with lower cardiometabolic disease risks, while urban dietary shifts expose populations to unhealthy food choices |
| John (12) | 2021 | India | Yes | Yes | Yes | No | No | No | Factors influencing pulse consumption and farming include cultural preferences, availability and affordability |
| Aryal(13) | 2020 | South Asia | Yes | No | No | No | Yes | No | Climate change and lack of financing institutions severely impact food security and livelihoods of smallholder farmers |
| Aggarwal(14) | 2011 | South Asia | Yes | Yes | No | Yes | Yes | No | Climate change will likely to affect food security in South Asia and several actions need to be taken to face its potential negative impact |
| Rasul(15) | 2014 | South Asia | Yes | Yes | Yes | Yes | Yes | Yes | Demographic, economic, and environmental changes in South Asia have increased the demand for natural resources and intensified their uses, with serious implications for food, water, and energy security in the subregion |
| Beal(16) | 2017 | South Asia | No | No | Yes | Yes | No | No | Widespread micronutrient deficiencies impact public health in South Asia |
| Swaminathan(17) | 2019 | India | Yes | No | No | Yes | No | No | Addressing anemia requires more than just iron supplementation; educational and dietary factors are also critical |
| Chaudhary(18) | 2022 | India | Yes | No | Yes | Yes | No | No | Micronutrient deficiencies in India are prevalent and affect overall health |
| Pradhan(19) | 2021 | India | Yes | No | Yes | Yes | No | No | Strategies to improve dietary variety in India face cultural and economic barriers |
| Saquib(20) | 2016 | Bangladesh | Yes | Yes | No | No | No | No | Lifestyle and dietary habits in Bangladesh are influenced by socioeconomic status |
| Bishwajit(21) | 2017 | South Asia | No | No | No | Yes | No | No | Low fruit and vegetable consumption is linked to higher depression rates in South Asia |
| Mustafa(22) | 2022 | India | Yes | Yes | Yes | Yes | No | No | Traditional food patterns in India are shifting due to urbanization and economic changes |
| Shridhar(23) | 2018 | India | Yes | Yes | Yes | Yes | No | No | Dietary patterns of Indian immigrants are influenced by cultural retention and adaptation |
| Ford(24) | 2019 | India | No | Yes | No | Yes | No | No | Effective dietary changes for diabetes prevention face cultural and economic challenges |
| Tarar(25) | 2020 | Pakistan | Yes | Yes | Yes | Yes | No | No | High trans fat consumption in Pakistan poses significant health risks |
| Singh(26) | 2011 | India | Yes | Yes | Yes | No | No | No | Economic constraints and cultural factors influence obesity rates and dietary habits |
| Ravi(27) | 2016 | India | No | Yes | Yes | Yes | No | Yes | Excessive sodium intake in India is linked to increased hypertension and cardiovascular disease |
| Ghimire(28) | 2021 | South Asia | No | Yes | Yes | Yes | No | No | Salt intake in South Asia is about twice the recommended levels and is in excess both during cooking and as discretionary table salt |
| Tripathy(29) | 2016 | India | No | Yes | No | Yes | No | No | Urban-rural differences in dietary patterns affect nutritional status |
| Alae-Carew(30) | 2019 | India | No | Yes | No | Yes | Yes | No | Future changes in food patterns in India are influenced by urbanization and economic factors. |
| Patel(31) | 2006 | India | No | Yes | No | No | No | No | Migration affects coronary heart disease risk factors through dietary changes. |
| Biehl (32) | 2016 | Nepal | Yes | No | No | Yes | Yes | No | Improving diets in Nepal faces financial and logistical challenges |
| Mritunjay (33) | 2016 | India | No | No | No | Yes | No | Yes | High prevalence of pathogens in raw vegetables in India poses health risks |
| Kumar(34) | 2016 | India | No | No | No | No | No | Yes | Salmonella contamination in food is a significant health concern in India. |
| Sultana(35) | 2017 | Pakistan | Yes | No | No | Yes | Yes | Yes | Aflatoxin contamination in rice is a critical issue in Pakistan affecting food safety. |
| Naeem(36) | 2022 | Pakistan | Yes | No | No | Yes | No | Yes | Aflatoxin contamination is widespread in Pakistan, posing health risks |
| Xia(37) | 2022 | Pakistan | Yes | No | No | Yes | Yes | Yes | Mycotoxin contamination in Pakistan's food supply poses serious health risks |
| Aslam(38) | 2021 | Pakistan | No | No | No | Yes | Yes | Yes | Patulin contamination in fruits is a concern for food safety in Pakistan |
| Iqbal(39) | 2016 | Pakistan | Yes | No | No | Yes | Yes | Yes | Contamination of rice with aflatoxins and ochratoxins affects food safety in Pakistan |
| Zakir (40) | 2022 | Bangladesh | No | No | No | Yes | Yes | Yes | Arsenic contamination in water and food is a severe health issue in Bangladesh |
| Islam (41) | 2017 | Bangladesh | No | No | No | Yes | Yes | Yes | High levels of arsenic in rice affect food safety and public health in Bangladesh |
| Hassan(42) | 2017 | South Asia | No | No | No | Yes | Yes | Yes | Arsenic exposure is linked to increased diabetes prevalence in South Asia. |
| Real(43) | 2017 | Bangladesh | Yes | Yes | No | Yes | No | Yes | Heavy metal contamination in food and water poses health risks in Bangladesh |
| Ullah (44) | 2017 | Bangladesh | Yes | Yes | No | Yes | No | Yes | Significant levels of heavy metals in food and water impact public health in Bangladesh |
| Alamdar(45) | 2017 | Pakistan | Yes | No | No | Yes | No | Yes | Heavy metal contamination in fish is a health concern in Pakistan |
| Jose (46) | 2018 | India | Yes | Yes | No | Yes | No | Yes | Widespread heavy metal contamination in food and water affects health in India |
| Hussain(47) | 2022 | Pakistan | Yes | No | No | Yes | No | Yes | Heavy metal contamination in Pakistan poses health risks. |
| Ullah(48) | 2022 | Pakistan | Yes | No | No | Yes | No | Yes | Heavy metals in the food supply of Pakistan are a public health concern |
| Kulathunga(49) | 2022 | Sri Lanka | Yes | No | No | Yes | No | Yes | Heavy metal contamination in Sri Lanka affects food safety and health |
| Lin Liu(50) | 2020 | Sri Lanka | Yes | No | No | Yes | No | Yes | Elevated levels of heavy metals in food impact health in Sri Lanka |
| Forsyth(51) | 2019 | Bangladesh | Yes | No | Yes | Yes | No | Yes | Lead contamination in turmeric is a serious health risk in Bangladesh |
| Ahmad(52) | 2015 | Afghanistan | Yes | No | No | Yes | No | Yes | Heavy metal contamination in Afghanistan poses significant health risks |
| Malakar(53) | 2016 | India | No | Yes | No | Yes | Yes | Yes | Increasing arsenic contamination in groundwater is a major environmental challenge in Kolkata, India. There is an urgent need for treated water supply and better groundwater management practices to prevent arsenic from entering the food chain and affecting public health |
| Khan(54) | 2017 | Pakistan | Yes | No | No | Yes | No | Yes | Potatoes grown on metal-contaminated soils in Punjab, Pakistan, pose significant health risks due to heavy metal accumulation. The study underscores the need for soil remediation and monitoring to ensure food safety |
| Haque(55) | 2018 | Bangladesh | Yes | No | Yes | Yes | No | Yes | Significant amounts of DDT metabolites were found in the serum of women in reproductive age in Bangladesh, especially in people with higher intakes of meat and fish |
| Ali(56) | 2022 | Pakistan | No | No | No | Yes | No | Yes | Some samples of Okra in Pakistan are not compliant with European Union maximum residue limits for pesticides, resulting in a small risk for acute exposure but insignificant for chronic exposure |
| Al-Mamun(57) | 2019 | Bangladesh | No | No | No | Yes | Yes | Yes | Polychlorinated biphenyls (PCBs) are present at medium-to-high concentrations in seafood in Bangladesh, presumably due to continuing and historical usage, resulting in a significant health risk |
| Arshad(58) | 2022 | Pakistan | No | No | No | Yes | Yes | Yes | Bioaccumulation of Polychlorinated biphenyls (PCBs) in the food chains affect grains, beans and vegetables in Pakistan, posing a moderate level of risk for human health |
| Sharma(59) | 2022 | India | No | No | No | Yes | Yes | Yes | Green chili and okra have the highest presence of pesticides in India, but their concentration is considered safe |
| Agarwal(60) | 2015 | India | No | No | No | Yes | Yes | Yes | Bioaccumulation of certain pesticides in the food chain, water and soil are alarming threats for the future, even if those levels are currently considered safe |
| Ahmed(61) | 2016 | India | No | No | No | Yes | Yes | Yes | Contamination by PCBs, especially in marine fish, poses a lifetime cancer risk to consumers of fish in India |
| Scheelbeek(62) | 2017 | Bangladesh | Yes | No | No | Yes | Yes | Yes | Drinking water salinity in coastal Bangladesh is extremely high, raising the overall daily sodium intake and blood pressure |
| Bhathal(63) | 2020 | India | Yes | No | Yes | Yes | No | No | Spices in India are crucial for traditional cuisine and have health benefits, but are mostly consumed for their flavor and are more utilized in urban areas |
| Siruguri(64) | 2015 | India | No | No | Yes | No | No | No | Intake of spices varies based on the traditional dishes that are prepared |
| Isharwal(65) | 2008 | India | No | Yes | No | Yes | No | No | Dietary nutrients are linked to insulin resistance among urban adolescents |
| Gulati(66) | 2013 | India | No | Yes | Yes | Yes | No | No | Familial dietary habits correlate with obesity rates in urban India |
| Whitton(67) | 2018 | India | No | Yes | Yes | Yes | No | No | Healthy dietary patterns correlate with reduced cardiovascular risk factors |
| Radhika(68) | 2008 | India | Yes | Yes | Yes | Yes | No | No | Fruit and vegetable intake is associated with lower cardiovascular risk |
| Venkatesan(69) | 2023 | India | No | Yes | Yes | Yes | No | No | Dietary patterns significantly affect health outcomes in South Asia |
| Harinarayan(70) | 2021 | India | Yes | No | Yes | Yes | No | No | Calcium and Vitamin D deficiency in India are frequent and there is an urging need of government action to reduce this type of malnutrition |
| Sikorski(71) | 2023 | South Asia | Yes | Yes | Yes | Yes | No | No | Dietary consumption patterns are changing rapidly in South Asia |
| Daniel(72) | 2014 | India | No | Yes | No | Yes | No | No | A dietary assessment tool and nutrient database for India could be useful for improving nutritional status and education |
| Neelakantan(73) | 2016 | India | No | Yes | No | Yes | No | No | A food frequency questionnaire for multi-ethnic Asian populations with educational purpose |
| Lim(74) | 2021 | India | No | Yes | Yes | Yes | No | No | Higher diet quality and lower refined grain consumption are linked to less weight gain |
| Colles(75) | 2013 | India | Yes | Yes | Yes | Yes | No | No | Dietary beliefs and eating patterns influence metabolic health in type 2 diabetes |
| Mohan(76) | 2009 | India | No | Yes | Yes | Yes | No | No | High glycemic load diets are associated with type 2 diabetes risk in urban Asian Indian populations |
| Safdar(77) | 2013 | Pakistan | Yes | No | Yes | Yes | No | No | Dietary habits in Pakistan are influenced by socioeconomic status and cultural practices |
| Abeywickrama(78) | 2020 | Sri Lanka | Yes | No | Yes | Yes | Yes | No | Dietary intakes in Sri Lanka vary significantly by region and socioeconomic status |
| Parthasarathy(79) | 2015 | India | Yes | No | No | Yes | No | No | Dietary modifications can improve micronutrient status in Indian children and adolescents with type 1 diabetes |
| Iqba(80) | 2009 | India | No | Yes | Yes | Yes | No | No | Food frequency questionnaire developed and validated for South Indian populations |
| Mustafa(81) | 2021 | Bangladesh | Yes | No | Yes | Yes | No | No | Fruit and vegetable consumption in Bangladesh is associated with socioeconomic status |
| Ojha(82) | 2022 | Nepal | Yes | No | Yes | Yes | Yes | No | Traditional diets in the Himalayas are influenced by local food availability and cultural practices |
| Mumu(83) | 2020 | Bangladesh | Yes | No | Yes | Yes | No | No | Dietary intakes in Bangladesh show significant regional variations |
| Abeywickrama (84) | 2018 | Sri Lanka | Yes | Yes | No | Yes | No | No | Micronutrient deficiencies are prevalent in Sri Lanka, especially in rural areas |
| Shalini(85) | 2019 | India | Yes | Yes | No | Yes | No | No | Micronutrient deficiencies are common in South India, with significant health impacts |
| Nair(86) | 2018 | India | No | Yes | Yes | Yes | No | No | High sodium intake in West India is linked to increased hypertension risk |
| Sudha(87) | 2006 | India | No | Yes | Yes | Yes | No | No | Dietary patterns in South India are shifting towards more processed foods |
| S Shetty(88) | 2002 | India | Yes | Yes | Yes | Yes | No | No | Food consumption trends in India show a shift towards more processed and convenience foods |
| Vaidya(89) | 2022 | India | Yes | Yes | No | No | No | No | Vitamin K intake in India is insufficient, particularly among vegetarians |
| Riaz(90) | 2016 | Pakistan | No | Yes | Yes | Yes | No | No | Vitamin D deficiency is highly prevalent in Pakistan, with significant health impacts |
| Fahim(91) | 2017 | Afghanistan | Yes | No | Yes | Yes | Yes | No | Dietary survey in Afghanistan highlights significant nutritional deficiencies |
| Sudo(92) | 2009 | Nepal | Yes | No | No | Yes | Yes | No | Dietary patterns in Nepal are influenced by socioeconomic status and cultural practices |
| Chakraborty(93) | 2009 | India | Yes | Yes | No | Yes | No | No | Dietary patterns in Kolkata are influenced by urbanization and economic status |
| Singh(94) | 2011 | India | Yes | Yes | Yes | Yes | No | No | Dietary patterns in Punjab are influenced by migration and socioeconomic changes |
| Mark(95) | 2016 | South Asia | Yes | Yes | Yes | Yes | No | Yes | Micronutrient deficiencies are widespread in South Asia, with significant health impacts |
| Chen(96) | 2004 | Bangladesh | Yes | Yes | No | Yes | No | No | Food frequency questionnaire validated for large cohort studies in Bangladesh highlights significant nutritional deficiencies |
| Bharathi(97) | 2008 | India | Yes | Yes | Yes | Yes | No | No | Food frequency questionnaire validated for South Indian populations |
| Sudo(98) | 2006 | India | No | No | Yes | Yes | No | No | Dietary patterns in Hindu communities in India are influenced by religious practices. |
| Ryckman(99) | 2021 | South Asia | Yes | Yes | Yes | Yes | No | No | Intake of certain nutrients in South Asia is dependent on the prices of their main dietary sources |
| Anjana(100) | 2014 | India | Yes | Yes | Yes | Yes | No | No | A large percentage of Indian population is inactive and only 10% is engaged in recreational physical activity |
| Jayawardena(101) | 2020 | South Asia | Yes | No | Yes | Yes | No | No | Low consumption of fruits and vegetables among South Asians is associated with a high prevalence of micronutrient deficiencies, influenced by cultural preferences and economic barriers that limit dietary diversity |
| Soofi(102) | 2017 | Pakistan | Yes | No | No | Yes | No | No | Women of reproductive age in Pakistan face high deficiencies in vitamin B12, folate, and iron, primarily due to low dietary diversity and limited awareness of micronutrient-rich food sources |
| Vijay(103) | 2021 | India | Yes | Yes | No | Yes | No | No | Dietary diversity among Indian women is very low, particularly in low-income households, with economic disparities and limited education acting as major barriers to accessing a variety of nutrient-rich foods |
| Weerasekara(104) | 2020 | Sri Lanka | Yes | Yes | Yes | Yes | No | No | Dietary diversity in Sri Lanka is lower in urban compared to rural areas, with economic barriers and the erosion of traditional food systems contributing to nutritional inadequacies in marginal and disadvantaged populations |
| Ali(105) | 2021 | Bangladesh | Yes | No | Yes | Yes | Yes | No | Dietary patterns in Bangladesh are dominated by staple-based diets that are inadequate for nutritional requirements, with economic vulnerability and cultural factors shaping food consumption behaviors |
| Lin(106) | 2017 | Bangladesh | Yes | No | Yes | Yes | No | No | Rural populations in Bangladesh rely heavily on staple foods and exhibit low dietary diversity, restricted by economic constraints and limited education, with food frequency questionnaires developed to tailor dietary assessments |
| Shrestha(107) | 2017 | Nepal | Yes | No | Yes | Yes | No | No | Nepalese populations show poor dietary variety, influenced by economic barriers and lack of nutritional awareness |
| Waid(108) | 2019 | Bangladesh | Yes | Yes | Yes | Yes | Yes | No | Dietary patterns in Bangladesh have shifted from traditional diets with mustard oil and pulses to modern, rice-dominated diets influenced by urbanization and economic growth. Limited awareness and economic constraints restrict access to nutrient-rich foods, with cultural and seasonal factors further shaping food choices |
| Sharma(109) | 2020 | India | Yes | Yes | No | Yes | No | No | In India, a significant disparity emerges in the intake of micronutrient-rich foods, particularly among economically weaker groups. Urban diets show increasing reliance on processed foods |
| Al Hasan(110) | 2019 | Bangladesh | Yes | No | Yes | Yes | Yes | No | Bangladeshi diets rely heavily on rice, with low intake of fruits, vegetables, and proteins. Cultural preferences and economic constraints limit dietary diversity, while regional differences reflect climatic and agricultural influences |
| Al Hasan(111) | 2020 | Bangladesh | Yes | Yes | Yes | Yes | No | No | Persistent high intake of rice dominates diets with inadequate protein and micronutrient consumption |
| Anand(112) | 2020 | India | No | Yes | Yes | Yes | No | No | Low potassium intake across rural and urban areas, with dietary reliance on high-sodium foods increasing hypertension risks |
| Tak(113) | 2022 | India | Yes | Yes | No | Yes | No | No | Urban households increasingly purchase processed foods, with higher consumption among wealthier households. Socioeconomic factors correlate with ultraprocessed food intake |
| Johnson(114) | 2017 | India | No | Yes | Yes | Yes | No | No | Average salt intake across rural and urban India exceeds WHO recommendations, driven by traditional dietary practices involving homemade food recipes |
| Neupane(115) | 2020 | Nepal | No | Yes | Yes | Yes | No | No | Salt consumption is nearly double WHO-recommended levels, largely due to habitual dietary patterns and lack of awareness |
| Sowmya(116) | 2016 | India | Yes | No | Yes | Yes | No | No | Diets are dominated by refined cereals and lack fruits, vegetables, and polyunsaturated fatty acids, correlating with higher rates of NCDs |
| Siddiquee(117) | 2021 | South Asia | Yes | Yes | Yes | Yes | No | No | Vitamin D deficiency is prevalent across South Asia, especially among women. Factors include limited dietary diversity and inadequate awareness |
| Smith(118) | 2019 | India | Yes | Yes | Yes | Yes | Yes | Yes | Urban areas in India have a higher prevalence of inadequate zinc intake compared to rural areas, driven by lower grain consumption and higher zinc requirements due to older populations. Shifts from coarse cereals to refined grains and rising CO2 levels are predicted to worsen zinc inadequacy |
| Jayawardena(119) | 2016 | Sri Lanka | No | No | Yes | Yes | No | No | Diets in Sri Lanka rely heavily on starchy foods, providing over 70% of energy intake, and are linked to high rates of diabetes and non-communicable diseases. Limited dietary diversity reflects cultural habits favoring staples over nutrient-rich foods |
| Jayawardena(120) | 2014 | Sri Lanka | No | No | Yes | Yes | No | No | Diets in Sri Lanka show low intake of micronutrient-rich foods and high consumption of refined carbohydrates. Significant nutritional gaps are linked to chronic diseases, emphasizing the need to address these deficiencies |
| Sivaprasad(121) | 2016 | India | No | Yes | Yes | Yes | No | No | A high prevalence of Vitamin B12 deficiency in urban South Indian adults is associated with dietary habits, including vegetarianism, which limits intake of animal-derived nutrients |
| Mahajan(122) | 2013 | India | Yes | No | Yes | Yes | No | No | Refined FFQ in South India provides reliable estimates of dietary intake but highlights gaps in nutrient adequacy, especially in rural populations relying on less diverse diets |
| Coleman(123) | 2023 | Bangladesh | Yes | No | Yes | Yes | Yes | No | Rural Bangladesh populations face significant micronutrient deficiencies due to poor dietary diversity and limited access to fortified foods |
| Akhtar(124) | 2013 | South Asia | Yes | No | Yes | Yes | Yes | No | Widespread micronutrient deficiencies in South Asia are linked to reliance on refined grains and insufficient public health initiatives targeting diverse diets |
| Sudha(125) | 2020 | India | Yes | Yes | Yes | Yes | No | No | Urbanization in India increases the consumption of processed and unhealthy foods, with a decline in traditional diets rich in whole grains and pulses |
| Shridhar(126) | 2014 | India | No | No | Yes | Yes | No | No | Vegetarian dietary preferences in India limit protein and micronutrient intake, increasing deficiencies in iron and Vitamin B12 among certain populations |
| Mazumder(127) | 2013 | India | Yes | No | No | Yes | No | Yes | Groundwater arsenic contamination in West Bengal severely affects dietary safety and health, reducing access to clean water and safe food preparation methods |
| Radhika(128) | 2010 | India | Yes | Yes | Yes | Yes | No | No | Refined grains and high glycemic load are significantly associated with increased risk of type 2 diabetes in urban Asian Indian populations while fiber intake is inversely associated with the risk |
| Anjana(129) | 2015 | India | No | Yes | Yes | Yes | No | No | Behavioral interventions and dietary awareness campaigns targeting lifestyle factors and eating habits are critical in managing diabetes prevalence in rapidly modernizing urban areas |
| Shabnam(130) | 2021 | Pakistan | Yes | No | Yes | Yes | No | Yes | High prevalence of vitamin D deficiency in Pakistan is linked to dietary inadequacies, limited awareness, and air pollution affecting sunlight exposure |
| Zavos(131) | 2024 | Sri Lanka | No | No | Yes | Yes | No | No | Food frequency patterns reveal inadequate consumption of fruits and vegetables despite their availability. Cultural preferences and lack of education on the health benefits contribute to suboptimal dietary diversity |
| Harris-Fry(132) | 2018 | Nepal | Yes | No | Yes | Yes | Yes | No | Rural Nepali communities face challenges in dietary diversity due to economic constraints, limited agricultural output, and climate-induced crop variations |
| Sathyamala(133) | 2018 | India | Yes | No | Yes | Yes | No | No | Vegetarianism in India is deeply rooted in Hinduism tradition and in the whole culture, resulting in potential law imposed prohibitions, social frictions and under-reporting of meat, fish or eggs consumption |
| Khan(134) | 2015 | Pakistan | Yes | No | No | Yes | Yes | Yes | Willingness to pay more for reducing pesticides risk in Pakistan is associated with educational level and awareness |
| Upadhyay(135) | 2011 | India | Yes | Yes | Yes | Yes | No | No | Lack of improvement in agricultural productivity, lack of education, inadequate job opportunities, overpopulation, gender inequality, inadequate distribution of food and lack of coherent nutrition policies are key factors leading to malnutrition in India |
| Sraboni(136) | 2018 | Bangladesh | Yes | No | Yes | Yes | No | No | Women empowerment is associated with better nutritional diversity and quality in Bangladesh, but with gender inequality |
| Srinivasan(137) | 2009 | India | Yes | Yes | No | Yes | Yes | Yes | The use of untreated or partially untreated wastewater for irrigation in India is associated with higher morbidity rates |
| Rao(138) | 2018 | India | Yes | No | Yes | No | No | No | Agricultural productivity is associated with higher intakes of grains and higher body mass index for women farmers in rural India |
| Rahman(139) | 2014 | Bangladesh | Yes | No | No | Yes | Yes | No | Bangladesh has made good progress towards food security by increasing productivity, but malnutrition is still highly prevalent for some key nutrients |
| Jha(140) | 2009 | India | Yes | No | No | Yes | No | No | Poverty-Nutrition Trap exists in rural India for energy intake and some micronutrients, being responsible of a malnutrition vicious cycle |
| Nithya(141) | 2018 | India | Yes | No | No | Yes | No | No | Dietary diversity is linked to an higher nutritional status among rural India adults and adolescents |
| Nahar(142) | 2013 | Bangladesh | Yes | No | Yes | Yes | No | Yes | Poverty and stigmatization of pig raisers are barriers to implement health interventions to reduce the risk of some zoonoses in Bangladesh |
| Balagamwala(143) | 2015 | Pakistan | Yes | No | Yes | No | No | No | For women working in agriculture in Pakistan an inverse relationship exists between farming production and family care, impacting the nutritional quality of family and children |
| Mulmi(144) | 2017 | Nepal | Yes | No | No | Yes | No | No | In poor communities of Nepal, child dietary intake is associated with the composition of farm production, often resulting in a reduced dietary diversity |
| Mulmi(145) | 2016 | Nepal | Yes | No | No | Yes | Yes | Yes | Lack of proper sanitation and hygiene is a barrier towards an healthy children growth in Nepal. More robust food markets protect children against fluctuations in local food production due to climate change |
| Aich(146) | 2014 | Bangladesh | Yes | No | Yes | Yes | No | No | Women farmers and shrimp workers in Bangladesh suffer from multiple nutritional deficiencies due to economic and cultural causes |
| Mohsena(147) | 2018 | Bangladesh | Yes | No | No | Yes | Yes | No | Income, education and agro-ecological aspects conditioning food production impact dietary quality in Bangladesh |
| Misra(148) | 2018 | Bangladesh | Yes | Yes | No | Yes | No | No | Agricultural modernization and commercialization reproduce rural malnutrition by degrading local biodiversity and the rural poor’s access to nutrient-rich diets in Bangladesh |
| Malapit(149) | 2015 | Nepal | No | No | Yes | Yes | No | No | Women empowerment effectively reduce the risk of malnutrition of both women and children by increasing dietary and farming products diversity |
| Naz(150) | 2014 | Pakistan | No | No | Yes | Yes | No | No | Severe inequalities exist between rural men and women in Pakistan in terms of food security and decisional role, impacting the overall health of families |
| Komatsu(151) | 2018 | Bangladesh & Nepal | Yes | No | Yes | Yes | No | No | Women in Nepal tend to dedicate more time to agricultural work compared to Bangladeshi, who instead spend more time for domestic work. Time spent for cooking is positively associated with women and children dietary diversity |
| Joshi(152) | 2018 | India | Yes | No | No | Yes | No | No | Inadequate dietary intake, especially hidden hunger and Chronic Energy Deficiency was prevalent among rural women in India |
| Kadiyala(153) | 2014 | India | Yes | No | No | No | No | No | Agricultural production, income and diet quality are deeply intertwined in rural India |
| Gulati(154) | 2012 | India | Yes | No | No | Yes | No | No | Improvements in agriculture alone is not enough in combating malnutrition in India. Interventions to improve education, health, sanitation and household infrastructure, and care and feeding practices are critical |
| Gartaula(155) | 2017 | Nepal | Yes | No | Yes | Yes | No | No | Agricultural changes in Nepal improve food security through off-farm income but increase burdens on women managing households. The "food wellbeing" approach reveals social and subjective factors affecting small-scale farming sustainability amid reliance on non-agricultural income. |
| Flores-Martinez(156) | 2016 | Afghanistan | Yes | No | No | Yes | No | No | Own-production of mutton may have a role to play in improving iron intakes and lowering anemia risk in Afghanistan |
| Cunningham(157) | 2015 | Nepal | No | No | Yes | Yes | No | No | Some aspects of women’s empowerment in agriculture are associated with better young child nutritional status and healthy growth in rural Nepal |
| Broaddus-Shea(158) | 2018 | Nepal | Yes | No | Yes | Yes | Yes | No | Children’s diets vary differently by season within each agro-ecological zone of Nepal and in some cases across socioeconomic groups, revealing the importance of taking a season- and location-specific approach to assessing diets and tailoring dietary strategies |
| Vijaya Bhaskar(159) | 2017 | India | Yes | No | No | Yes | Yes | No | Nutritional status, dietary diversity and cropping patterns in both Wardha and Koraput regions of India reflected the influence of local socio-economic and environmental aspects, farm household decision making and the choice and opportunities of farming approaches |

**REFERENCES**

1. Payyappallimana U, Venkatasubramanian P. Exploring ayurvedic knowledge on food and health for providing innovative solutions to contemporary healthcare. *Front Public Heal* (2016) 4:1–9. doi: 10.3389/fpubh.2016.00057

2. Sarkar P, Lohith KDH, Dhumal C, Panigrahi SS, Choudhary R. Traditional and ayurvedic foods of Indian origin. *J Ethn Foods* (2015) 2:97–109. doi: 10.1016/j.jef.2015.08.003

3. Pieroni A, Houlihan L, Ansari N, Hussain B, Aslam S. Medicinal perceptions of vegetables traditionally consumed by South-Asian migrants living in Bradford, Northern England. *J Ethnopharmacol* (2007) 113:100–110. doi: 10.1016/j.jep.2007.05.009

4. Amiry F, Mousavi SM, Barekzai AM, Esmaillzadeh A. Adherence to the Mediterranean Diet in Relation to Gastric Cancer in Afghanistan. *Front Nutr* (2022) 9:1–6. doi: 10.3389/fnut.2022.830646

5. Hewawasam SP, Iacovou M, Muir JG, Gibson PR. Dietary practices and FODMAPs in South Asia: Applicability of the low FODMAP diet to patients with irritable bowel syndrome. *J Gastroenterol Hepatol* (2018) 33:365–374. doi: 10.1111/jgh.13885

6. Kaur J, Kaur M, Webster J, Kumar R. Protocol for a cluster randomised controlled trial on information technology-enabled nutrition intervention among urban adults in Chandigarh (India): SMART eating trial. *Glob Health Action* (2018) 11: doi: 10.1080/16549716.2017.1419738

7. Dhillon PK, Bowen L, Kinra S, Bharathi AV, Agrawal S, Prabhakaran D, Reddy KS, Ebrahim S, Patel T, Ramakrishnan L, et al. Legume consumption and its association with fasting glucose, insulin resistance and type 2 diabetes in the Indian Migration Study. *Public Health Nutr* (2016) 19:3017–3026. doi: 10.1017/S1368980016001233

8. Kachwaha S, Nguyen PH, DeFreese M, Avula R, Cyriac S, Girard A, Menon P. Assessing the economic feasibility of assuring nutritionally adequate diets for vulnerable populations in Uttar Pradesh, India: Findings from a “cost of the diet” analysis. *Curr Dev Nutr* (2020) 4:1–9. doi: 10.1093/cdn/nzaa169

9. Bren d’Amour C, Pandey B, Reba M, Ahmad S, Creutzig F, Seto KC. Urbanization, processed foods, and eating out in India. *Glob Food Sec* (2020) 25:100361. doi: 10.1016/j.gfs.2020.100361

10. Joy EJM, Ahmad W, Zia MH, Kumssa DB, Young SD, Ander EL, Watts MJ, Stein AJ, Broadley MR. Valuing increased zinc (Zn) fertiliser-use in Pakistan. *Plant Soil* (2017) 411:139–150. doi: 10.1007/s11104-016-2961-7

11. Kapoor D, Iqbal R, Singh K, Jaacks LM, Shivashankar R, Sudha V, Anjana RM, Kadir M, Mohan V, Ali MK, et al. Association of dietary patterns and dietary diversity with cardiometabolic disease risk factors among adults in South Asia: The CARRS study. *Asia Pac J Clin Nutr* (2018) 27:1332–1343. doi: 10.6133/apjcn.201811_27(6).0021

12. John AT, Makkar S, Swaminathan S, Minocha S, Webb P, Kurpad A V., Thomas T. Factors influencing household pulse consumption in India: A multilevel model analysis. *Glob Food Sec* (2021) 29:100534. doi: 10.1016/j.gfs.2021.100534

13. Aryal JP, Sapkota TB, Khurana R, Khatri-Chhetri A, Rahut DB, Jat ML. *Climate change and agriculture in South Asia: adaptation options in smallholder production systems*. Springer Netherlands. (2020). 5045–5075 p. doi: 10.1007/s10668-019-00414-4

14. Aggarwal PK, Sivakumar MVK. *Climate Change and Food Security in South Asia*. (2011). doi: 10.1007/978-90-481-9516-9

15. Rasul G. Food, water, and energy security in South Asia: A nexus perspective from the Hindu Kush Himalayan region{star, open}. *Environ Sci Policy* (2014) 39:35–48. doi: 10.1016/j.envsci.2014.01.010

16. Beal T, Massiot E, Arsenault JE, Smith MR, Hijmans RJ. Global trends in dietary micronutrient supplies and estimated prevalence of inadequate intakes. *PLoS One* (2017) 12:1–20. doi: 10.1371/journal.pone.0175554

17. Swaminathan S, Ghosh S, Varghese JS, Sachdev HS, Kurpad A V., Thomas T. Dietary iron intake and anemia are weakly associated, limiting effective iron fortification strategies in India. *J Nutr* (2019) 149:831–839. doi: 10.1093/jn/nxz009

18. Chaudhary V, Saraswathy K, Sarwal R. Dietary diversity as a sustainable approach towards micronutrient deficiencies in India. *Indian J Med Res* (2022) 156:31–45. doi: 10.4103/IJMR.IJMR_3314_21

19. Pradhan A, Raju S, Nithya DJ, Panda AK, Wagh RD, Maske MR, Bhavani R V. Farming System for Nutrition-a pathway to dietary diversity: Evidence from India. *PLoS One* (2021) 16:1–20. doi: 10.1371/journal.pone.0248698

20. Saquib J, Saquib N, Stefanick ML, Khanam MA, Anand S, Rahman M, Chertow GM, Barry M, Ahmed T, Cullen MR. Sex differences in obesity, dietary habits, and physical activity among urban middle-class Bangladeshis. *Int J Health Sci (Qassim)* (2016) 10:363. doi: 10.12816/0048730

21. Bishwajit G, O’Leary DP, Ghosh S, Sanni Y, Shangfeng T, Zhanchun F. Association between depression and fruit and vegetable consumption among adults in South Asia. *BMC Psychiatry* (2017) 17:1–9. doi: 10.1186/s12888-017-1198-1

22. Mustafa U, Ghoshal UC. The challenges of implementing low fermentable oligo-, di-, mono-saccharides and polyol diet in India: An analysis of available data. *Indian J Gastroenterol* (2022) 41:319. doi: 10.1007/s12664-022-01268-x

23. Shridhar K, Satija A, Dhillon PK, Agrawal S, Gupta R, Bowen L, Kinra S, Bharathi A V., Prabhakaran D, Srinath Reddy K, et al. Association between empirically derived dietary patterns with blood lipids, fasting blood glucose and blood pressure in adults - The India migration study. *Nutr J* (2018) 17:1–12. doi: 10.1186/s12937-018-0327-0

24. Ford CN, Weber MB, Staimez LR, Anjana RM, Lakshmi K, Mohan V, Narayan KMV, Harish R. Dietary changes in a diabetes prevention intervention among people with prediabetes: the Diabetes Community Lifestyle Improvement Program trial. *Acta Diabetol* (2019) 56:197–209. doi: 10.1007/s00592-018-1249-1

25. Tarar OM, Ahmed KM, Nishtar NA, Achakzai ABK, Gulzar Y, Delles C, Al-Jawaldeh A. Understanding the complexities of prevalence of trans fat and its control in food supply in Pakistan. *J Clin Hypertens* (2020) 22:1338–1346. doi: 10.1111/jch.13943

26. Singh M, Kirchengast S. Obesity Prevalence and nutritional habits among Indian women: a comparison between Punjabi women living in India and Punjabi migrants in Vienna, Austria. *J Biol Clin Anthropol* (2011) 68:239–251. doi: 10.1127/0003-5548/2011/0132

27. Ravi S, Bermudez OI, Harivanzan V, Kenneth Chui KH, Vasudevan P, Must A, Thanikachalam S, Thanikachalam M. Sodium Intake, Blood Pressure, and Dietary Sources of Sodium in an Adult South Indian Population. *Ann Glob Heal* (2016) 82:234–242. doi: 10.1016/j.aogh.2016.02.001

28. Ghimire K, Mishra SR, Satheesh G, Neupane D, Sharma A, Panda R, Kallestrup P, Mclachlan CS. Salt intake and salt-reduction strategies in South Asia: From evidence to action. *J Clin Hypertens* (2021) 23:1815–1829. doi: 10.1111/jch.14365

29. Tripathy JP, Thakur JS, Jeet G, Chawla S, Jain S, Prasad R. Urban rural differences in diet, physical activity and obesity in India: Are we witnessing the great Indian equalisation? Results from a cross-sectional STEPS survey. *BMC Public Health* (2016) 16:1–10. doi: 10.1186/S12889-016-3489-8/FIGURES/1

30. Alae-Carew C, Bird FA, Choudhury S, Harris F, Aleksandrowicz L, Milner J, Joy EJ, Agrawal S, Dangour AD, Green R. Future diets in India: A systematic review of food consumption projection studies. *Glob Food Sec* (2019) 23:182–190. doi: 10.1016/j.gfs.2019.05.006

31. Patel J V., Vyas A, Cruickshank JK, Prabhakaran D, Hughes E, Reddy KS, Mackness MI, Bhatnagar D, Durrington PN. Impact of migration on coronary heart disease risk factors: Comparison of Gujaratis in Britain and their contemporaries in villages of origin in India. *Atherosclerosis* (2006) 185:297–306. doi: 10.1016/j.atherosclerosis.2005.06.005

32. Biehl E, Klemm RDW, Manohar S, Webb P, Gauchan D, West KP. What Does It Cost to Improve Household Diets in Nepal? Using the Cost of the Diet Method to Model Lowest Cost Dietary Changes. *Food Nutr Bull* (2016) 37:247–260. doi: 10.1177/0379572116657267

33. Mritunjay SK, Kumar V. Microbial quality, safety, and pathogen detection by using quantitative PCR of raw salad vegetables sold in Dhanbad City, India. *J Food Prot* (2017) 80:121–126. doi: 10.4315/0362-028X.JFP-16-223

34. Kumar Y, Gupta N, Vaish VB, Gupta S. Distribution trends & antibiogram pattern of Salmonella enterica serovar Newport in India. *Indian J Med Res* (2016) 144:82–86. doi: 10.4103/0971-5916.193293

35. Sultana N, Tahira I, Kausar M, Hassan SM, Hanif NQ. Dietary exposure and natural occurrence of total aflatoxins in basmati rice of Pakistan. *J Food Prot* (2017) 80:331–337. doi: 10.4315/0362-028X.JFP-16-290

36. Naeem I, Ismail A, Rehman AU, Ismail Z, Saima S, Naz A, Faraz A, de Oliveira CAF, Benkerroum N, Aslam MZ, et al. Prevalence of Aflatoxins in Selected Dry Fruits, Impact of Storage Conditions on Contamination Levels and Associated Health Risks on Pakistani Consumers. *Int J Environ Res Public Health* (2022) 19: doi: 10.3390/ijerph19063404

37. Xia L, Rasheed H, Routledge MN, Wu H, Gong YY. Super-Sensitive LC-MS Analyses of Exposure Biomarkers for Multiple Mycotoxins in a Rural Pakistan Population. *Toxins (Basel)* (2022) 14:1–16. doi: 10.3390/TOXINS14030193

38. Aslam K, Iqbal SZ, Faizal A, Razis A, Usman S, Ali NB. Variation of patulin levels in citrus fruits from central cities of Punjab and Northern cities of Pakistan, and estimation of dietary intake. *Int J Environ Res Public Health* (2021) 18: doi: 10.3390/ijerph18052270

39. Iqbal SZ, Asi MR, Hanif U, Zuber M, Jinap S. The presence of aflatoxins and ochratoxin A in rice and rice products; And evaluation of dietary intake. *Food Chem* (2016) 210:135–140. doi: 10.1016/j.foodchem.2016.04.104

40. Zakir HM, Quadir QF, Bushra A, Sharmin S, Sarker A, Rashid MH, Rahman A. Human health exposure and risks of arsenic from contaminated soils and brinjal fruits collected from different producers and retailers levels. *Environ Geochem Health* (2022) 44:4665–4683. doi: 10.1007/s10653-022-01227-7

41. Islam S, Rahman MM, Islam MR, Naidu R. Geographical variation and age-related dietary exposure to arsenic in rice from Bangladesh. *Sci Total Environ* (2017) 601–602:122–131. doi: 10.1016/j.scitotenv.2017.05.184

42. Hassan FI, Niaz K, Khan F, Maqbool F, Abdollahi M. The Relation Between Rice Consimption, Arsenic Contamination, and Prevalence of Diabetes in South Asia. *EXCLI J* (2017) 16:1132–1143. doi: 10.17179/excli2017-222

43. Real MIH, Azam HM, Majed N. Consumption of heavy metal contaminated foods and associated risks in Bangladesh. *Environ Monit Assess* (2017) 189: doi: 10.1007/s10661-017-6362-z

44. Ullah AKMA, Maksud MA, Khan SR, Lutfa LN, Quraishi SB. Dietary intake of heavy metals from eight highly consumed species of cultured fish and possible human health risk implications in Bangladesh. *Toxicol Reports* (2017) 4:574–579. doi: 10.1016/j.toxrep.2017.10.002

45. Alamdar A, Eqani SAMAS, Hanif N, Ali SM, Fasola M, Bokhari H, Katsoyiannis IA, Shen H. Human exposure to trace metals and arsenic via consumption of fish from river Chenab, Pakistan and associated health risks. *Chemosphere* (2017) 168:1004–1012. doi: 10.1016/j.chemosphere.2016.10.110

46. Jose A, Ray JG. Toxic heavy metals in human blood in relation to certain food and environmental samples in Kerala, South India. *Environ Sci Pollut Res* (2018) 25:7946–7953. doi: 10.1007/s11356-017-1112-x

47. Hussain N, Shafiq ahmed K, Asmatullah, shafiq Ahmed M, Makhdoom Hussain S, javid A. Potential health risks assessment cognate with selected heavy metals contents in some vegetables grown with four different irrigation sources near Lahore, Pakistan. *Saudi J Biol Sci* (2022) 29:1813–1824. doi: 10.1016/j.sjbs.2021.10.043

48. Ullah H, Khaliq M, Ullah N, Iqbal A, Fozia, Ullah I. Health Risk Assessment and Multivariate Statistical Analysis of Heavy Metals in Vegetables of Khyber Pakhtunkhwa Region, Pakistan. *Biol Trace Elem Res* (2022) 200:3023–3038. doi: 10.1007/s12011-021-02892-y

49. Kulathunga MRDL, Wijayawardena MAA, Naidu R. Dietary heavy metal(loid)s exposure and prevalence of chronic kidney disease of unknown aetiology (CKDu) in Sri Lanka. *Environ Geochem Health* (2022) 44:3863–3874. doi: 10.1007/s10653-021-01144-1

50. Liu L, Han J, Xu X, Xu Z, Abeysinghe KS, Atapattu AJ, De Silva PMCS, Lu Q, Qiu G. Dietary exposure assessment of cadmium, arsenic, and lead in market rice from Sri Lanka. *Environ Sci Pollut Res* (2020) 27:42704–42712. doi: 10.1007/s11356-020-10209-0

51. Forsyth JE, Nurunnahar S, Islam SS, Baker M, Yeasmin D, Islam MS, Rahman M, Fendorf S, Ardoin NM, Winch PJ, et al. Turmeric means “yellow” in Bengali: Lead chromate pigments added to turmeric threaten public health across Bangladesh. *Environ Res* (2019) 179:108722. doi: 10.1016/j.envres.2019.108722

52. Ahmad H, Yousafzai AM, Siraj M, Ahmad R, Ahmad I, Nadeem MS, Ahmad W, Akbar N, Muhammad K. Pollution problem in River Kabul: Accumulation estimates of heavy metals in native fish species. *Biomed Res Int* (2015) doi: 10.1155/2015/537368

53. Malakar A, Islam S, Ali MA, Ray S. Rapid decadal evolution in the groundwater arsenic content of Kolkata, India and its correlation with the practices of her dwellers. *Environ Monit Assess* (2016) 188: doi: 10.1007/s10661-016-5592-9

54. Khan ZI, Ahmad K, Yasmeen S, Akram NA, Ashraf M, Mehmood N. Potential health risk assessment of potato (Solanum tuberosum L.) grown on metal contaminated soils in the central zone of Punjab, Pakistan. *Chemosphere* (2017) 166:157–162. doi: 10.1016/j.chemosphere.2016.09.064

55. Haque R, Inaoka T, Fujimura M, Watanabe C, Ahmad AS, Kakimoto R, Ishiyama M, Ueno D. Dietary patterns and serum of DDT concentrations among reproductive-aged group of women in Bangladesh. *Environ Sci Pollut Res* (2018) 25:17665–17673. doi: 10.1007/s11356-018-1958-6

56. Ali SN, Rafique N, Akhtar S, Taj T, Mehboob F. Analysis of multiple pesticide residues in market samples of okra and associated dietary risk assessment for consumers. *Environ Sci Pollut Res* (2022) 29:47561–47570. doi: 10.1007/s11356-022-19197-9

57. Al-Mamun H, Ahmed K, Islam S, Hossain A, Tokumura M, Masunaga S. Polychlorinated biphenyls (PCBs) in commonly consumed seafood from the coastal area of Bangladesh: occurrence, distribution, and human health implications. *Environ Sci Pollut Res* (2019) 26:1355–1369. doi: 10.1007/s11356-018-3671-x

58. Arshad M, Asmatullah, Shafiq Ahmed M, Shafiq ahmed K, Makhdoom Hussain S, Hussain N. Monitoring of level of mean concentration and toxicity equivalence (TEQ) of polychlorinated biphenyls (PCBs) in selected vegetables, beans and grains in khanewal and multan, Pakistan. *Saudi J Biol Sci* (2022) 29:2787–2793. doi: 10.1016/j.sjbs.2022.01.009

59. Sharma KK, Tripathy V, Sharma K, Gupta R, Yadav R, Devi S, Walia S. Long–term monitoring of 155 multi–class pesticide residues in Indian vegetables and their risk assessment for consumer safety. *Food Chem* (2022) 373:131518. doi: 10.1016/j.foodchem.2021.131518

60. Agarwal A, Prajapati R, Singh OP, Raza SK, Thakur LK. Pesticide residue in water—a challenging task in India. *Environ Monit Assess* (2015) 187: doi: 10.1007/s10661-015-4287-y

61. Ahmed MN, Sinha SN, Vemula SR, Sivaperumal P, Vasudev K, Ashu S, Mendu VVR, Bhatnagar V. Accumulation of polychlorinated biphenyls in fish and assessment of dietary exposure: a study in Hyderabad City, India. *Environ Monit Assess* (2016) 188:1–11. doi: 10.1007/s10661-015-5068-3

62. Scheelbeek PFD, Chowdhury MAH, Haines A, Alam DS, Hoque MA, Butler AP, Khan AE, Mojumder SK, Blangiardo MAG, Elliott P, et al. Drinking water salinity and raised blood pressure: Evidence from a cohort study in coastal Bangladesh. *Environ Health Perspect* (2017) 125:1–8. doi: 10.1289/EHP659

63. Bhathal SK, Kaur H, Bains K, Mahal AK. Assessing intake and consumption level of spices among urban and rural households of Ludhiana district of Punjab, India. *Nutr J* (2020) 19:1–12. doi: 10.1186/s12937-020-00639-4

64. Siruguri V, Bhat R V. Assessing intake of spices by pattern of spice use, frequency of consumption and portion size of spices consumed from routinely prepared dishes in southern India. *Nutr J* (2015) 14:1–9. doi: 10.1186/1475-2891-14-7

65. Isharwal S, Arya S, Misra A, Wasir JS, Pandey RM, Rastogi K, Vikram NK, Luthra K, Sharma R. Dietary nutrients and insulin resistance in urban Asian Indian adolescents and young adults. *Ann Nutr Metab* (2008) 52:145–151. doi: 10.1159/000127416

66. Gulati S, Misra A, Colles SL, Kondal D, Gupta N, Goel K, Bansal S, Mishra M, Madkaikar V, Bhardwaj S. Dietary intakes and familial correlates of overweight/obesity: A four-cities study in India. *Ann Nutr Metab* (2013) 62:279–290. doi: 10.1159/000346554

67. Whitton C, Rebello SA, Lee J, Tai ES, van Dam RM. A healthy asian a posteriori dietary pattern correlates with a priori dietary patterns and is associated with cardiovascular disease risk factors in a multiethnic asian population. *J Nutr* (2018) 148:616–623. doi: 10.1093/jn/nxy016

68. Radhika G, Sudha V, Mohan Sathya R, Ganesan A, Mohan V. Association of fruit and vegetable intake with cardiovascular risk factors in urban south Indians. *Br J Nutr* (2008) 99:398–405. doi: 10.1017/S0007114507803965

69. Venkatesan P, Prakash SS, Ramasamy J. Association of dietary patterns and health outcomes by spatial regression analysis of nationally representative survey data from India. *Indian J Public Health* (2023) 67:399–407. doi: 10.4103/ijph.ijph_112_23

70. Harinarayan CV, Akhila H, Shanthisree E. Modern India and Dietary Calcium Deficiency—Half a Century Nutrition Data—Retrospect–Introspect and the Road Ahead. *Front Endocrinol (Lausanne)* (2021) 12: doi: 10.3389/fendo.2021.583654

71. Sikorski C, Yang S, Stennett R, Miller V, Teo K, Anand SS, Paré G, Yusuf S, Dehghan M, Mente A. Changes in energy, macronutrient, and food consumption in 47 countries over the last 70 years (1950-2019): a systematic review and meta-analysis. *Nutrition* (2023) 108: doi: 10.1016/j.nut.2022.111941

72. Daniel CR, Kapur K, McAdams MJ, Dixit-Joshi S, Devasenapathy N, Shetty H, Hariharan S, George PS, Mathew A, Sinha R. Development of a field-friendly automated dietary assessment tool and nutrient database for India. *Br J Nutr* (2014) 111:160–171. doi: 10.1017/S0007114513001864

73. Neelakantan N, Whitton C, Seah S, Koh H, Rebello SA, Lim JY, Chen S, Chan MF, Chew L, Van Dam RM. Development of a semi-quantitative food frequency questionnaire to assess the dietary intake of a multi-ethnic urban asian population. *Nutrients* (2016) 8:1–15. doi: 10.3390/nu8090528

74. Lim CGY, Whitton C, Rebello SA, Van Dam RM. Diet Quality and Lower Refined Grain Consumption are Associated With Less Weight Gain in a Multi-Ethnic Asian Adult Population. *J Nutr* (2021) 151:2372–2382. doi: 10.1093/jn/nxab110

75. Colles S, Singh S, Kohli C, Mithal A. Dietary beliefs and eating patterns influence metabolic health in type 2 diabetes: A clinic-based study in urban North India. *Indian J Endocrinol Metab* (2013) 17:1066. doi: 10.4103/2230-8210.122626

76. Mohan V, Radhika G, Sathya RM, Tamil SR, Ganesan A, Sudha V. Dietary carbohydrates, glycaemic load, food groups and newly detected type 2 diabetes among urban Asian Indian population in Chennai, India (Chennai Urban Rural Epidemiology Study 59). *Br J Nutr* (2009) 102:1498–1506. doi: 10.1017/S0007114509990468

77. Safdar NF, Bertone-Johnson E, Cordeiro L, Jafar TH, Cohen NL. Dietary patterns of Pakistani adults and their associations with sociodemographic, anthropometric and life-style factors. *J Nutr Sci* (2013) 2:1–10. doi: 10.1017/jns.2013.37

78. Abeywickrama HM, Swarna Wimalasiri KM, Koyama Y, Uchiyama M, Shimizu U, Chandrajith R, Nanayakkara N. Assessment of nutritional status and dietary pattern of a rural adult population in dry zone, Sri Lanka. *Int J Environ Res Public Health* (2020) 17:1–14. doi: 10.3390/ijerph17010150

79. Parthasarathy LS, Chiplonkar SA, Khadilkar A V., Khadilkar V V. Dietary modifications to improve micronutrient status of Indian children and adolescents with type 1 diabetes. *Asia Pac J Clin Nutr* (2015) 24:73–82. doi: 10.6133/apjcn.2015.24.1.04

80. Iqbal R, Ajayan K, Bharathi A V., Zhang X, Islam S, Soman CR, Merchant AT. Refinement and validation of an FFQ developed to estimate macro- and micronutrient intakes in a south Indian population. *Public Health Nutr* (2009) 12:12–18. doi: 10.1017/S1368980008001845

81. Mustafa S, Haque CE, Baksi S. Low Daily Intake of Fruits and Vegetables in Rural and Urban Bangladesh : Influence of Socioeconomic and Demographic. *Nutrients* (2021) 13:2808.

82. Ojha SN, Anand A, Sundriyal RC, Arya D. Traditional Dietary Knowledge of a Marginal Hill Community in the Central Himalaya: Implications for Food, Nutrition, and Medicinal Security. *Front Pharmacol* (2022) 12:1–22. doi: 10.3389/fphar.2021.789360

83. Mumu SJ, Merom D, Ali L, Fahey PP, Hossain I, Rahman AKMF, Allman-Farinelli M. Validation of a food frequency questionnaire as a tool for assessing dietary intake in cardiovascular disease research and surveillance in Bangladesh. *Nutr J* (2020) 19:1–16. doi: 10.1186/s12937-020-00563-7

84. Abeywickrama HM, Koyama Y, Uchiyama M, Shimizu U, Iwasa Y, Yamada E, Ohashi K, Mitobe Y. Micronutrient status in Sri Lanka: A review. *Nutrients* (2018) 10:1–20. doi: 10.3390/nu10111583

85. Shalini T, Sivaprasad M, Balakrishna N, Madhavi G, Radhika MS, Kumar BN, Pullakhandam R, Reddy GB. Micronutrient intakes and status assessed by probability approach among the urban adult population of Hyderabad city in South India. *Eur J Nutr* (2019) 58:3147–3159. doi: 10.1007/s00394-018-1859-y

86. Nair S, Bandyopadhyay S. Sodium Intake Pattern in West Indian Population. *Indian J Community Med* (2018)67–71.

87. Sudha V, Radhika G, Sathya RM, Ganesan A, Mohan V. Reproducibility and validity of an interviewer-administered semi-quantitative food frequency questionnaire to assess dietary intake of urban adults in southern India. *Int J Food Sci Nutr* (2006) 57:481–493. doi: 10.1080/09637480600969220

88. Shetty PS. Nutrition transition in India. *Public Health Nutr* (2002) 5:175–182. doi: 10.1079/phn2001291

89. Vaidya R, Vaidya ADB, Sheth J, Jadhav S, Mahale U, Mehta D, Popko J, Badmaev V, Stohs SJ. Vitamin K Insufficiency in the Indian Population: Pilot Observational Epidemiology Study. *JMIR Public Heal Surveill* (2022) 8:1–7. doi: 10.2196/31941

90. Riaz H, Finlayson AE, Bashir S, Hussain S, Mahmood S, Malik F, Godman B. Prevalence of Vitamin D deficiency in Pakistan and implications for the future. *Expert Rev Clin Pharmacol* (2016) 9:329–338. doi: 10.1586/17512433.2016.1122519

91. Fahim O, Shahim S, Shams AN, Muhammadi AF, Djazayery A, Esmaillzadeh A. Double burden of malnutrition in Afghanistan: Secondary analysis of a national survey. *PLoS One* (2023) 18:1–19. doi: 10.1371/journal.pone.0284952

92. Sudo N, Sekiyama M, Ohtsuka R, Maharjan M. Gender differences in “luxury food intake” owing to temporal distribution of eating occasions among adults of Hindu communities in lowland Nepal. *Asia Pac J Clin Nutr* (2009) 18:441–446.

93. Chakraborty R, Bose K, Ulijaszek S. Income Level and Food Intake Patterns among Male Bengalee Slum Dwellers in Kokata, India. *Malays J Nutr* (2009) 15:19–25.

94. Singh M, Kirchengast S. Obesity prevalence and nutritional habits among Indian women: A comparison between Punjabi women living in India and Punjabi migrants in Vienna, Austria. *Anthropol Anzeiger* (2011) 68:239–251. doi: 10.1127/0003-5548/2011/0132

95. Mark HE, Houghton LA, Gibson RS, Monterrosa E, Kraemer K. Estimating dietary micronutrient supply and the prevalence of inadequate intakes from national Food Balance Sheets in the South Asia region. *Asia Pac J Clin Nutr* (2016) 25:368–376. doi: 10.6133/apjcn.2016.25.2.11

96. Chen Y, Ahsan H, Parvez F, Howe GR. Validity of a food-frequency questionnaire for a large prospective cohort study in Bangladesh. *Br J Nutr* (2004) 92:851–859. doi: 10.1079/bjn20041277

97. Bharathi AV, Kurpad AV, Thomas T, Yusuf S, Saraswathi G, Vaz M. Development of food frequency questionnaires and a nutrient database for the Prospective Urban and Rural Epidemiological (PURE) pilot study in South India: methodological issues. *Asia Pac J Clin Nutr* (2008) 17:178–85. http://www.ncbi.nlm.nih.gov/pubmed/18364343

98. Sudo N, Sekiyama M, Maharjan M, Ohtsuka R. Gender differences in dietary intake among adults of Hindu communities in lowland Nepal: Assessment of portion sizes and food consumption frequencies. *Eur J Clin Nutr* (2006) 60:469–477. doi: 10.1038/sj.ejcn.1602339

99. Ryckman T, Beal T, Nordhagen S, Murira Z, Torlesse H. Affordability of nutritious foods for complementary feeding in South Asia. *Nutr Rev* (2021) 79:52–68. doi: 10.1093/nutrit/nuaa139

100. Anjana RM, Pradeepa R, Das AK, Deepa M, Bhansali A, Joshi SR, Joshi PP, Dhandhania VK, Rao P V, Sudha V, et al. Physical activity and inactivity patterns in India – results from the ICMR-INDIAB study. (2014)1–11.

101. Jayawardena R, Jeyakumar DT, Gamage M, Sooriyaarachchi P, Hills AP. Fruit and vegetable consumption among South Asians: A systematic review and meta-analysis. *Diabetes Metab Syndr Clin Res Rev* (2020) 14:1791–1800. doi: 10.1016/j.dsx.2020.09.004

102. Soofi S, Khan GN, Sadiq K, Ariff S, Habib A, Kureishy S, Hussain I, Umer M, Suhag Z, Rizvi A, et al. Prevalence and possible factors associated with anaemia, and Vitamin B 12 and folate deficiencies in women of reproductive age in Pakistan: Analysis of national-level secondary survey data. *BMJ Open* (2017) 7: doi: 10.1136/bmjopen-2017-018007

103. Vijay J, Kumar Patel K. Recommendations to scale up dietary diversity data at household and individual level in India. *Diabetes Metab Syndr Clin Res Rev* (2021) 15:102310. doi: 10.1016/j.dsx.2021.102310

104. Weerasekara PC, Withanachchi CR, Ginigaddara GAS, Ploeger A. Understanding Dietary Diversity, Dietary Practices and Changes in Food Patterns in Marginalised Societies in Sri Lanka. *Foods* (2020) 9: doi: 10.3390/foods9111659

105. Ali Z, Scheelbeek PFD, Sanin KI, Thomas TS, Ahmed T, Prentice AM, Green R. Characteristics of distinct dietary patterns in rural bangladesh: Nutrient adequacy and vulnerability to shocks. *Nutrients* (2021) 13:1–13. doi: 10.3390/nu13062049

106. Lin PID, Bromage S, Mostofa MG, Allen J, Oken E, Kile ML, Christiani DC. Validation of a dish-based semiquantitative food questionnaire in rural Bangladesh. *Nutrients* (2017) 9:1–18. doi: 10.3390/nu9010049

107. Shrestha A, Koju RP, Beresford SAA, Chan KCG, Connell FA, Karmacharya BM, Shrestha P, Fitzpatrick AL. Reproducibility and relative validity of food group intake in a food frequency questionnaire developed for Nepalese diet. *Int J Food Sci Nutr* (2017) 68:605–612. doi: 10.1080/09637486.2016.1268099

108. Waid JL, Sinharoy SS, Ali M, Stormer AE, Thilsted SH, Gabrysch S. Dietary Patterns and Determinants of Changing Diets in Bangladesh from 1985 to 2010. *Curr Dev Nutr* (2019) 3:1–14. doi: 10.1093/cdn/nzy091

109. Sharma M, Kishore A, Roy D, Joshi K. A comparison of the Indian diet with the EAT-Lancet reference diet. *BMC Public Health* (2020) 20:1–13. doi: 10.1186/s12889-020-08951-8

110. Al Hasan SM, Saulam J, Kanda K, Hirao T. Temporal trends in apparent food consumption in bangladesh: Ajoinpoint regression analysis of fao’s food balance sheet data from 1961 to 2013. *Nutrients* (2019) 11: doi: 10.3390/nu11081864

111. Al Hasan SM, Saulam J, Kanda K, Murakami A, Yamadori Y, Mashima Y, Ngatu NR, Hirao T. Temporal trends in apparent energy and macronutrient intakes in the diet in bangladesh: A joinpoint regression analysis of the fao’s food balance sheet data from 1961 to 2017. *Nutrients* (2020) 12:1–18. doi: 10.3390/nu12082319

112. Anand S, Shivashankar R, Kondal D, Garg V, Khandelwal S, Gupta R, Krishnan A, Amarchand R, Poulter N, Reddy KS, et al. Potassium Intake in India: Opportunity for Mitigating Risks of High-Sodium Diets. *Am J Prev Med* (2020) 58:302–312. doi: 10.1016/j.amepre.2019.09.017

113. Tak M, Law C, Green R, Shankar B, Cornelsen L. Processed foods purchase profiles in urban India in 2013 and 2016: a cluster and multivariate analysis. *BMJ Open* (2022) 12:1–11. doi: 10.1136/bmjopen-2022-062254

114. Johnson C, Mohan S, Rogers K, Shivashankar R, Thout SR, Gupta P, He FJ, MacGregor GA, Webster J, Krishnan A, et al. Mean dietary salt intake in urban and rural areas in India: A population survey of 1395 persons. *J Am Heart Assoc* (2017) 6: doi: 10.1161/JAHA.116.004547

115. Neupane D, Rijal A, Henry ME, Kallestrup P, Koirala B, Mclachlan CS, Ghimire K, Zhao D, Sharma S, Pokharel Y, et al. Mean dietary salt intake in Nepal: A population survey with 24-hour urine collections. *J Clin Hypertens* (2020) 22:273–279. doi: 10.1111/jch.13813

116. Sowmya N, Lakshmipriya N, Arumugam K, Venkatachalam S, Vijayalakshmi P, Ruchi V, Geetha G, Anjana RM, Mohan V, Krishnaswamy K, et al. Comparison of dietary profile of a rural south Indian population with the current dietary recommendations for prevention of non-communicable diseases (CURES 147). *Indian J Med Res* (2016) 144:112–119. doi: 10.4103/0971-5916.193297

117. Siddiqee MH, Bhattacharjee B, Siddiqi UR, MeshbahurRahman M. High prevalence of vitamin D deficiency among the South Asian adults: a systematic review and meta-analysis. *BMC Public Health* (2021) 21:1–18. doi: 10.1186/s12889-021-11888-1

118. Smith MR, DeFries R, Chhatre A, Ghosh-Jerath S, Myers SS. Inadequate Zinc Intake in India: Past, Present, and Future. *Food Nutr Bull* (2019) 40:26–40. doi: 10.1177/0379572118825176

119. Jayawardena R, Byrne NM, Soares MJ, Katulanda P, Hills AP. Validity of a food frequency questionnaire to assess nutritional intake among Sri Lankan adults. *Springerplus* (2016) 5:1–6. doi: 10.1186/s40064-016-1837-x

120. Jayawardena R, Thennakoon S, Byrne N, Soares M, Katulanda P, Hills A. Energy and nutrient intakes among Sri Lankan adults. *Int Arch Med* (2014) 7:1–11. doi: 10.1186/1755-7682-7-34

121. Sivaprasad M, Shalini T, Balakrishna N, Sudarshan M, Lopamudra P, Suryanarayana P, Arlappa N, Ravikumar BP, Radhika MS, Reddy GB. Status of Vitamin B12 and Folate among the Urban Adult Population in South India. *Ann Nutr Metab* (2016) 68:94–102. doi: 10.1159/000442677

122. Mahajan R, Malik M, Bharathi A V., Lakshmi PVM, Patro BK, Rana SK, Kumar R. Reproducibility and validity of a quantitative food frequency questionnaire in an urban and rural area of northern India. *Natl Med J India* (2013) 26:266–272.

123. Coleman FM, Ahmed AU, Quisumbing AR, Roy S, Hoddinott J. Diets of Men and Women in Rural Bangladesh Are Equitable but Suboptimal. *Curr Dev Nutr* (2023) 7:100107. doi: 10.1016/j.cdnut.2023.100107

124. Akhtar S, Ismail T, Atukorala S, Arlappa N. Micronutrient deficiencies in South Asia - Current status and strategies. *Trends Food Sci Technol* (2013) 31:55–62. doi: 10.1016/j.tifs.2013.02.005

125. Sudha V, Anjana RM, Vijayalakshmi P, Lakshmipriya N, Kalpana N, Gayathri R, Priyadarshini RD, Malini HM, Anitha C, Deepa M, et al. Reproducibility and construct validity of a food frequency questionnaire for assessing dietary intake in rural and urban Asian Indian adults. *Asia Pac J Clin Nutr* (2020) 29:192–204. doi: 10.6133/apjcn.202003_29(1).0025

126. Shridhar K, Dhillon PK, Bowen L, Kinra S, Bharathi AV. Nutritional profile of Indian vegetarian diets – the Indian Migration Study (IMS). *Nutr J* (2014) 13:

127. Mazumder DNG, Deb D, Biswas A, Saha C, Nandy A, Ganguly B, Ghose A, Bhattacharya K, Majumdar KK. Evaluation of dietary arsenic exposure and its biomarkers: A case study of West Bengal, India. *J Environ Sci Heal - Part A Toxic/Hazardous Subst Environ Eng* (2013) 48:896–904. doi: 10.1080/10934529.2013.761495

128. Radhika G, Sathya RM, Ganesan A, Saroja R, Vijayalakshmi P, Sudha V, Mohan V. Dietary profile of urban adult population in South India in the context of chronic disease epidemiology (CURES - 68). *Public Health Nutr* (2010) 14:591–598. doi: 10.1017/S136898001000203X

129. Anjana RM, Sudha V, Nair DH, Lakshmipriya N, Deepa M, Pradeepa R, Shanthirani CS, Subhashini S, Malik V, Unnikrishnan R, et al. Diabetes in Asian Indians-How much is preventable? Ten-year follow-up of the Chennai Urban Rural Epidemiology Study (CURES-142). *Diabetes Res Clin Pract* (2015) 109:253–261. doi: 10.1016/j.diabres.2015.05.039

130. Shabnam N, Ashraf MA, Laar RA, Ashraf R. Increased Household Income Improves Nutrient Consumption in Pakistan: A Cross-Sectional Study. *Front Nutr* (2021) 8:1–11. doi: 10.3389/fnut.2021.672754

131. Zavos HMS, Riddleston L, Jayaweera K, Dissanayake L, Jabir S. Frequency of Consumption of Food Groups and Cardio ‑ Metabolic Risk Factors : A Genetically Informative Twin Study in Sri Lanka. *Behav Genet* (2024) 54:73–85. doi: 10.1007/s10519-023-10165-8

132. Harris-Fry H, Shrestha N, Costello A, Saville NM. Determinants of intra-household food allocation between adults in South Asia - A systematic review. *Int J Equity Health* (2018) 16:1–21. doi: 10.1186/s12939-017-0603-1

133. Sathyamala C. Meat-eating in India: Whose food, whose politics, and whose rights? *Policy Futur Educ* (2019) 17:878–891. doi: 10.1177/1478210318780553

134. Khan M, Damalas CA. Farmers’ willingness to pay for less health risks by pesticide use: A case study from the cotton belt of Punjab, Pakistan. *Sci Total Environ* (2015) 530–531:297–303. doi: 10.1016/j.scitotenv.2015.05.110

135. Upadhyay PR. Curbing Malnutrition and Micronutrient Defi ciencies in India… Food Based Strategies at the Centre of a Holistic Approach. *Int J Med Public Heal* (2011) 1:24–27. doi: 10.5530/ijmedph.4.2011.6

136. Sraboni E, Quisumbing A. Women’s empowerment in agriculture and dietary quality across the life course: Evidence from Bangladesh. *Food Policy* (2018) 81:21–36. doi: 10.1016/j.foodpol.2018.09.001

137. Srinivasan JT, Reddy VR. Impact of irrigation water quality on human health: A case study in India. *Ecol Econ* (2009) 68:2800–2807. doi: 10.1016/j.ecolecon.2009.04.019

138. Rao T, Pingali P. The role of agriculture in women’s nutrition: Empirical evidence from India. *PLoS One* (2018) 13:1–17. doi: 10.1371/journal.pone.0201115

139. Rahman KMM, Islam MA. Nutrition-sensitive agriculture in Bangladesh: a review. *Food Secur* (2014) 6:671–683. doi: 10.1007/s12571-014-0380-2

140. Jha R, Gaiha R, Sharma A. Calorie and Micronutrient Deprivation and Poverty Nutrition Traps in Rural India. *World Dev* (2009) 37:982–991. doi: 10.1016/j.worlddev.2008.09.008

141. Nithya DJ, Bhavani R V. Dietary diversity and its relationship with nutritional status among adolescents and adults in rural India. *J Biosoc Sci* (2018) 50:397–413. doi: 10.1017/S0021932017000463

142. Nahar N, Uddin M, Sarkar RA, Gurley ES, Uddin Khan MS, Hossain MJ, Sultana R, Luby SP. Exploring pig raising in Bangladesh: implications for public health interventions. *Vet Ital* (2013) 49:7–17. http://www.ncbi.nlm.nih.gov/pubmed/23564585

143. Balagamwala M, Gazdar H, Mallah HB. Synergy or Trade-Off between Agricultural Growth and Nutrition Women’s Work and Care. *The Pakistan Development Review*. Pakistan Institute of Development Economics (2015)

144. Mulmi P, Masters WA, Ghosh S, Namirembe G, Rajbhandary R, Manohar S, Shrestha B, West KP, Webb P. Household food production is positively associated with dietary diversity and intake of nutrient-dense foods for older preschool children in poorer families: Results from a nationally-representative survey in Nepal. *PLoS One* (2017) 12:1–23. doi: 10.1371/journal.pone.0186765

145. Mulmi P, Block SA, Shively GE, Masters WA. Climatic conditions and child height: Sex-specific vulnerability and the protective effects of sanitation and food markets in Nepal. *Econ Hum Biol* (2016) 23:63–75. doi: 10.1016/j.ehb.2016.07.002

146. Aich M, Mahzebin M, Subarna NF, Hassan A. A study on socio-economic condition and nutritional profile of women worker’s in shrimp and agriculture sectors in selected two districts of Bangladesh. *Int Res J Soc Sci* (2014) 3:15–21. http://www.isca.in/IJSS/Archive/v3/i3/4.ISCA-IRJSS-2014-08.pdf

147. Mohsena M, Hossain M, Chakraborty B, Bayes A, Rahman ANM. Fragile Environment, Seasonality and Maternal and Childhood Undernutrition in Bangladesh. *J Biosoc Sci* (2018) 50:579–603. doi: 10.1017/S002193201700044X

148. Misra M. Moving away from technocratic framing: agroecology and food sovereignty as possible alternatives to alleviate rural malnutrition in Bangladesh. *Agric Human Values* (2018) 35:473–487. doi: 10.1007/s10460-017-9843-3

149. Malapit HJL, Kadiyala S, Quisumbing AR, Cunningham K, Tyagi P. Women’s Empowerment Mitigates the Negative Effects of Low Production Diversity on Maternal and Child Nutrition in Nepal. *J Dev Stud* (2015) 51:1097–1123. doi: 10.1080/00220388.2015.1018904

150. Naz M, Khan IA, Shahbaz B. Role of rural women in agriculture and household food security in Faisalabad district. *Pakistan J Agric Sci* (2014) 51:757–761.

151. Komatsu H, Malapit HJL, Theis S. Does women’s time in domestic work and agriculture affect women’s and children’s dietary diversity? Evidence from Bangladesh, Nepal, Cambodia, Ghana, and Mozambique. *Food Policy* (2018) 79:256–270. doi: 10.1016/j.foodpol.2018.07.002

152. Joshi P, Sharma JP, Sharma N, Singh BK, Ahmad N, Gills R. Health and nutrition status of farm women in rural India: Case of Indo-Gangetic plains. *Indian J Agric Sci* (2018) 88:978–984. doi: 10.56093/ijas.v88i6.80657

153. Kadiyala S, Harris J, Headey D, Yosef S, Gillespie S. Agriculture and nutrition in India: Mapping evidence to pathways. *Ann N Y Acad Sci* (2014) 1331:43–56. doi: 10.1111/nyas.12477

154. Gulati A, Ganesh-Kumar A, Shreedhar G, Nandakumar T. Agriculture and malnutrition in India. *Food Nutr Bull* (2012) 33:

155. Gartaula H, Patel K, Johnson D, Devkota R, Khadka K, Chaudhary P. From food security to food wellbeing: examining food security through the lens of food wellbeing in Nepal’s rapidly changing agrarian landscape. *Agric Human Values* (2017) 34:573–589. doi: 10.1007/s10460-016-9740-1

156. Flores-Martinez A, Zanello G, Shankar B, Poole N. Reducing anemia prevalence in Afghanistan: Socioeconomic correlates and the particular role of agricultural assets. *PLoS One* (2016) 11:1–22. doi: 10.1371/journal.pone.0156878

157. Cunningham K, Ploubidis GB, Menon P, Ruel M, Kadiyala S, Uauy R, Ferguson E. Women’s empowerment in agriculture and child nutritional status in rural Nepal. *Public Health Nutr* (2015) 18:3134–3145. doi: 10.1017/S1368980015000683

158. Broaddus-Shea ET, Thorne-Lyman AL, Manohar S, Nonyane BAS, Winch PJ, West KP. Seasonality of consumption of nonstaple nutritious foods among young children from Nepal’s 3 agroecological zones. *Curr Dev Nutr* (2018) 2:nzy058. doi: 10.1093/cdn/nzy058

159. Vijaya Bhaskar A V., Nithya DJ, Raju S, Bhavani R V. Establishing integrated agriculture-nutrition programmes to diversify household food and diets in rural India. *Food Secur* (2017) 9:981–999. doi: 10.1007/s12571-017-0721-z
