# Supplementary material for: South Asia-specific adaptation of Mediterranean diet principles: a mixed-methods review for practical and sustainable dietary habits
Source: Front Nutr. 2025 Dec 23;12:1719686. doi: 10.3389/fnut.2025.1719686 (PMC12786337; doi:10.3389/fnut.2025.1719686)
Supplement: Supplementary file 6 [file Table_6.DOCX]

**Supplementary file 7. Sustainable and MD-tailored dietary recommendations for South Asia**

At the base of the pyramids, we incorporated a set of foundational healthy habits that should be consistently followed, serving as essential complementary behaviours to a MD-oriented South Asian dietary pattern:

- Conviviality, recognized as a social and cultural value.
- Respect for food seasonality, to reduce the consumption of processed foods and promote the use of fresh, locally sourced ingredients.
- Physical activity, including both aerobic and strength training.
- Adequate rest, as an integral component of a healthy lifestyle.
- Outdoor activities, as a source of sun exposure, physical movement, and social interaction.
- Mindfulness and meditation, to reduce stress and support mental well-being.

Additionally, access to safe and clean water is emphasized not only as a fundamental human right but also as a key aspect of healthy nutrition. Water intake can be complemented with herbal infusions, tea, and low-salt, low-fat broths. While individual hydration needs vary, a general recommended intake ranges between 1.5 and 3 litres per day.

**Vegetarian pyramid**

Each day, two or three main meals should always include one or two portions of:

- Cereals or starchy tubers: These serve as the primary sources of energy and carbohydrates and are generally affordable. Whole grains should be consumed frequently, while refined rice, wheat, and starchy roots can be included once a day, provided that the overall dietary recommendations are followed.
- Vegetables: These provide vitamins, minerals, fiber, and antioxidants while contributing a minimal caloric load. Vegetables in this category exclude starchy tubers, fruits, spices, and legumes. Compared to non-vegetarians, their consumption can be slightly lower given the higher intake of legumes
- Fruits: Like vegetables, fruits supply vitamins, minerals, fiber, and antioxidants, but their higher fructose content results in a slightly greater energy contribution, suggesting a more moderate intake. Fresh fruits can also serve as snacks or be incorporated into healthy desserts.

***Each main meal should also include one portion of:***

- Legumes and pulses: These constitute the primary protein source for vegetarians, including beans (rajma), soy, lentils (dal), peas, and chickpeas. They can be consumed fresh or processed into various traditional preparations, often in combination with grains.
- Spices: A fundamental component of South Asian cuisine, spices enhance flavour while reducing the need for added salt. They also contribute beneficial micronutrients and antioxidants, supporting a balanced and healthy diet.
- Seasoning and cooking fats: These represent the principal source of dietary fat. The selection of fats should consider not only taste but also their chemical composition, health benefits, and geographical availability. A balanced use of rapeseed/canola, sesame, and sunflower oil is recommended for raw dressings, whereas ghee, coconut ghee, and soy or peanut oil are more suitable for cooking (excluding frying). Mustard oil is suitable for both purposes, but a modest intake is recommended due to the high erucic acid content. The use of vanaspati should be avoided.

***Additionally, each day, it is recommended to consume one or two portions of:***

- Milk and dairy products: These provide complementary proteins and essential micronutrients such as calcium, iodine, omega-3 polyunsaturated fatty acids, vitamin B12, and vitamin B2, which may be less abundant in other vegetarian food groups. Due to significant variations in fat content, low-fat options such as milk, yogurt (dahi, curd), and low-fat paneer should be preferred.
- Nuts and seeds: These serve as valuable sources of vitamins, minerals, fiber, and healthy fats. Their intake should be balanced based on overall dietary fat consumption. Specific emphasis is placed on nuts and seeds rich in omega-3 polyunsaturated fatty acids, such as flaxseeds, basil (sabja) seeds, white mustard seeds, and walnuts. Their placement higher in the pyramid compared to oils and ghee is primarily due to their relatively higher cost.

***Each week, two to four portions should be included in the diet from the following food groups:***

- Eggs (not always consumed by vegetarians in South Asia): They represent a high-quality protein source that provides essential micronutrients such as vitamin B12 and choline, which are particularly relevant in vegetarian diets. Their consumption should be considered as an alternative to dairy when possible and culturally acceptable.
- Healthy homemade sweets and beverages: These include minimally processed traditional preparations made with natural ingredients, such as legume-based sweets and snacks, fruit-based desserts, and low-sugar lassi. This category does not include fried foods.

***Each week, less than two portions in total should come from the following food groups:***

- Ultra-processed foods: These include packaged snacks, ready-to-eat meals, sugar-sweetened beverages and processed vegetarian meat substitutes, which often contain high levels of sodium, sugar, unhealthy fats, and additives.
- Fried foods: Foods prepared using deep frying should be limited due to their high trans-fat and calorie content.

**Non-vegetarian pyramid**

Each day, two or three main meals should always include one or two portions of:

- Cereals or starchy tubers: As for the vegetarians, but whole grains consumption should be higher due to the lower intake of legumes and pulses.
- Vegetables: As for vegetarians, but in slightly higher doses to counteract the lower fiber and antioxidant intake.
- Fruits: As for vegetarians.

***Each main meal should also include one portion of:***

- Spices: A defining element of South Asian cuisine, spices not only enhance flavor but also provide beneficial compounds such as curcumin (turmeric), piperine (black pepper), and capsaicin (chili peppers). Their use helps reduce the need for added salt while contributing to a balanced diet.
- Seasoning and cooking fats: A combination of sesame oil, rapeseed/canola oil, and sunflower oil is recommended for raw use, while ghee, coconut ghee, and peanut or soy oil are more suitable for cooking. Mustard oil is suitable for both purposes, but a modest intake is recommended due to the high erucic acid content. Vanaspati and hydrogenated fats should be avoided due to their trans-fat content.

***Additionally, once or twice per day, it is recommended to consume one or two portions of:***

- Milk and dairy products: Their consumption should be balanced with the intake of flesh foods to not exceed with animal protein and fat.
- Legumes and pulses: Their consumption should be slightly reduced compared to vegetarians since non-vegetarians have a wider choice of protein sources, including animal-based options. However, they should remain an important component of non-vegetarian diets due to their health benefits and sustainability.
- Nuts and seeds: As for vegetarians, but less emphasis on Ω3-rich seeds if fish intake is in line with recommendations.

***Each week, two to four portions should be included in the diet from the following food groups:***

- Eggs: Balanced with intake of meat, fish, and dairy to avoid excessive animal protein. When possible, opt for boiled or poached preparations over deep-fried versions.
- Fish and seafood: This category provides crucial nutrients such as high-quality protein, essential fatty acids, iodine, selenium and zinc. A balanced combination of seafood, including shellfish, lean and fatty fish is recommended.
- White meat: Chicken, other poultry sources and rabbit provide lean proteins rich in iron, zinc and B vitamins. Traditional preparations and curries without excessive oil are preferable to fried options.
- Healthy homemade sweets and beverages, excluding fried foods: Non-vegetarians should limit such sweets to festive occasions, use lighter versions with reduced sugar and fat, and avoid pairing them with heavy meals.

***Each week, a maximum of one portion should come from:***

- Red meat: This category is including mutton, goat, lamb, beef and pork meats. While a rich source of iron, zinc, and vitamin B12, red meat consumption should be limited to lean cuts to minimize saturated fat intake and other negative effects on health.
- Ultra-processed foods, fried foods, processed meat and sugar-sweetened beverages, which should be strictly limited.

|  | Vegetarians | Non vegetarians |
| --- | --- | --- |
| Each main meal (one or two portions) |  |  |
| Cereals and starchy tubers | Primary sources of energy and carbohydrates and highly affordable. Whole grains should be  consumed frequently, while refined rice, wheat and starchy roots can be included once a day, provided that the overall dietary recommendations are followed. | As for the vegetarians, but whole grains  consumption should be higher due to the lower intake of legumes |
| Vegetables | Provide vitamins, minerals, fiber, and antioxidants while contributing a minimal caloric load. Vegetables  in this category exclude starchy tubers, fruits, spices, and legumes. Compared to non-vegetarians, their consumption can be slightly lower given the higher intake of legumes | As for vegetarians, but in slightly higher doses to counteract the lower fibre and antioxidant intake. |
| Fruits | Like vegetables, fruits supply vitamins, minerals, fiber, and antioxidants, but their higher fructose content results in a  slightly greater energy contribution, suggesting a more moderate intake. Fresh fruits can also serve as snacks or be  incorporated into healthy desserts. | As for vegetarians. |
| Legumes and pulses | These constitute the primary protein source for vegetarians, including beans (rajma), soy, lentils (dal),  peas, and chickpeas. They can be consumed fresh or processed into various traditional preparations, often in combination  with grains. | Their consumption should be slightly reduced compared to vegetarians since non-vegetarians have a  wider choice of protein sources, including animal-based options. However, they should remain an important component of  non-vegetarian diets due to their health benefits and sustainability. |
| Spices | A fundamental component of South Asian cuisine, spices enhance flavour while reducing the need for added salt.  They also contribute beneficial micronutrients and antioxidants, supporting a balanced and healthy diet. | A defining element of South Asian cuisine, spices not only enhance flavor but also provide beneficial compounds  such as curcumin (turmeric), piperine (black pepper), and capsaicin (chili peppers). Their use helps reduce the need for  added salt while contributing to a balanced diet. |
| Seasoning and cooking fats | These represent the principal source of dietary fat. The selection of fats should consider not  only taste but also their chemical composition, health benefits, and geographical availability. A balanced use of rapeseed/canola, sesame, and sunflower oil is recommended for raw dressings, whereas ghee, coconut ghee, and soy or peanut oils are more suitable for cooking (excluding frying). Mustard oil is suitable for both purposes, but a modest intake is recommended due to the high erucic acid content. The use of vanaspati should be avoided. Prefer non-refined and rich in Ω3 oils | As for vegetarians, but less emphasis on Ω3-rich oils if fish intake is in line with reccomendations |
| Additionally, each day, it is recommended to consume (one or two portions) |  |  |
| Milk and dairy products | These provide complementary proteins and essential micronutrients such as calcium, iodine,  omega-3 polyunsaturated fatty acids, vitamin B12, and vitamin B2, which may be less abundant in other vegetarian food  groups. Due to significant variations in fat content, low-fat options such as milk, yogurt (dahi, curd), and low-fat paneer  should be preferred. | Their consumption should be balanced with the intake of flesh foods to not exceed with animal  protein and fat. |
| Nuts and seeds | These serve as valuable sources of vitamins, minerals, fiber, and healthy fats. Their intake should be  balanced based on overall dietary fat consumption. Specific emphasis is placed on nuts and seeds rich in omega-3 polyunsaturated fatty acids, such as flaxseeds, basil (sabja) seeds, white mustard seeds, and walnuts. Their placement higher in the pyramid compared to oils and ghee is primarily due to their relatively higher cost. | As for vegetarians, but less emphasis on Ω3-rich seeds if fish intake is in line with recommendations. |
| Each week- (two to four portions) |  |  |
| Eggs (not always consumed by vegetarians in South Asia) | They represent a high-quality protein source that provides  essential micronutrients such as vitamin B12 and choline, which are particularly relevant in vegetarian diets. Their  consumption should be considered as an alternative to dairy when possible and culturally acceptable. | Eggs should be balanced with intake of meat, fish, and dairy to avoid excessive animal protein. When possible, opt for boiled or poached preparations over deep-fried versions. |
| Healthy homemade sweets and beverages | These include minimally processed traditional preparations made with natural  ingredients, such as legume-based sweets and snacks, fruit-based desserts, and low-sugar lassi. This category does not  include fried foods. | Non-vegetarians should limit such sweets to festive occasions, use lighter versions with reduced sugar and fat, and avoid pairing them with heavy meals. |
| Each week- | | |
|  | (less than two portions) | (one portion or less) |
| Ultra-processed foods | These include packaged snacks, ready-to-eat meals, sugar-sweetened beverages and processed vegetarian meat substitutes,which often containhigh levels of sodium, sugar, unhealthy fats, and additives. | Should be strictly limited. Includes packaged snacks, ready-to-eat meals, processed meats (e.g. Fries, nuggets), and sugary beverages. These are typically high in unhealthy fats, sodium, and preservatives, which contribute to cardiovascular and metabolic risks. Preference should be given to fresh, home-cooked meals using whole ingredients. |
| Fried foods | Foods prepared using deep frying should be limited due to their high trans-fat and calorie content. | Should be strictly limited. Compared to vegetarian fried items, non-vegetarian fried foods (e.g., fried chicken, meat cutlets) often have higher saturated fat and cholesterol content, increasing cardiometabolic risk. Moreover, when combined with processed meats, the health burden is amplified. Therefore, non-vegetarians should rely more heavily on grilling, baking, or steaming as preferred cooking methods |
