# Supplementary material for: South Asia-specific adaptation of Mediterranean diet principles: a mixed-methods review for practical and sustainable dietary habits
Source: Front Nutr. 2025 Dec 23;12:1719686. doi: 10.3389/fnut.2025.1719686 (PMC12786337; doi:10.3389/fnut.2025.1719686)
Supplement: Supplementary file 7 [file Table_7.DOCX]

**Supplementary File 5.**

**Summary of South Asian vegetarian diet typical food and recipes**

| Food Group | Raw Ingredients | Main Recipes | Raw Properties | Strengths and Weaknesses | Possible Improvements | References |
| --- | --- | --- | --- | --- | --- | --- |
| Rice | Basmati Rice, Jasmine Rice, Brown Rice, Red Rice, Black Rice | **Fermented Rice**  Dosa/Panta Bhat/Idli/Dhokla/Uttapam/Appam/Paniyaram/Bubur Cha Cha/Apong  **Flavored Rice**  Biryani/Nasi Lemak/Nasi Goreng/Pulao/Tahiri/Lamprais/Kiribath/Lemon Rice/Tamarind Rice, Tehari/Zafrani Pulao  **Flatbreads**  Akki roti  **Rice Soups**  Khichuri/Khichdi, Borhani/Congee  **Side Dishes**  Sambhar Rice/Curd Rice/Coconut Rice/Hoppers/Appam, Pittu/Puttu  **Fried Rice**  Sel Roti  **Sweet Rice**  Ricepudding/Kheer/Zarda/Lamprais/Kiribath/Yomari  **Puffed Rice**  Bhel Puri, Jhalmuri  **Rice Cakes**  Pitha | High in carbohydrates, low in fat, moderate in protein. Rich in B vitamins (B1, B3, B9). Contains iron, manganese, selenium. Fiber content varies with processing; whole rice retains more fiber and micronutrients than white rice. Whole ricehashigherantioxidantvalue. | **Strengths**  Versatile and staple in South Asian diets. Provides energy and sustenance. Fermented rice offers probiotics. **Weaknesses**  Refined rice has high glycemic index and low fiber. Often consumed with minimal vegetables or proteins. High oil and sugar content in some recipes. | Increase consumption of whole grains like brown, red, and black rice. Enrich meals with vegetables and legumes. Reduce added oils and sugars. | (1–3) |
| Wheat | Whole Wheat, Sharbati Wheat, Semolina, All-Purpose Flour | **Whole Wheat Flour (Atta) and Sharbati Wheat Flour:** Roti/Chapati/Paratha/Phulka/Punjabi Aloo Paratha/Atta Dosa/Missi Roti/Onion Paratha/Pav, Bhatura  **Semolina**  Idli/Dosa/Upma/Momos, Sheera  **All-Purpose Flour**  Cakes/Pastries/Cookies/Biscuits/Samosas/Appam/Gulgule/Nankhatai, Mawa cake  **Soups**  Wheat flour soup  **Flatbreads**  Naan, Kulcha | High in carbohydrates, protein, B vitamins (B1, B2, B3, B6, B9), and minerals (iron, magnesium, phosphorus). Whole wheat is rich in fiber. All-purpose flour has lower nutrient content due to refining. | **Strengths**  High nutrient density in whole wheat. Good source of energy and protein. Versatile in many recipes.  **Weaknesses**  Refined wheat has high glycemic index, lower fiber, and nutrient loss. High consumption of refined wheat products. | Increase consumption of whole wheat products. Use alternative grains like millet and sorghum. Reduce portions of refined wheat products. Promote biofortified wheat with higher zinc content. | (4,5) |
| Other grains | Jowar (Sorghum), Bajra (Pearl Millet), Ragi (Finger Millet), Maize | **Jowar**: Jowar Roti/Bhakri  **Bajra**: Bajra Roti, Bajra Khichdi, KambuKoozh  **Ragi**: Ragi Dosa/Ragi Mudde/Ragi Malt  **Maize**: Makki Roti/Chakki Roti | High in carbohydrates, fiber, protein, and essential minerals like calcium, iron, magnesium. Most are gluten-free | **Strengths**  Nutrient-dense and suitable for gluten-sensitive individuals. High in fibers. Promote microbiota diversity and quality.  **Weaknesses**  Can be less palatable compared to refined grains.  Presence of anti-nutrients in whole grains.  Shorter shelf life and baking quality compared to refined flours. Maize is naturally low in fiber and has high glycemic index. | Promote traditional recipes using millets and other grains, which can also be fortified for additional nutrient value. Encourage variety in grain consumption. | (6–10) |
| Tubers and starchy roots | Potato, Sweet Potato, Taro (Arbi), Yam/Elephant foot Yam (Orr),Cassava, Lotus Root, | **Main Courses**  Aloo Gobi, Sweet Potato Curry, Taro Curry/Kcamal Kakdi Curry/Orr Curry ,Chatpate, Chotpoti  **Side Dishes**  Aloo Paratha,  Sweet Potato fry/Cassava fry/Orr Fry, Arbi Masala, Kamal Kakdi Ki Subzi,  **Snacks**  Potato Pakoras, Sweet Potato Chaat, Taro Chips, Pani Puri/Golgappa/Aloo Tikki, Samosa | High in carbohydrates, fiber, vitamins (C, B6), minerals (potassium, magnesium, calcium). | **Strengths**  High energy content, rich in vitamins and minerals.  **Weaknesses**  High glycemic index, potential for high calorie content in fried preparations. | Promote the consumption of baked or boiled tubers instead of frying. Encourage the use of sweet potatoes and taro for their higher nutrient content compared to regular potatoes. Educate on portion control to manage calorie intake. Consider the use of spices and herbs to enhance flavor without adding excessive calories or salt. Promote the inclusion of tubers in combination with other vegetables for balanced meals. Encourage the use of tubers in soups and stews for a nutritious addition. | (34–36) |
| Fruits | Mango, Banana, Papaya, Pomegranate, Guava, Pineapple, Jackfruit, Apple, Grapes, Oranges, Watermelon, Melon, Chikoo, Lychee | **Local Fruit Juices**  Aam Ras/Sugarcane Juice/Nariyal Pani/Anar ka Ras/Guava Juice/Pineapple Juice/Amla Juice/Pomegranate Juice, Lemon Sherbet  **Traditional Desserts**  Mango Lassi, Banana Fritters, Papaya Halwa, Pomegranate Raita | Rich in vitamins (A, C, E, K), minerals (potassium, magnesium, calcium), fiber, antioxidants (polyphenols, flavonoids). | **Strengths**  High in vitamins and minerals, rich in antioxidants, hydrating properties.  **Weaknesses:**  Variable nutrient content depending on ripeness, potential for high sugar content in some fruits, pesticide residues. | Promote the consumption of a variety of fruits to ensure a broad spectrum of nutrients. Encourage the use of organic and locally sourced fruits to reduce pesticide exposure. Educate on the benefits of consuming whole fruits instead of fruit juices to retain fiber content. Promote the inclusion of fruits in every meal for balanced nutrition. Encourage home gardening to increase access to fresh fruits. Consider the use of biofortified fruits to enhance nutrient content. Encourage the use of fruits in combination with nuts and seeds for balanced snacks. | (14,18,30–33) |
| Vegetables | Okra, Drumstick, Drumstick Leaves, Eggplant (Baingan), Spinach, Bitter Gourd, Bottle Gourd, Carrot, Radish, Tomato, Cauliflower, Cabbage, Chayote, Pumpkin,  Bamboo shoots | **Main Courses** Vegetable Curry/Aloo Gobi/Baingan Bharta/Palak Paneer, Undhiyu/Eromba  **Side dishes** Achar/Raita/Aloo Paratha/Bhindi Masala, Chana Chaat, Vegetable Pakoras  **Salads**  Mixed fresh salads  **Pickles**  Pachranga Achar/Gongura pickle/Lal Mirch ka Bharwa Achar/DalleKhursani Achar/Pika pila  **Snacks**  Bhaji /Pakoras, Chana Chaat  **Fermented vegetables dishes**  Gundruk, Sinki, Masyaura | Rich in vitamins (A, C, K, folate), minerals (potassium, magnesium, calcium), fiber, antioxidants (polyphenols, flavonoids). Low on calories, proteins, fats. | **Strengths**  High nutrient density, rich in vitamins and minerals, fiber content supports digestive health.  **Weaknesses**  Potential for pesticide residue; variable nutrient content depending on preparation methods; overcooking can reduce nutrient content; presence of anti-nutrients in some cases | Promote the consumption of a variety of vegetables to ensure a broad spectrum of nutrients. Encourage the use of organic and locally sourced vegetables to reduce pesticide exposure. Educate on the benefits of minimal cooking methods to preserve nutrients. Promote the inclusion of vegetables in every meal for balanced nutrition. Encourage home gardening and foraging to increase access to fresh vegetables. Consider the use of biofortified vegetables to enhance nutrient content. | (14,18,30–33) |
| Herb/Spice | Black Pepper, Chili Pepper, Turmeric, Ginger, Garlic, Clove, Cinnamon, Cardamom, Saffron, MustardSeeds, Fennel, Coriander, Nutmeg, Fenugreek | **Curries:** Butter chicken, Masala curry, Korma  **Rice Dishes:** Biryani, Pulao  **Condiments:** Mustard, Chutneys, Hot sauces  **Soups:** Tom yum, Mulligatawny  **Teas:** Chai, Herbal infusions  **BakedGoods:** Spiced bread, Cookies, Cakes  **Beverages:** Golden milk, Saffron tea | Rich in bioactive compounds, essential oils, polyphenols, flavonoids, and minerals | **Strengths**  Antioxidant, anti-inflammatory, antimicrobial, digestive aid, metabolism support, cardiovascular health, and immunity effects.  **Weaknesses**  Pepper, chili and mustard may cause irritation in sensitive people.  Potential adulteration with heavy metals or presence of micotoxins | Encourage balanced use, avoid excessive consumption of strong spices, promote fresh over processed forms, and enhanced absorption (like black pepper with turmeric) | (43,44) |
| Oils and coking fats | Mustard oil, Coconut oil, Ghee, Coconut ghee, Sesame oil, Peanut oil, Palm oil, Sunflower oil,Soy bean oil, Rapeseed oil,  Vanaspati | **Tempering**  Dal Tadka  **Deep Frying** Samosa/Pakoras Shallow frying  Aloo Paratha  **Roasting**  Baingan Bharta  **Cooking in Oil-Based Gravies**  Rogan Josh, Chicken Karahi  **Seasoning Salads** Kachumber | High in fats (saturated, monounsaturated, polyunsaturated), vitamins (E), and antioxidants. Mustard oil: omega-3 and omega-6 fatty acids, vitamin E. Coconut oil: saturated fats, lauric acid, medium chain triglycerides. Ghee: saturated fats, vitamins (A, D, E, K). Sesame oil: unsaturated fats, antioxidants, vitamin E. Peanut oil: monounsaturated fats, vitamin E. Palm oil: saturated fats, vitamin E, carotenes.  Vanaspati: saturated fats and high amount of trans fats | **Strengths**  Rich in essential fatty acids, vitamin E, and antioxidants. Versatile in cooking methods. **Weaknesses**  High calorie and fat content, potential health risks with excessive consumption, environmental impact of palm oil production. High trans fats intake for vanaspati, butter and fried oils. High erucic acid content in mustard oil. Potential source of excess omega 6 fatty acids. | Promote the responsible use of moderate quantities of oils and fats based on the cooking methods and localization. Encourage moderation but not exclusion in the use of ghee, palm and coconut oil due to their high saturated fat content and of mustard oil due to high erucic acid and contamination with argemone oil.  Limit vanaspati from diet due to the high trans fat content. Educate on the environmental impact of palm oil and promote sustainable alternatives. Promote the use of oils with a higher smoke point (ghee, coconut ghee, or peanut oil) for frying to reduce harmful compounds. Consider the use of fortified oils with additional vitamins and minerals. Use seeds and nuts as alternatives, when possible. | (11,26–29) |
| Legumes and Pulses | Lentils (Masoor Dal, Moong Dal, Toor Dal), Chickpeas, Black Gram (Urad Dal), Pigeon Peas, Green Gram, Kidney Beans (Rajma), Soy beans | **Soups/curries**  Dal Soup/Chana DalSoup/Urad Dal Soup/Usal Soup/Kootu Soup/Sambar, Chana Masala/Rajma Masala/Lobia Masala/Mulligatawny  **Main Dishes**  Haleem/Dhansak  **Side Dishes**  Dal Tadka/Kadhi, Urad Dal Fry, Dal ‘veggie balls’, Bara  **Salads**  Sprouted Dhal Salad/Chana Chaat/Lobia Salad/Rajma Salad/Masoor Dal Salad/Kala Chana Salad  **Sweets**  Besan Ladoo/Moong Dal Halwa/Chana Dal Barfi, PuranPoli  **Fried Foods/Snacks**  Pakoras/Samosa/Vada/Dal Kachori/Bhallas  **Breads**  Missi Roti/Thepla/Puran Poli/Dal Paratha/Besan Cheela  **Fermented**  Masyaura, Kinema/Kinama | High in protein, fiber, complex carbohydrates. Rich in B vitamins (except B12) and minerals (iron, magnesium, zinc). Contain antioxidants (polyphenols). | **Strengths**  Good source of plant-based protein, high in fiber, rich in vitamins and minerals. **Weaknesses**  Lack of vitamin B12, presence of antinutrients, may cause bloating. | Soaking legumes before cooking, use of dehulled or blended legumes, and incorporating digestive spices like cumin, coriander, and asafoetida (hing) can aid in digestion and reduce antinutrients content. Increase educational projects aimed at favoring legumes consumption and preservation of traditional recipes. Increase use of fermented legumes and sprout legumes for additional benefits. Reduction of recipes that require frying or use of added sugars. | (13–15) |
| Nuts/Seeds | Almonds, Peanuts, Cashews, Sesame Seeds, Sunflower Seeds, Chia Seeds | **Fried**  Biryani/Fried Rice/Ladoo/Groundnut Chikki/Gajar ka Halwa/Upma, Badam Halwa  **Boiled**  Chana Chaat/Sundal, Peanut Chikki  **Roasted**  Chapati  **Sweet Snacks**  Tilgul | Moderate protein content and high healthy fats (monounsaturated and polyunsaturated), fiber, vitamins (E, B2, B6, folate), minerals (magnesium, phosphorus, potassium, zinc, selenium, copper). | **Strengths**  Rich in healthy fats, protein, and fiber. Nutrient-dense and versatile in cooking. **Weaknesses**  High-calorie content,  Disproportion in n-6/n-3 PUFA in most variants.  Potential allergens (peanuts, tree nuts), risk of aflatoxin contamination in improperly stored nuts. | Promote the consumption of raw or roasted nuts without added salt or sugar. Encourage the use of nuts and seeds in combination with fruits, vegetables, and whole grains for balanced snacks. Increase awareness of portion sizes to avoid excessive calorie intake. Promote the use of chia and flax seeds as sources of omega-3 fatty acids. Encourage the consumption of a variety of nuts and seeds to benefit from different nutrient profiles. Consider fortifying nuts and seeds with additional vitamins and minerals. | (24,25) |
| Milk and Dairies | Milk, Paneer, Yogurt (Dahi), Ghee, Khoya | **Soft Cheese**  Paneer/Peda/Rasgulla/Chhena  **Yogurt and Similar (Curd)**  Dahi, Shrikhand/Rabri/Basundi  **Drinks**  *See ‘non alcoholic beverages’*  **Sweets**  Kulfi/Mishti Doi/Gulab, Khoya, Jamun/Rasmalai/Sandesh | High-quality protein, saturated fats, calcium, vitamins (A, D, B12), and minerals (phosphorus, magnesium, potassium). Fermented dairies contain probiotics. | **Strengths**  High protein content, rich in calcium and vitamins. Probiotics in fermented dairy.  **Weaknesses**  High in saturated fats and calories. Lactose intolerance concerns. High sodium in some products. | Increase consumption of low-fat and probiotic dairy products. Reduce added sugars in flavored yogurts. Promote lactose-free options. Encourage grass-fed and organic dairy. | (11,12) |
| Eggs | Chicken Eggs, Duck Eggs | **Fried**  Fried egg/Egg bhurji/Mughlai paratha/Egg bonda/Goan egg drop curry/Kerala egg roast, Egg Roll  **Baked**  Egg tadka/Egg korma  **Mixed Dishes**  Egg keema/Chettinad egg curry/Nargisi kofta egg curry, Egg Curry  **Street Food**  Egg Paratha Roll | High-quality protein, vitamins (A, D, E, B2, B6, B12), minerals (iron, zinc, phosphorus, selenium), antioxidants (lutein, zeaxanthin). | **Strengths**  High-quality protein, versatile in cooking.  **Weaknesses**  High cholesterol content, risk of contamination (salmonella), overconsumption of fried egg dishes. | Encourage boiling or poaching methods to reduce added fats. Promote the use of eggs in combination with vegetables for balanced meals. Increase awareness about the nutritional benefits of eggs.  Consider fortification of eggs with omega-3 fatty acids. | (22,23) |
| Ultra-Processed Foods and Sugar-Sweetened Beverages | Various processed ingredients, high fructose corn syrup, artificial flavors | **Snacks**  Packaged chips/crisps/instant noodles  **Soft Drinks**  Cola-based beverages/Lemon-based beverages/Fruit-flavored beverages  **Desserts**  Packaged cakes/cookies/pastries | High in calories, sugar, salt, unhealthy fats (saturated, hydrogenated, trans fats), and additives. Low fiber and antioxidant content. | **Strengths**  Convenient and widely available  **Weaknesses**  Contribute to obesity, diabetes, and other health issues. | Encourage natural food alternatives. Educate on reading labels and recognizing ultra-processed foods. | (40–42) |
| Alcoholic Beverages | Barley, Wheat, Rice, Grapes and other fruits, Palm Sap | **Beers**  Tongba(Millet beer), Rice Beer/Yu/Poko  **Wines**  Rice and cereal wines, palm wine, flower-based wines, grape (Angoori, Rguntshang) or other fruits wines (Banana wine, Apricot Wine, Jackfruit wine)  **Spirits**  Distilled spirits from cereals (Raksi/Chhang/Aara) palm (Toddy Arrack) or flowers  **Liqueurs**  Flower liqueurs (Mahua), Fruit liqueurs (Mead), nut liqueurs (Feni), palm liqueurs | Alcoholic beverages provide calories but negligible amounts of essential nutrients. Some beverages contain antioxidants (wine). | **Strengths**  Moderate consumption of some alcoholic beverages (e.g., wine) may have cardiovascular benefits. **Weaknesses**  High calorie content, potential for addiction and abuse, negative health impacts with excessive consumption. | Promote moderation and responsible consumption. Educate on the potential health risks associated with excessive alcohol intake. Encourage the consumption of beverages with lower alcohol content. | (37) |
| Non-Alcoholic Beverages | Tea Leaves, Herbs (Chamomile, Dandelion, Fennel, Honeybush, Oolong), Yogurt, Sugarcane, Lemon, Fruits | **Local Fruit Juices**  Aam Ras/Sugarcane Juice/Nariyal Pani/Anar ka Ras/Guava Juice/Pineapple Juice/Amla Juice/Pomegranate Juice, Lemon Sherbet  **Tea**  Chai/Green Tea/Herbal Teas (Chamomile, Dandelion, Fennel, Honeybush, Oolong), Masala Chai  **Milk or Curd-Based Drinks**  Lassi/Mattha/Mohi/Borhani/Ghol/Thandai, Buttermilk (Chaas/Moru)  Falooda, Sarbat | Non-alcoholic beverages vary in nutrient content; fruit juices provide vitamins and minerals, tea contains antioxidants, and curd-based drinks provide probiotics and essential nutrients | **Strengths**  Hydrating, variety of flavors and nutritional benefits depending on the beverage. **Weaknesses**  High sugar content in many beverages, potential for added artificial flavors, preservatives and chemical residuals | Promote the consumption of beverages with low or no added sugars. Encourage the use of natural ingredients in traditional beverages. Educate on the benefits of hydration and choosing healthier beverage options. Promote the use of herbal teas for their health benefits without adding calories. Encourage the consumption of homemade beverages to control ingredients and reduce sugar intake. | (38,39) |
| Ultra-Processed Foods and Sugar-Sweetened Beverages | Various processed ingredients, high fructose corn syrup, artificial flavors | **Snacks**  Packaged chips/crisps/instant noodles  **Soft Drinks**  Cola-based beverages/Lemon-based beverages/Fruit-flavored beverages  **Desserts**  Packaged cakes/cookies/pastries | High in calories, sugar, salt, unhealthy fats (saturated, hydrogenated, trans fats), and additives. Low fiber and antioxidant content. | **Strengths**  Convenient and widely available  **Weaknesses**  Contribute to obesity, diabetes, and other health issues. | Encourage natural food alternatives. Educate on reading labels and recognizing ultra-processed foods. | (40–42) |

**Summary of South Asian non-vegetarian diet typical food and recipes**

| Food Group | Raw Ingredients | Main Recipes | Raw Properties | Strengths and Weaknesses | Possible Improvements | References |
| --- | --- | --- | --- | --- | --- | --- |
| Rice | Basmati Rice, Jasmine Rice, Brown Rice, Red Rice, Black Rice | **Fermented Rice**  Dosa/Panta Bhat/Idli/Dhokla/Uttapam/Appam/Paniyaram/Bubur Cha Cha/Apong  **Flavored Rice**  Biryani/Nasi Lemak/Nasi Goreng/Pulao/Tahiri/Lamprais/Kiribath/Lemon Rice/Tamarind Rice, Tehari/Zafrani Pulao  **Flatbreads**  Akki roti  **Rice Soups**  Khichuri/Khichdi, Borhani/Congee  **Side Dishes**  Sambhar Rice/Curd Rice/Coconut Rice/Hoppers/Appam, Pittu/Puttu  **Fried Rice**  Sel Roti  **Sweet Rice**  Ricepudding/Kheer/Zarda/Lamprais/Kiribath/Yomari  **Puffed Rice**  Bhel Puri, Jhalmuri  **Rice Cakes**  Pitha | High in carbohydrates, low in fat, moderate in protein. Rich in B vitamins (B1, B3, B9). Contains iron, manganese, selenium. Fiber content varies with processing; whole rice retains more fiber and micronutrients than white rice. Whole ricehashigherantioxidantvalue. | **Strengths**  Versatile and staple in South Asian diets. Provides energy and sustenance. Fermented rice offers probiotics. **Weaknesses**  Refined rice has high glycemic index and low fiber. Often consumed with minimal vegetables or proteins. High oil and sugar content in some recipes. | Increase consumption of whole grains like brown, red, and black rice. Enrich meals with vegetables and legumes. Reduce added oils and sugars. | (1–3) |
| Wheat | Whole Wheat, Sharbati Wheat, Semolina, All-Purpose Flour | **Whole Wheat Flour (Atta) and Sharbati Wheat Flour:** Roti/Chapati/Paratha/Phulka/Punjabi Aloo Paratha/Atta Dosa/Missi Roti/Onion Paratha/Pav, Bhatura  **Semolina**  Idli/Dosa/Upma/Momos, Sheera  **All-Purpose Flour**  Cakes/Pastries/Cookies/Biscuits/Samosas/Appam/Gulgule/Nankhatai, Mawa cake  **Soups**  Wheat flour soup  **Flatbreads**  Naan, Kulcha | High in carbohydrates, protein, B vitamins (B1, B2, B3, B6, B9), and minerals (iron, magnesium, phosphorus). Whole wheat is rich in fiber. All-purpose flour has lower nutrient content due to refining. | **Strengths**  High nutrient density in whole wheat. Good source of energy and protein. Versatile in many recipes.  **Weaknesses**  Refined wheat has high glycemic index, lower fiber, and nutrient loss. High consumption of refined wheat products. | Increase consumption of whole wheat products. Use alternative grains like millet and sorghum. Reduce portions of refined wheat products. Promote biofortified wheat with higher zinc content. | (4,5) |
| Other grains | Jowar (Sorghum), Bajra (Pearl Millet), Ragi (Finger Millet), Maize | **Jowar**: Jowar Roti/Bhakri  **Bajra**: Bajra Roti, Bajra Khichdi, KambuKoozh  **Ragi**: Ragi Dosa/Ragi Mudde/Ragi Malt  **Maize**: Makki Roti/Chakki Roti | High in carbohydrates, fiber, protein, and essential minerals like calcium, iron, magnesium. Most are gluten-free | **Strengths**  Nutrient-dense and suitable for gluten-sensitive individuals. High in fibers. Promote microbiota diversity and quality.  **Weaknesses**  Can be less palatable compared to refined grains.  Presence of anti-nutrients in whole grains.  Shorter shelf life and baking quality compared to refined flours. Maize is naturally low in fiber and has high glycemic index. | Promote traditional recipes using millets and other grains, which can also be fortified for additional nutrient value. Encourage variety in grain consumption. | (6–10) |
| Tubers and starchy roots | Potato, Sweet Potato, Taro (Arbi), Yam/Elephant foot Yam (Orr),Cassava, Lotus Root, | **Main Courses**  Aloo Gobi, Sweet Potato Curry, Taro Curry/Kcamal Kakdi Curry/Orr Curry ,Chatpate, Chotpoti  **Side Dishes**  Aloo Paratha,  Sweet Potato fry/Cassava fry/Orr Fry, Arbi Masala, Kamal Kakdi Ki Subzi,  **Snacks**  Potato Pakoras, Sweet Potato Chaat, Taro Chips, Pani Puri/Golgappa/Aloo Tikki, Samosa | High in carbohydrates, fiber, vitamins (C, B6), minerals (potassium, magnesium, calcium). | **Strengths**  High energy content, rich in vitamins and minerals.  **Weaknesses**  High glycemic index, potential for high calorie content in fried preparations. | Promote the consumption of baked or boiled tubers instead of frying. Encourage the use of sweet potatoes and taro for their higher nutrient content compared to regular potatoes. Educate on portion control to manage calorie intake. Consider the use of spices and herbs to enhance flavor without adding excessive calories or salt. Promote the inclusion of tubers in combination with other vegetables for balanced meals. Encourage the use of tubers in soups and stews for a nutritious addition. | (34–36) |
| Vegetables | Okra, Drumstick, Drumstick Leaves, Eggplant (Baingan), Spinach, Bitter Gourd, Bottle Gourd, Carrot, Radish, Tomato, Cauliflower, Cabbage, Chayote, Pumpkin,  Bamboo shoots | **Main Courses** Vegetable Curry/Aloo Gobi/Baingan Bharta/Palak Paneer, Undhiyu/Eromba  **Side dishes** Achar/Raita/Aloo Paratha/Bhindi Masala, Chana Chaat, Vegetable Pakoras  **Salads**  Mixed fresh salads  **Pickles**  Pachranga Achar/Gongura pickle/Lal Mirch ka Bharwa Achar/DalleKhursani Achar/Pika pila  **Snacks**  Bhaji /Pakoras, Chana Chaat  **Fermented vegetables dishes**  Gundruk, Sinki, Masyaura | Rich in vitamins (A, C, K, folate), minerals (potassium, magnesium, calcium), fiber, antioxidants (polyphenols, flavonoids). Low on calories, proteins, fats. | **Strengths**  High nutrient density, rich in vitamins and minerals, fiber content supports digestive health.  **Weaknesses**  Potential for pesticide residue; variable nutrient content depending on preparation methods; overcooking can reduce nutrient content; presence of anti-nutrients in some cases | Promote the consumption of a variety of vegetables to ensure a broad spectrum of nutrients. Encourage the use of organic and locally sourced vegetables to reduce pesticide exposure. Educate on the benefits of minimal cooking methods to preserve nutrients. Promote the inclusion of vegetables in every meal for balanced nutrition. Encourage home gardening and foraging to increase access to fresh vegetables. Consider the use of biofortified vegetables to enhance nutrient content. | (14,18,30–33) |
| Fruits | Mango, Banana, Papaya, Pomegranate, Guava, Pineapple, Jackfruit, Apple, Grapes, Oranges, Watermelon, Melon, Chikoo, Lychee | **Local Fruit Juices**  Aam Ras/Sugarcane Juice/Nariyal Pani/Anar ka Ras/Guava Juice/Pineapple Juice/Amla Juice/Pomegranate Juice, Lemon Sherbet  **Traditional Desserts**  Mango Lassi, Banana Fritters, Papaya Halwa, Pomegranate Raita | Rich in vitamins (A, C, E, K), minerals (potassium, magnesium, calcium), fiber, antioxidants (polyphenols, flavonoids). | **Strengths**  High in vitamins and minerals, rich in antioxidants, hydrating properties.  **Weaknesses:**  Variable nutrient content depending on ripeness, potential for high sugar content in some fruits, pesticide residues. | Promote the consumption of a variety of fruits to ensure a broad spectrum of nutrients. Encourage the use of organic and locally sourced fruits to reduce pesticide exposure. Educate on the benefits of consuming whole fruits instead of fruit juices to retain fiber content. Promote the inclusion of fruits in every meal for balanced nutrition. Encourage home gardening to increase access to fresh fruits. Consider the use of biofortified fruits to enhance nutrient content. Encourage the use of fruits in combination with nuts and seeds for balanced snacks. | (14,18,30–33) |
| Herb/Spice | Black Pepper, Chili Pepper, Turmeric, Ginger, Garlic, Clove, Cinnamon, Cardamom, Saffron, MustardSeeds, Fennel, Coriander, Nutmeg, Fenugreek | **Curries:** Butter chicken, Masala curry, Korma  **Rice Dishes:** Biryani, Pulao  **Condiments:** Mustard, Chutneys, Hot sauces  **Soups:** Tom yum, Mulligatawny  **Teas:** Chai, Herbal infusions  **BakedGoods:** Spiced bread, Cookies, Cakes  **Beverages:** Golden milk, Saffron tea | Rich in bioactive compounds, essential oils, polyphenols, flavonoids, and minerals | **Strengths**  Antioxidant, anti-inflammatory, antimicrobial, digestive aid, metabolism support, cardiovascular health, and immunity effects.  **Weaknesses**  Pepper, chili and mustard may cause irritation in sensitive people.  Potential adulteration with heavy metals or presence of micotoxins | Encourage balanced use, avoid excessive consumption of strong spices, promote fresh over processed forms, and enhanced absorption (like black pepper with turmeric) | (43,44) |
| Oils and coking fats | Mustard oil, Coconut oil, Ghee, Coconut ghee, Sesame oil, Peanut oil, Palm oil, Sunflower oil,Soy bean oil, Rapeseed oil,  Vanaspati | **Tempering**  Dal Tadka  **Deep Frying** Samosa/Pakoras Shallow frying  Aloo Paratha  **Roasting**  Baingan Bharta  **Cooking in Oil-Based Gravies**  Rogan Josh, Chicken Karahi  **Seasoning Salads** Kachumber | High in fats (saturated, monounsaturated, polyunsaturated), vitamins (E), and antioxidants. Mustard oil: omega-3 and omega-6 fatty acids, vitamin E. Coconut oil: saturated fats, lauric acid, medium chain triglycerides. Ghee: saturated fats, vitamins (A, D, E, K). Sesame oil: unsaturated fats, antioxidants, vitamin E. Peanut oil: monounsaturated fats, vitamin E. Palm oil: saturated fats, vitamin E, carotenes.  Vanaspati: saturated fats and high amount of trans fats | **Strengths**  Rich in essential fatty acids, vitamin E, and antioxidants. Versatile in cooking methods. **Weaknesses**  High calorie and fat content, potential health risks with excessive consumption, environmental impact of palm oil production. High trans fats intake for vanaspati, butter and fried oils. High erucic acid content in mustard oil. Potential source of excess omega 6 fatty acids. | Promote the responsible use of moderate quantities of oils and fats based on the cooking methods and localization. Encourage moderation but not exclusion in the use of ghee, palm and coconut oil due to their high saturated fat content and of mustard oil due to high erucic acid and contamination with argemone oil.  Limit vanaspati from diet due to the high trans fat content. Educate on the environmental impact of palm oil and promote sustainable alternatives. Promote the use of oils with a higher smoke point (ghee, coconut ghee, or peanut oil) for frying to reduce harmful compounds. Consider the use of fortified oils with additional vitamins and minerals. Use seeds and nuts as alternatives, when possible. | (11,26–29) |
| Legumes and Pulses | Lentils (Masoor Dal, Moong Dal, Toor Dal), Chickpeas, Black Gram (Urad Dal), Pigeon Peas, Green Gram, Kidney Beans (Rajma), Soy beans | **Soups/curries**  Dal Soup/Chana DalSoup/Urad Dal Soup/Usal Soup/Kootu Soup/Sambar, Chana Masala/Rajma Masala/Lobia Masala/Mulligatawny  **Main Dishes**  Haleem/Dhansak  **Side Dishes**  Dal Tadka/Kadhi, Urad Dal Fry, Dal ‘veggie balls’, Bara  **Salads**  Sprouted Dhal Salad/Chana Chaat/Lobia Salad/Rajma Salad/Masoor Dal Salad/Kala Chana Salad  **Sweets**  Besan Ladoo/Moong Dal Halwa/Chana Dal Barfi, PuranPoli  **Fried Foods/Snacks**  Pakoras/Samosa/Vada/Dal Kachori/Bhallas  **Breads**  Missi Roti/Thepla/Puran Poli/Dal Paratha/Besan Cheela  **Fermented**  Masyaura, Kinema/Kinama | High in protein, fiber, complex carbohydrates. Rich in B vitamins (except B12) and minerals (iron, magnesium, zinc). Contain antioxidants (polyphenols). | **Strengths**  Good source of plant-based protein, high in fiber, rich in vitamins and minerals. **Weaknesses**  Lack of vitamin B12, presence of antinutrients, may cause bloating. | Soaking legumes before cooking, use of dehulled or blended legumes, and incorporating digestive spices like cumin, coriander, and asafoetida (hing) can aid in digestion and reduce antinutrients content. Increase educational projects aimed at favoring legumes consumption and preservation of traditional recipes. Increase use of fermented legumes and sprout legumes for additional benefits. Reduction of recipes that require frying or use of added sugars. | (13–15) |
| Nuts/Seeds | Almonds, Peanuts, Cashews, Sesame Seeds, Sunflower Seeds, Chia Seeds | **Fried**  Biryani/Fried Rice/Ladoo/Groundnut Chikki/Gajar ka Halwa/Upma, Badam Halwa  **Boiled**  Chana Chaat/Sundal, Peanut Chikki  **Roasted**  Chapati  **Sweet Snacks**  Tilgul | Moderate protein content and high healthy fats (monounsaturated and polyunsaturated), fiber, vitamins (E, B2, B6, folate), minerals (magnesium, phosphorus, potassium, zinc, selenium, copper). | **Strengths**  Rich in healthy fats, protein, and fiber. Nutrient-dense and versatile in cooking. **Weaknesses**  High-calorie content,  Disproportion in n-6/n-3 PUFA in most variants.  Potential allergens (peanuts, tree nuts), risk of aflatoxin contamination in improperly stored nuts. | Promote the consumption of raw or roasted nuts without added salt or sugar. Encourage the use of nuts and seeds in combination with fruits, vegetables, and whole grains for balanced snacks. Increase awareness of portion sizes to avoid excessive calorie intake. Promote the use of chia and flax seeds as sources of omega-3 fatty acids. Encourage the consumption of a variety of nuts and seeds to benefit from different nutrient profiles. Consider fortifying nuts and seeds with additional vitamins and minerals. | (24,25) |
| Milk and Dairies | Milk, Paneer, Yogurt (Dahi), Ghee, Khoya | **Soft Cheese**  Paneer/Peda/Rasgulla/Chhena  **Yogurt and Similar (Curd)**  Dahi, Shrikhand/Rabri/Basundi  **Drinks**  *See ‘non alcoholic beverages’*  **Sweets**  Kulfi/Mishti Doi/Gulab, Khoya, Jamun/Rasmalai/Sandesh | High-quality protein, saturated fats, calcium, vitamins (A, D, B12), and minerals (phosphorus, magnesium, potassium). Fermented dairies contain probiotics. | **Strengths**  High protein content, rich in calcium and vitamins. Probiotics in fermented dairy.  **Weaknesses**  High in saturated fats and calories. Lactose intolerance concerns. High sodium in some products. | Increase consumption of low-fat and probiotic dairy products. Reduce added sugars in flavored yogurts. Promote lactose-free options. Encourage grass-fed and organic dairy. | (11,12) |
| Eggs | Chicken Eggs, Duck Eggs | **Fried**  Fried egg/Egg bhurji/Mughlai paratha/Egg bonda/Goan egg drop curry/Kerala egg roast, Egg Roll  **Baked**  Egg tadka/Egg korma  **Mixed Dishes**  Egg keema/Chettinad egg curry/Nargisi kofta egg curry, Egg Curry  **Street Food**  Egg Paratha Roll | High-quality protein, vitamins (A, D, E, B2, B6, B12), minerals (iron, zinc, phosphorus, selenium), antioxidants (lutein, zeaxanthin). | **Strengths**  High-quality protein, versatile in cooking.  **Weaknesses**  High cholesterol content, risk of contamination (salmonella), overconsumption of fried egg dishes. | Encourage boiling or poaching methods to reduce added fats. Promote the use of eggs in combination with vegetables for balanced meals. Increase awareness about the nutritional benefits of eggs.  Consider fortification of eggs with omega-3 fatty acids. | (22,23) |
| Fish and seafood | Rohu, Hilsa, Pomfret, Kingfish, Mackerel, Sardines, Squid,  Crabs,  Octopus, Shrimps, Shellfish | **Fried Fish**  Machher Jhol/Meen Varuval/Amritsari Fish, Fish Fry  **Soups/Curries**  Fish Moilee/Patrani Machhi/Goan Fish Curry, Shrimp Curry/Crab Curry, Fish Stew  **Grilled Fish**  Tandoori Fish/Hariyali Fish Tikka, Grilled Pomfret  **Barbecue Fish**  **Raw Fish**  Fish Ceviche/Fish Carpaccio  **Pickled Fish**  Fish Achaar  **Fermented Fish**  Shidal/Ngari, HentakTungtap | High-quality protein, omega-3 fatty acids (in fish), vitamins (B2, B3, B6, B12, D), and minerals (iodine, iron, zinc, selenium). | **Strengths**  Important source of omega-3 fatty acids, vitamins, minerals and lean protein.  **Weaknesses**  High levels of contaminants in some fish, overfishing, and sustainability concerns. Common deep frying reduces nutritional content.  Dried fish is rich in salt. Traditional cooking methods can sometimes involve the use of heavy sauces and oils that increase the calorie content of fish dishes. | Promote grilling, baking, or steaming methods instead of frying to preserve the nutritional content. Increase awareness of sustainable fishing practices and promote the consumption of sustainably sourced fish. Encourage the inclusion of a variety of fish in the diet to benefit from different nutrient profiles. Consider seasonal availability and local sourcing to reduce environmental impact. Recommend moderation in the use of heavy sauces and oils in fish dishes to maintain their health benefits. Promote fish rich in omega-3s (such as mackerel and sardines) for heart health benefits. Encourage the use of spices and herbs for flavor instead of heavy sauces. Consider incorporating more fish-based soups and stews with vegetables for a balanced meal. | (19–21) |
| Meat | Chicken, Mutton, Beef, Pork, Lamb | **Red Meat Dishes**  Rogan Josh/Mutton Biryani/Keema Curry/MachherJhol/Bengali Fish Curry/Hyderabadi Biryani, Lamb Vindaloo  **Grilled**  Tandoori Chicken/Seekh Kebab/Fish Tikka  **Fried**: Kerala Beef Fry/Mutton Chops/Chicken 65/Amritsari Fish Fry, Samosa, Pakora  **Soups**  Nihari/Fish Curry/MeenRasam/Lamb Paya Soup/Goan Fish Curry Soup  **White Meat Dishes**  Chicken Curry/Butter Chicken/Chicken Tikka Masala/Tandoori Chicken/Chicken Pakora, Kori Gassi  **Street Food**  Kebab Paratha Rolls, Beef Seekh Kebab | High-quality protein, iron, zinc, selenium, B vitamins (B12, B6, niacin), and minimal carbohydrates. Fish also provide omega-3 fatty acids and vitamin D. | **Strengths**  Provides essential nutrients and high-quality protein. Culinary diversity with rich flavors.  **Weaknesses**  High saturated fat content in some meats. Production of carcinogenic compounds during cooking procedures. Potential contamination and spoilage risks. Environmental impact of meat production. | Promote alternative cooking methods such as grilling, baking, or steaming instead of deep-frying. Encourage the use of moderate amounts of healthy oils for cooking meat. Encourage the inclusion of a variety of vegetables in meat based dishes. Promote legumes, dairy, fish and eggs as alternative protein sources | (16–18) |
| Ultra-Processed Foods and Sugar-Sweetened Beverages | Various processed ingredients, high fructose corn syrup, artificial flavors | **Snacks**  Packaged chips/crisps/instant noodles  **Soft Drinks**  Cola-based beverages/Lemon-based beverages/Fruit-flavored beverages  **Desserts**  Packaged cakes/cookies/pastries | High in calories, sugar, salt, unhealthy fats (saturated, hydrogenated, trans fats), and additives. Low fiber and antioxidant content. | **Strengths**  Convenient and widely available  **Weaknesses**  Contribute to obesity, diabetes, and other health issues. | Encourage natural food alternatives. Educate on reading labels and recognizing ultra-processed foods. | (40–42) |
| Alcoholic Beverages | Barley, Wheat, Rice, Grapes and other fruits, Palm Sap | **Beers**  Tongba(Millet beer), Rice Beer/Yu/Poko  **Wines**  Rice and cereal wines, palm wine, flower-based wines, grape (Angoori, Rguntshang) or other fruits wines (Banana wine, Apricot Wine, Jackfruit wine)  **Spirits**  Distilled spirits from cereals (Raksi/Chhang/Aara) palm (Toddy Arrack) or flowers  **Liqueurs**  Flower liqueurs (Mahua), Fruit liqueurs (Mead), nut liqueurs (Feni), palm liqueurs | Alcoholic beverages provide calories but negligible amounts of essential nutrients. Some beverages contain antioxidants (wine). | **Strengths**  Moderate consumption of some alcoholic beverages (e.g., wine) may have cardiovascular benefits. **Weaknesses**  High calorie content, potential for addiction and abuse, negative health impacts with excessive consumption. | Promote moderation and responsible consumption. Educate on the potential health risks associated with excessive alcohol intake. Encourage the consumption of beverages with lower alcohol content. | (37) |
| Non-Alcoholic Beverages | Tea Leaves, Herbs (Chamomile, Dandelion, Fennel, Honeybush, Oolong), Yogurt, Sugarcane, Lemon, Fruits | **Local Fruit Juices**  Aam Ras/Sugarcane Juice/Nariyal Pani/Anar ka Ras/Guava Juice/Pineapple Juice/Amla Juice/Pomegranate Juice, Lemon Sherbet  **Tea**  Chai/Green Tea/Herbal Teas (Chamomile, Dandelion, Fennel, Honeybush, Oolong), Masala Chai  **Milk or Curd-Based Drinks**  Lassi/Mattha/Mohi/Borhani/Ghol/Thandai, Buttermilk (Chaas/Moru)  Falooda, Sarbat | Non-alcoholic beverages vary in nutrient content; fruit juices provide vitamins and minerals, tea contains antioxidants, and curd-based drinks provide probiotics and essential nutrients | **Strengths**  Hydrating, variety of flavors and nutritional benefits depending on the beverage. **Weaknesses**  High sugar content in many beverages, potential for added artificial flavors, preservatives and chemical residuals | Promote the consumption of beverages with low or no added sugars. Encourage the use of natural ingredients in traditional beverages. Educate on the benefits of hydration and choosing healthier beverage options. Promote the use of herbal teas for their health benefits without adding calories. Encourage the consumption of homemade beverages to control ingredients and reduce sugar intake. | (38,39) |
| Ultra-Processed Foods and Sugar-Sweetened Beverages | Various processed ingredients, high fructose corn syrup, artificial flavors | **Snacks**  Packaged chips/crisps/instant noodles  **Soft Drinks**  Cola-based beverages/Lemon-based beverages/Fruit-flavored beverages  **Desserts**  Packaged cakes/cookies/pastries | High in calories, sugar, salt, unhealthy fats (saturated, hydrogenated, trans fats), and additives. Low fiber and antioxidant content. | **Strengths**  Convenient and widely available  **Weaknesses**  Contribute to obesity, diabetes, and other health issues. | Encourage natural food alternatives. Educate on reading labels and recognizing ultra-processed foods. | (40–42) |

**REFERENCES**

1. Sudha V, Spiegelman D, Hong B, Malik V, Jones C, Wedick NM, Hu FB, Willett W, Bai MR, Ponnalagu MM, et al. Consumer Acceptance and Preference Study (CAPS) on Brown and Undermilled Indian Rice Varieties in Chennai, India. *J Am Coll Nutr* (2013) 32:50–57. doi: 10.1080/07315724.2013.767672

2. Kumar N, Chhokar RS, Meena RP, Kharub AS, Gill SC, Tripathi SC, Gupta OP, Mangrauthia SK, Sundaram RM, Sawant CP, et al. Challenges and opportunities in productivity and sustainability of rice cultivation system: a critical review in Indian perspective. *Cereal Res Commun* (2022) 50:573–601. doi: 10.1007/s42976-021-00214-5

3. Zhou Z, Robards K, Helliwell S, Blanchard C. Composition and functional properties of rice. *Int J Food Sci Technol* (2002) 37:849–868. doi: 10.1046/j.1365-2621.2002.00625.x

4. Šramková Z, Gregová E, Šturdík E. Chemical composition and nutritional quality of wheat grain. *Acta Chim Slovaca* (2009) 2:115–138.

5. Saini P, Kaur H, Tyagi V, Saini P, Ahmed N, Dhaliwal HS, Sheikh I. Nutritional value and end-use quality of durum wheat. *Cereal Res Commun* (2023) 51:283–294. doi: 10.1007/s42976-022-00305-x

6. Singhal T, Tara Satyavathi C, Singh SP, Mallik M, Anuradha N, Sankar SM, Bharadwaj C, Singh N. Achieving nutritional security in India through iron and zinc biofortification in pearl millet (Pennisetum glaucum (L.) R. Br.). *Physiol Mol Biol Plants* (2022) 28:849–869. doi: 10.1007/s12298-022-01144-0

7. Tako E, Reed SM, Budiman J, Hart JJ, Glahn RP. Higher iron pearl millet (Pennisetum glaucum L.) provides more absorbable iron that is limited by increased polyphenolic content. *Nutr J* (2015) 14:1–9. doi: 10.1186/1475-2891-14-11

8. Shobana S, Krishnaswamy K, Sudha V, Malleshi NG, Anjana RM, Palaniappan L, Mohan V. *Finger Millet (Ragi, Eleusine coracana L.). A Review of Its Nutritional Properties, Processing, and Plausible Health Benefits*. 1st ed. Copyright &copy; 2013 Elsevier Inc. All rights reserved. (2013). 1–39 p. doi: 10.1016/B978-0-12-410540-9.00001-6

9. Mal B, Padulosi S, Ravi SB. *Minor Millets in South Asia*. (2010). 1–185 p.

10. Pillam VSS, Siripurapu KK. The Bio - Cultural Diversity of Jowar / Sorghum. (2021).

11. Kumar A, Tripathi S, Hans N, Pattnaik F, Naik SN. Ghee: Its Properties, Importance and Health Benefits. *Lipid Universe* (2018) 6:1–14. https://www.researchgate.net/publication/339499398

12. Khan SU, Pal MA. Paneer production: A review. *J Food Sci Technol* (2011) 48:645–660. doi: 10.1007/s13197-011-0247-x

13. Amoah I, Ascione A, Muthanna FMS, Feraco A, Camajani E, Gorini S, Armani A, Caprio M, Lombardo M. Sustainable Strategies for Increasing Legume Consumption: Culinary and Educational Approaches. *Foods* (2023) 12:1–32. doi: 10.3390/foods12112265

14. Kapoor R, Sabharwal M, Ghosh-Jerath S. Indigenous Foods of India: A Comprehensive Narrative Review of Nutritive Values, Antinutrient Content and Mineral Bioavailability of Traditional Foods Consumed by Indigenous Communities of India. *Front Sustain Food Syst* (2022) 6: doi: 10.3389/fsufs.2022.696228

15. Shridhar K, Dhillon PK, Bowen L, Kinra S, Bharathi AV. Nutritional profile of Indian vegetarian diets – the Indian Migration Study (IMS). *Nutr J* (2014) 13:

16. Devi SM, Balachandar V, Lee SI, Kim IH. An outline of meat consumption in the indian population-A pilot review. *Korean J Food Sci Anim Resour* (2014) 34:507–515. doi: 10.5851/kosfa.2014.34.4.507

17. Sathyamala C. Meat-eating in India: Whose food, whose politics, and whose rights? *Policy Futur Educ* (2019) 17:878–891. doi: 10.1177/1478210318780553

18. Antani V, Mahapatra S. Evolution of Indian cuisine: a socio-historical review. *J Ethn Foods* (2022) 9: doi: 10.1186/s42779-022-00129-4

19. Fernandes AC, Medeiros CO, Bernardo GL, Vieira M, Faria P, Pietro DI, Altenburg De Assis MA, De Assis F, De Vasconcelos G. Benefits and risks of fish consumption for the human health. *Rev Nutr* (2012) 25:283–295.

20. Thapa N, Pal J, Tamang JP. Microbial diversity in ngari, hentak and tungtap, fermented fish products of North-East India. *World J Microbiol Biotechnol* (2004) 20:599–607. doi: 10.1023/B:WIBI.0000043171.91027.7e

21. Rifat MA, Wahab MA, Rahman MA, Nahiduzzaman M, Mamun A Al. Nutritional value of the marine fish in Bangladesh and their potential to address malnutrition: A review. *Heliyon* (2023) 9:e13385. doi: 10.1016/j.heliyon.2023.e13385

22. Morris SS, Beesabathuni K, Headey D. An egg for everyone: Pathways to universal access to one of nature’s most nutritious foods. *Matern Child Nutr* (2018) 14:1–9. doi: 10.1111/mcn.12679

23. Ruxton CHS, Derbyshire E, Gibson S. The nutritional properties and health benefits of eggs. *Nutr Food Sci* (2010) 40:263–279. doi: 10.1108/00346651011043961

24. Balakrishna R, Bjørnerud T, Bemanian M, Aune D, Fadnes LT. Consumption of Nuts and Seeds and Health Outcomes Including Cardiovascular Disease, Diabetes and Metabolic Disease, Cancer, and Mortality: An Umbrella Review. *Adv Nutr* (2022) 13:2136–2148. doi: 10.1093/advances/nmac077

25. George ES, Daly RM, Tey SL, Brown R, Wong THT, Tan SY. Perspective: Is it Time to Expand Research on “nuts” to Include “seeds”? Justifications and Key Considerations. *Adv Nutr* (2022) 13:1016–1027. doi: 10.1093/advances/nmac028

26. Awogbemi O, Onuh EI, Inambao FL. Comparative study of properties and fatty acid composition of some neat vegetable oils and waste cooking oils. *Int J Low-Carbon Technol* (2019) 14:417–425. doi: 10.1093/ijlct/ctz038

27. Mhatre S, Rajaraman P, Chatterjee N, Bray F, Goel M, Patkar S, Ostwal V, Patil P, Manjrekar A, Shrikhande S V., et al. Mustard oil consumption, cooking method, diet and gallbladder cancer risk in high- and low-risk regions of India. *Int J Cancer* (2020) 147:1621–1628. doi: 10.1002/ijc.32952

28. Manna S, Sharma HB, Vyas S, Kumar J. Comparison of mustard oil and ghee consumption on the history of coronary heart disease in urban population of India. *J Clin Diagnostic Res* (2016) 10:OC01–OC05. doi: 10.7860/JCDR/2016/18929.8593

29. Shahidi F. *Nutraceutical and Specialty Lipids and their Co-Products*. Shahidi F, editor. CRC Press. (2006). 579 p.

30. Jayawardena R, Jeyakumar DT, Gamage M, Sooriyaarachchi P, Hills AP. Fruit and vegetable consumption among South Asians: A systematic review and meta-analysis. *Diabetes Metab Syndr Clin Res Rev* (2020) 14:1791–1800. doi: 10.1016/j.dsx.2020.09.004

31. Bishwajit G, O’Leary DP, Ghosh S, Sanni Y, Shangfeng T, Zhanchun F. Association between depression and fruit and vegetable consumption among adults in South Asia. *BMC Psychiatry* (2017) 17:1–9. doi: 10.1186/s12888-017-1198-1

32. Lampe JW. Health effects of vegetables and fruit: Assessing mechanisms of action in human experimental studies. *Am J Clin Nutr* (1999) 70:475S-490S. doi: 10.1093/ajcn/70.3.475s

33. Bajgai RC, Bajgai Y, Johnson SB. The presence of wild edible plants and determinants influencing their harvest, consumption, and conservation in south eastern Bhutan. *PLoS One* (2023) 18:1–24. doi: 10.1371/journal.pone.0285936

34. Sreeramulu D, Raghunath M. Antioxidant activity and phenolic content of roots, tubers and vegetables commonly consumed in India. *Food Res Int* (2010) 43:1017–1020. doi: 10.1016/j.foodres.2010.01.009

35. Beals KA. Potatoes, Nutrition and Health. *Am J Potato Res* (2019) 96:102–110. doi: 10.1007/s12230-018-09705-4

36. Chattopadhyay A, Saha B, Pal S, Bhattacharya A, Sen H. Quantitative and qualitative aspects of elephant foot Yam. *Int J Veg Sci* (2009) 16:73–84. doi: 10.1080/19315260903211852

37. Swain MR, Kumari A, Pandey A, Gupta A. *Indigenous Alcoholic Beverages of South Asia*. (2015).

38. Sani AM, Rahbar M, Sheikhzadeh M. *Traditional beverages in different countries: Milk-based beverages*. (2019). 239–272 p. doi: 10.1016/B978-0-12-815504-2.00007-4

39. Rabade VS, Gurunani SG, Chaple DR. Appraising herbal tea as a medicated and nutritive drink. *Res J Pharm Technol* (2016) 9:613–616. doi: 10.5958/0974-360X.2016.00117.7

40. Parmar J. Sugar - sweetened beverages and their association with obesity in South Asian children. (2014).

41. Bren d’Amour C, Pandey B, Reba M, Ahmad S, Creutzig F, Seto KC. Urbanization, processed foods, and eating out in India. *Glob Food Sec* (2020) 25:100361. doi: 10.1016/j.gfs.2020.100361

42. Kalra S, Kalra B, Kapoor L, Kapoor N. Ultra Processed Foods: A South Asian culinary perspective. *J Pakistan Med Assoc* (2024) 74:

43. Bhathal SK, Kaur H, Bains K, Mahal AK. Assessing intake and consumption level of spices among urban and rural households of Ludhiana district of Punjab, India. *Nutr J* (2020) 19:1–12. doi: 10.1186/s12937-020-00639-4

44. Mackonochie M, Rodriguez-Mateos A, Mills S, Rolfe V. A Scoping Review of the Clinical Evidence for the Health Benefits of Culinary Doses of Herbs and Spices for the Prevention and Treatment of Metabolic Syndrome. *Nutrients* (2023) 15: doi: 10.3390/nu15234867
