# Supplementary material for: South Asia-specific adaptation of Mediterranean diet principles: a mixed-methods review for practical and sustainable dietary habits
Source: Front Nutr. 2025 Dec 23;12:1719686. doi: 10.3389/fnut.2025.1719686 (PMC12786337; doi:10.3389/fnut.2025.1719686)
Supplement: Supplementary file 8 [file Table_8.DOCX]

**Supplementary File 8.** **Integration of systematic and scoping evidence into final pyramid adaptation and policy recommendations**

| Evidence source | Key findings | Implications for pyramid adaptation | Required actions beyond the pyramid |
| --- | --- | --- | --- |
| **Systematic review — Protein intake and quality** | Protein intake was frequently below adequacy; pulses provided low-quality proteins with suboptimal digestibility; egg consumption was limited due to cultural norms; dairy emerged as the primary high-quality protein source for vegetarians; inland areas had minimal access to fish. | Increased emphasis on dairy and legumes for vegetarians; introduction of diversified protein options; weekly inclusion of fish, eggs, or poultry for non-vegetarians; recommendation to target ≥1 g/kg/day protein where feasible. | Policies to improve availability and affordability of dairy and pulses; support for small-scale aquaculture; nutrition education on amino acid profiles and protein quality. |
| **Systematic review — Cultural protein patterns** | Vegetarianism is widespread and strongly rooted in religious identity; egg consumption remains controversial in several communities; dairy is culturally anchored and widely accepted. | Development of two parallel pyramids (vegetarian and non-vegetarian); preservation of dairy as a central protein source; flexible inclusion of eggs depending on local norms. | Partnerships with religious/community leaders to support culturally acceptable nutrition messaging; adaptation of public programs to vegetarian households. |
| **Systematic review — Fat sources and fatty acid balance** | Diets showed excessive intake of n-6-rich oils and limited access to n-3 sources; commonly used refined oils contained fewer bioactive compounds; industrial trans-fat sources (e.g., vanaspati) were associated with increased cardiovascular risk. | Recommendation of mixed-oil strategies (mustard + canola/rapeseed + limited ghee/coconut); encouragement of ALA-rich seeds; explicit positioning of industrial trans-fat sources at the top of the pyramid (to be avoided). | Regulatory restrictions on trans fats; subsidies for oils with balanced fatty-acid profiles; promotion of perilla/camelina cultivation as alternative ALA sources. |
| **Systematic review — Carbohydrate sources** | High reliance on refined rice and wheat; insufficient whole-grain consumption; tubers showed comparable or superior fiber and micronutrient profiles and greater affordability. | Tubers positioned at the same level as grains due to nutritional quality and economic accessibility; emphasis on whole-grain substitution; reduction of refined cereals. | Agricultural incentives for whole grains and nutrient-dense tubers; public communication campaigns on the health value of non-refined staples. |
| **Scoping review — Affordability and modernization** | Healthy foods were often unaffordable; modernization increased access to industrial snacks; cost was a major determinant of refined cereal consumption. | Integration of affordability considerations in all food groups; nuts and seeds recommended in small amounts; fried snacks and processed sweets placed at the top. | Subsidies for legumes, nuts, and whole grains; regulation of highly processed foods; expansion of home and community food production initiatives. |
| **Scoping review — Cultural traditions** | Frying practices were central to culinary identity; sweets held symbolic and social importance; dairy was viewed as nourishing and culturally positive. | Inclusion of traditional sweets in moderation; allowance for frying with safer fats; maintenance of culturally meaningful dairy consumption. | Reformulation programs for traditional sweets; training on safer frying practices; community-led demonstrations of healthier cooking methods. |
| **Scoping review — Nutritional awareness** | Awareness of dietary inadequacies was low; misconceptions regarding food quality, protein needs, and fat sources were common. | Lifestyle block included at the base of the pyramids; clear guidance on balanced meals; visible reminders of hydration, rest, and physical activity. | School-based nutrition curricula; community workshops; mHealth and digital education campaigns; materials tailored to urban vs rural populations. |
| **Scoping review — Environmental and food safety constraints** | Widespread pesticide contamination, unsafe water, heavy metals in crops; climate variability reduced the availability of traditional foods like millets. | Emphasis on safe water; preference for foods less prone to contamination; inclusion of climate-resilient staples (e.g., millets, tubers). | Strengthening food safety regulations; investment in irrigation and storage infrastructure; promotion of agroecological practices. |
